# Stars, Stripes, and Perceptions: Examining the Impact of the American Flag on Anticipated Interactions

**DOI:** 10.3390/bs16101776

**Published:** 2026-09-28

**Authors:** Sheba M. Aikawa, Evava S. Pietri, Emily J. Brown, Katherine Parks

**Affiliations:** Department of Psychology & Neuroscience, University of Colorado Boulder, Muenzinger D244, 345 UCB, Boulder, CO 80309, USA

**Keywords:** American flag, identity threat, intergroup relations, national symbols, social dominance

## Abstract

The American flag is often associated with patriotism, solidarity, and national allegiance; however, it may not uniformly signal inclusion for all Americans. Across three studies (*N* = 1658), we examined whether the presence of the American flag in professional settings influences perceptions and anticipated interactions. Black, Latine, and White students viewed a Zoom background depicting a professor’s office containing an American flag (versus no flag or a sports team flag in Study 1; versus no flag or an inclusive flag in Study 2). Across both studies, participants perceived the professor displaying the American flag as more racist, more sexist, and higher in social dominance orientation (SDO), and Black/Latine (vs. White) participants reported less comfort and interest in interacting with the professor. In Study 3, we examined perceptions of the American flag in a workplace context. Replicating the previous studies, both Black and White participants viewed a coworker displaying the American flag (vs. no flag) as more racist, more sexist, and higher in SDO. Across all studies, these effects were generally stronger among participants lower in SDO. Together, these findings demonstrate how national symbols can shape anticipated interpersonal dynamics depending on perceiver identity and ideological orientation.

## 1. Introduction

Symbols can represent a common membership when they evoke strong feelings of identification (Butz, 2009; Callahan & Ledgerwood, 2016; Schatz & Lavine, 2007). In this way, these symbols can act as forces of integration, fostering solidarity by uniting individuals into a collective whole (Klatch, 1988). However, symbols can also create division within society (Klatch, 1988). In particular, political symbols can both reinforce and reflect broader power dynamics in society (Klatch, 1988). National flags, in particular, are especially potent political symbols that can simultaneously reinforce national sentiments and deepen social divisions (Billig, 1995).

In the United States (U.S.), the American flag—the primary national symbol—is widely displayed at sporting events, on homes, in schools, and on government buildings (Chan, 2017; Estrada et al., 2024; Ferguson & Hassin, 2007; Kemmelmeier & Winter, 2008; Skitka, 2005). Although displaying the American flag is often interpreted as signaling patriotism or national allegiance, the flag does not uniformly signal inclusion for everyone. For some marginalized individuals, the flag may also represent the nation’s historical and ongoing injustice, such as the enslavement of Black Americans and the genocide of Native Americans (Kemmelmeier & Winter, 2008). Most notably, to bring awareness to issues regarding racial injustice, police brutality, and the treatment of minorities in the U.S., NFL player Colin Kaepernick decided to kneel during the national anthem and stated, “*I am not going to stand up to show pride in a flag for a country that oppresses Black people and people of color. To me, this is bigger than football and it would be selfish on my part to look the other way. There are bodies in the street and people getting paid leave and getting away with murder.*” Thus, although the American flag can symbolize unity and national pride, it can also serve as a subtle reminder of both the positive and negative (i.e., historical and ongoing inequality) aspects of the nation’s history (Billig, 1995; Ferguson & Hassin, 2007). Because the flag carries this dual symbolism, its presence may function as a cue that shapes how individuals interpret a person who bears it. In the present research, we examine whether the presence of the American flag in professional settings (e.g., school or workplace settings) influences perceptions and anticipated interactions.

### 1.1. Perceptions of the American Flag and Others Displaying the Flag

People rapidly form impressions based on minimal cues, often assigning disproportionate weight to subtle environmental signals when inferring another person’s beliefs, values, and behaviors. Indeed, observers can form relatively accurate impressions even in the absence of in-person behavioral cues (Bacev-Giles & Haji, 2017). Even background symbols (e.g., a flag hanging in someone’s office) can shape judgments and influence how individuals perceive others. For example, mere proximity to group symbols (e.g., a Christian cross) has been shown to affect inferences about an individual’s traits, even when evaluators were explicitly told that the pairing between the person and the symbol was entirely random (Carlston & Mae, 2007). More recently, El-Alayli et al. (2026) found that an American flag displayed on a person’s social media profile led observers to perceive the individual as more prejudiced (i.e., racist and sexist), aggressive, and intolerant. Together, this work suggests that people use the American flag as an ideological cue, inferring the beliefs and values of the person displaying it. Accordingly, in the present research, we predict that an individual (i.e., a professor or coworker) who displays the American flag will be perceived as holding more prejudicial attitudes (i.e., racism and sexism) than an individual displaying comparison flags (i.e., a sports flag or inclusive flag) or no flag.

Given the historical context of displaying the American flag, as well as the typical person who takes pride in the flag (Carney et al., 2008; R. J. Goldstein, 1995), examining why observers might make these inferences is important. National flags have long symbolized patriotism, identity, and belonging (Cerulo, 1993; Estrada et al., 2024; Kariryaa et al., 2022). In the U.S., the American flag is often associated with values such as commitment to the country, selfless sacrifice, and solidarity (Kemmelmeier & Winter, 2008; Marvin & Ingle, 1999). For example, following the 11 September 2001 attacks, the flag became a highly salient symbol of unity and resilience, and was displayed widely across both public and private spaces (Bratta, 2009; Estrada et al., 2024; Skitka, 2005).

At the same time, the American flag has acquired additional social and political meanings. Research has linked the flag to perceptions of power, war, dominance, aggression (Ferguson & Hassin, 2007; Becker et al., 2017), pro-White bias (Devos & Banaji, 2005), and discrimination (Kemmelmeier & Winter, 2008). The American flag is further linked to nationalism, patriotism, and conservatism (Kemmelmeier & Winter, 2008; Skitka, 2005; Wilson & Sibley, 2013). Consistent with these perceptions, conservative (versus liberal) college students are more likely to display the American flag in their dorm rooms (Carney et al., 2008), suggesting group differences in identification with, and reaction to, the national flag (Ferguson & Hassin, 2007). More broadly, political conservatism as well as attitudes towards the American flag are also associated with Social Dominance Orientation (SDO; Pratto et al., 1994), or the extent to which individuals endorse a group-based hierarchy. Furthermore, exposure to the American flag has been linked to greater endorsement of both SDO and nationalism (Kemmelmeier & Winter, 2008). Thus, because the flag has become associated with these ideological beliefs, observers may use it as a heuristic when forming impressions of others, inferring that individuals who display the American flag are more likely to endorse hierarchical beliefs (i.e., higher SDO) as well as prejudicial attitudes (e.g., perceived sexism and racism).

### 1.2. Participant Race and Social Dominance Orientation (SDO) as Moderators of American Flag Perceptions

Importantly, environmental cues do not signal threat or inclusion uniformly across individuals. Rather, individual differences may moderate whether environmental symbols signal identity-threat or -safety (Pietri et al., 2025). In addition to impacting impressions of others displaying the American flag, the flag may also serve as a general cue signaling inclusion or exclusion based on individuals’ identities and personal ideologies (Park et al., 2023; Pietri et al., 2025). Marginalized individuals, in particular, attend closely to environmental cues when entering new settings to assess whether the context is identity-safe (i.e., free from the threat that one’s marginalized identity will lead to negative outcomes) or identity-threatening (i.e., where such threats are likely; Chaney et al., 2016; Davies et al., 2005; Kaiser et al., 2006; Kruk & Matsick, 2021; Oyserman et al., 2007; Pachankis, 2007; Park et al., 2023; Purdie-Vaughns & Walton, 2011; Richeson & Shelton, 2007; Steele et al., 2002; Vorauer, 2006). For instance, over 50% of Black individuals report experiencing microaggressions and racial slurs (Bleich et al., 2019). Relatedly, in a survey of 1000 undergraduate and graduate students, 44% of Black students and 34% of Latine students—compared to 30% of White students—reported witnessing or experiencing increased discrimination toward marginalized groups on campus within that past year (Nam, 2024). Such experiences of marginalization can lead to heightened vigilance in environments where further mistreatment may occur (Crocker & Major, 1989).

While this vigilance can be adaptive—helping individuals anticipate and avoid potential threats—it can also carry cognitive and emotional costs and may undermine performance and well-being (Kruk & Matsick, 2021; Oyserman et al., 2007; Pachankis, 2007). Experiencing identity-threat is associated with a range of negative outcomes in organizational and educational contexts, including burnout, disengagement, and attrition (Banchefsky et al., 2019; Burrows et al., 2022; Hall et al., 2015; Holleran et al., 2011). For example, prospective employees who anticipate not belonging in an organization are less attracted to the organization and less likely to pursue opportunities within the organization (Burrows et al., 2022; Pietri et al., 2019). Similarly, students with marginalized identities often encounter identity-threatening cues in higher education classrooms that signal the devaluation of their identities (Howansky et al., 2022; Petriglieri, 2011; Steele et al., 2002). These experiences can shape perceptions of instructors and lead to beliefs that an instructor either does or does not lack competence in addressing identity-related issues (Howansky et al., 2022; McKinney, 2005; Watkins et al., 2007). More broadly, various cues within classrooms or organizations can signal either inclusion or threat (Kruk & Matsick, 2021; Pietri et al., 2025), and these cues are often subtle, such as posters, decorations, or symbols.

The American flag may act as a subtle symbol evoking either identity-safety or identity-threat in an academic or organizational space, particularly for those who are attentive to identity-relevant cues. National flags represent group memberships and evoke strong emotional attachments to one’s nation; they are often mobilized to advance group goals or as part of collective nationalistic responses to outgroups (Becker et al., 2017; Butz, 2009). However, when individuals, particularly racially marginalized individuals, encounter the American flag in professional settings (e.g., schools or workplaces), they may interpret both the symbol and the individual displaying it as potential sources of threat. Specifically, the flag may function as a cue to group membership, from which observers infer ideological affiliations (e.g., political conservatism, nationalism; Carney et al., 2008; Ferguson & Hassin, 2007; Kemmelmeier & Winter, 2008; Skitka, 2005; Wilson & Sibley, 2013), potentially leading to expectations of prejudice (i.e., racism and sexism)—particularly among those who associate the flag with values that marginalize their group. In this way, national symbols not only define who belongs to the ingroup but also delineate who does not by emphasizing boundaries between “us” and “them” (e.g., *self-categorization theory*; Brewer, 1999; Butz, 2009; Callahan & Ledgerwood, 2016; Cerulo, 1993; Schatz & Lavine, 2007; Turner et al., 1987). Consequently, engaging with national symbols may prompt individuals to consider both inclusion and exclusion (Trofimchuk & Goh, 2024), leading observers to form impressions of the individual displaying the symbol and shaping their anticipated comfort and interest in interacting with that person.

Despite this possibility, little research has examined racial differences in perceptions of individuals displaying the American flag or whether national flags act as an identity-threat or -safety cue. Trofimchuk and Goh (2024) found relatively few racial differences in how positively or negatively Black and White Democrats evaluated the American flag overall. However, Black Democrats were more likely than White Democrats to associate the American flag with racism (Trofimchuk & Goh, 2024). Building on this work, the present research examines how the presence of the American flag shapes perceptions (i.e., perceived racism, sexism, SDO) and anticipated interactions (comfort and interest in interacting) among Black, Latine, and White individuals across the political spectrum.

In addition to participant race, individuals’ ideological orientations may shape responses to the American flag. One particularly relevant ideological difference is SDO. Individuals higher in SDO tend to hold more negative attitudes towards outgroups, and high-status group members are more likely to justify inequality (Sidanius & Pratto, 1999). Consistent with this perspective, individuals higher in SDO are also more likely to endorse nationalism (Osborne et al., 2017), and exposure to the American flag has been associated with greater endorsement of both nationalism and SDO (Kemmelmeier & Winter, 2008). Extending this prior research, the present studies examine both participant race and participants’ levels of SDO as moderators of responses to the American flag. Specifically, we predicted that the threatening effects of displaying the American flag on perceptions of the individual would be stronger among Black/Latine participants than White participants and stronger among participants lower in SDO than those higher in SDO. That is, these groups are expected to report more negative perceptions of the individual displaying the flag and less comfort and interest in interacting with them.

## 2. Overview of the Studies

Across three experiments, we examined whether the presence of the American flag influences perceptions (i.e., perceived racism, sexism, SDO) and anticipated interactions (i.e., comfort and interest in interreacting). In Studies 1 and 2, undergraduate students viewed a Zoom background picture of a professor’s office. In Study 1, the office either contained an American flag, a flag for a popular sports team (Denver Broncos), or no flag on the wall. In Study 2, the office either contained no flag, an American flag, or an inclusive “All Are Welcome Here” flag. Study 1 used participants recruited from a university subject pool, whereas Study 2 recruited a national racially diverse sample of undergraduates from Prolific. In Study 3, we shifted the context to a workplace setting, where participants viewed a Zoom background picture of a coworker’s office with an American flag present or absent.

We hypothesized that (H1) participants assigned to the American flag (vs. no flag, a Denver Broncos flag, and an inclusive flag) condition would report more negative perceptions of, and less comfort and interest in interacting with, the professor and coworker (Studies 1–3); (H2) negative responses to the American flag would be stronger for participants lower versus higher in SDO (Studies 1–3); (H3) negative responses to the American flag would similarly be stronger for Black/Latine versus White participants (Studies 2 and 3); and (H4) an inclusive flag would be perceived more positively by Black/Latine versus White participants (Study 2) (See Figure 1).

## 3. Study 1

In a non-preregistered study, we examined undergraduate participants’ perceptions of a Zoom background picture of a professor’s office. Participants were randomly assigned, in a between-subjects design, to view a professor’s office displaying no flag (true control), a Denver Broncos flag (control), or an American flag. We predicted that participants assigned to the American flag condition, compared to those in the no flag and Denver Broncos flag conditions, would report more negative perceptions of the professor (e.g., racism, sexism, SDO), lower comfort and interest in interacting with the professor, and a lower likelihood of selecting the professor (H1). Additionally, in an exploratory analysis, we examined whether these effects were moderated by participants’ level of Social Dominance Orientation (Participant SDO; i.e., preference for a group-based hierarchy), hypothesizing that the effects would be stronger among participants lower (versus higher) in SDO (H2).

### 3.1. Method

#### 3.1.1. Sensitivity Analysis and Participants

We recruited as many participants as possible during one semester at a western university in the U.S., resulting in 576 participants. After excluding participants who failed attention checks (e.g., a flag attention check and an open-ended attention check), the final sample included 560 participants (*M*_age_ = 18.92, *SD*_age_ = 2.12; 74% White; 73% women; 35 [6.3%] identified as extremely liberal, 199 [35.5%] identified as mostly liberal, 107 [19.1%] identified as somewhat liberal, 132 [23.6%] identified as neither liberal nor conservative, 62 [11.1%] identified as somewhat conservative, 23 [4.1%] identified as mostly conservative, 2 [0.4%] identified as extremely conservative). Participants who failed the attention checks did not vary systematically across conditions (see Supplemental Table S1). A post hoc sensitivity analysis conducted using G*Power (version 3.1.9.6; Faul et al., 2007) determined we were adequately powered to find small effect sizes (*f* = 0.13) at 80% power and *p* < 0.05. Additional details regarding attention checks, exclusions, and demographic information for all studies are provided in the Supplementary Materials (see Tables S1 and S2).

#### 3.1.2. Procedure and Measures

Information about the study measures—including example items, number of items, and scale endpoints—across all three experiments is reported in Table 1. Reliabilities and correlations for all the outcomes used in Study 1 are presented in Table 2. All materials, data, and syntax are available on Study 1’s OSF page: https://osf.io/n2a85/overview?view_only=7cf6ae79ec31441bbe14d39b56ef6fa7 (accessed on 13 August 2026).

We advertised the studies as examining “Perceptions of Zoom Backgrounds”, and participants were told that we were interested in understanding how people evaluate different Zoom backgrounds. After providing consent, participants were told that they would examine the Zoom background of Dr. Johnson, PhD’s (a fictitious professor) office. Moreover, Dr. Johnson holds office hours via Zoom, and this is the Zoom background that students see. Participants were then randomly assigned (via Qualtrics) to view one of three Zoom background pictures of Dr. Johnson’s office with: no flag (a blank background), a Denver Broncos flag, or an American flag. Importantly, Dr. Johnson was not depicted, and no sociodemographic information (e.g., race, gender) was provided.^1^ The Zoom background pictures are available on the OSF page.

Immediately after viewing their assigned Zoom background picture, participants completed an attention check to identify their assigned flag (or no flag); only those who answered correctly were included in analyses.^2^ Participants then completed measures assessing their perceptions of Dr. Johnson, including *perceived racism* and *sexism* (Sanchez et al., 2017) and perceived social dominance orientation (*professor SDO*; modified from Sanchez et al., 2017). Participants also described their general impressions of Dr. Johnson in an open-ended response. This item served as an attention check, allowing us to ensure participants were engaging meaningfully with the survey.

Next, participants were asked to imagine they were Dr. Johnson’s student and report their *comfort and interest* in interacting with Dr. Johnson (Pietri et al., 2018) and willingness to select Dr. Johnson as their professor (*professor selection*; modified from Aikawa et al., 2026).^3^ Following the primary outcomes, participants reported their own social dominance orientation (*participant SDO*;^4^ Kteily et al., 2011). The study concluded with demographic questions (e.g., race, country of origin, and political orientation; see Table S2 in the Supplementary Materials), followed by debriefing.

### 3.2. Results

To examine the influence of the flags on perceptions of the professor, we conducted separate one-way ANOVAs (no flag vs. Denver Broncos flag vs. American flag) for each dependent variable. Means, standard deviations, contrasts between conditions, and full ANOVA results are reported in Table 3. Bonferroni corrections were applied to account for multiple comparisons.

We found significant effects for all outcome variables (all *ps* < 0.001; see Table 3). Compared to the no flag condition, the Denver Broncos flag condition led to greater comfort and interest in interacting with the professor (*p* < 0.001) and a greater likelihood of selecting the professor (*p* < 0.001). There were no differences between the no flag and Denver Broncos flag conditions for perceived racism, perceived sexism, and professor SDO (all *ps* > 0.072; see Table 3).

In contrast, compared to both the no flag and the Denver Broncos flag conditions, displaying an American flag led to higher perceptions of racism, sexism, and professor SDO (*ps* < 0.001; see Table 3). Additionally, compared to the Denver Broncos flag, the American flag led to less comfort and interest in interacting with the professor (*p* < 0.001) and a lower likelihood of selecting the professor (*p* < 0.001; see Table 3). There were no differences between the no flag and American flag conditions for comfort and interest or professor selection (all *ps* > 0.115; see Table 3).

#### Moderation by Participant Social Dominance Orientation (SDO)

Next, we explored whether participants reported social dominance orientation (SDO) moderated the effects of the flag condition. Thus, we ran a regression model with condition dummy coded and the no flag condition as the reference group, Participant SDO, and all interactions.^5^ Initial analyses revealed no significant Flag Condition × Participant SDO interactions for the comparison between the no flag and Denver Broncos flag conditions (all *ps* > 0.094; see Table 4). However, we did observe significant Flag Condition × Participant SDO interactions for the comparison between the no flag and American flag conditions on all outcome variables (all *ps* < 0.001; see Table 4). We therefore probed the significant Flag Condition (no flag vs. American flag) × Participant SDO interactions by examining the effect of the no flag versus the American condition at one standard deviation below and above the mean for participant SDO (also see Table 4 for simple effects).

For comfort and interest, at one standard deviation below the mean on Participant SDO, participants expressed more comfort and interest interacting with the professor in the no flag compared to American flag condition (*p* = 0.001). The opposite pattern occurred for participants at one standard deviation above the mean on Participant SDO, with participants expressing more comfort and interest interacting with the professor in the American flag versus no flag condition (*p* = 0.011; see Table 4 and Figure 2).

Among participants at one standard deviation below (*ps* < 0.001) and above (*ps* < 0.023; see Table 4) the mean on participant SDO, compared to the no flag condition, they viewed the professor in the American flag condition as more racist, more sexist, and higher in SDO, but these effects were larger for participants with lower SDO scores (see Table 4).

Finally, for professor selection, among participants at one standard deviation below the mean on Participant SDO, they were more likely to select the professor in the no flag compared to American flag condition (*p* = 0.001). Participants at one standard deviation above the mean on Participant SDO did not show this effect (*p* = 0.226; see Table 4). Across all studies, we ran similar moderation analyses with political orientation. These analyses showed a similar pattern as moderation by Participant SDO and thus are presented in the Supplementary Materials.

### 3.3. Discussion

Study 1 served as an initial study to examine whether the American flag functioned as a cue impacting impressions of the professor. Consistent with H1, compared to both the no flag and Denver Broncos flag conditions, the presence of the American flag led participants to perceive the professor as more racist, more sexist, and higher in SDO. Contrary to H1, however, the American flag and no flag conditions did not significantly differ in participants’ comfort and interest in interacting with or selecting the professor. Interestingly, and contrary to predictions, the Denver Broncos flag produced a different pattern of effects. Relative to the no flag condition, the Broncos flag increased comfort and interest in interacting with the professor and the likelihood of selecting the professor, without increasing perceptions of racism, sexism, or SDO. This suggests that nonpolitical, culturally familiar symbols may serve as affiliative cues that enhance interpersonal warmth without signaling threat. The absence of effects on identity-based threat (i.e., racism and sexism) distinguishes the Denver Broncos flag from the American flag and underscores the unique social meaning of national symbols.

Moderation analyses further revealed that reactions to the American flag were not uniform, but instead varied as a function of individual differences in SDO. Aligning with H2, participants lower in SDO generally exhibited stronger negative reactions to the American flag, including heightened perceptions of racism, sexism, and SDO, as well as reduced comfort and interest in interacting with the professor and less willingness to select the professor. In contrast, these effects were attenuated—and, in some cases, reversed or nonsignificant—among participants higher in SDO. Indeed, among participants high in SDO, relative to the no flag condition, the American flag condition increased comfort and interest in interacting with the professor, suggesting that the American flag may function as an identity-safety cue for participants high in SDO. Notably, moderation effects were absent when comparing the no flag and Denver Broncos flag conditions, further reinforcing the idea that the observed individual differences are specific to the sociopolitical meaning of the American flag rather than to flags more generally.

## 4. Study 2

Given that the university subject pool sample in Study 1 was drawn from a single institution and consisted primarily of White and women participants, Study 2 recruited a more nationally diverse sample of undergraduate students. Specifically, we recruited Black, Latine, and White participants via Prolific. Additionally, Study 1 indicated that the American flag increased perceptions of identity-based threat (i.e., racism and sexism). Accordingly, Study 2 incorporated an identity-based safety symbol—an inclusive “All Are Welcome Here” flag—given prior research demonstrating that such cues can enhance feelings of belonging and safety among marginalized groups (Chaney & Sanchez, 2018; Maimon et al., 2023; Purdie-Vaughns et al., 2008).

This study was preregistered on OSF: https://osf.io/qwxc6/overview?view_only=ef8c686f16d6485bbd3bf749b4abffee (accessed on 4 April 2024). Based on Study 1, we hypothesized that Black, Latine, and White participants assigned to the American flag condition, relative to those assigned to the no flag and inclusive flag conditions, would perceive the American flag as a threatening cue. Consequently, we expected that participants would report lower comfort and interest in interacting with the professor, a lower likelihood of selecting the professor, and report more negative perceptions (i.e., racism, sexism, SDO) of the professor in the American flag condition relative to the other two conditions (H1). We further expected that these effects would be stronger among Black/Latine participants compared to White participants (H3) and among individuals lower in SDO relative to those higher in SDO (H2). In contrast, we anticipated that the inclusive flag would be perceived more positively by Black/Latine participants (relative to White participants), given that Black and Latine students tend to experience greater identity-threat in academic settings than White students (H4; Davis et al., 2024; Howansky et al., 2022).

### 4.1. Method

#### 4.1.1. Sensitivity Analysis and Participants

This study had a 2 (participant race: Black/Latine vs. White) × 3 (flag condition: no flag vs. inclusive flag vs. American flag) between-subjects design. We recruited 600 Black, Latine, and White students from Prolific, compensating participants $4.00. Due to exclusions (e.g., not identifying as Black, Latine, White; not identifying as a current student; and failing attention checks), we had a final sample of 542 participants (M_age_ = 22.01, SD_age_ = 1.95; 46% White; 54% Black/Latine; 53% women; 113 [20.8%] identified as extremely liberal, 156 [28.8%] identified as mostly liberal, 85 [15.7%] identified as somewhat liberal, 100 [18.5%] identified as neither liberal nor conservative, 51 [9.4%] identified as somewhat conservative, 28 [5.2%] identified as mostly conservative, 9 [1.7%] identified as extremely conservative). Participants who failed the attention checks did not vary systematically across conditions (see Supplemental Table S1). A post hoc sensitivity analysis conducted using G*Power (Faul et al., 2007) determined we were adequately powered to find small effect sizes (*f* = 0.13) at 80% power and *p* < 0.05.

#### 4.1.2. Procedure and Measures

All study materials, data, and syntax are available on Study 2’s OSF page: https://osf.io/n2a85/overview?view_only=7cf6ae79ec31441bbe14d39b56ef6fa7 (accessed on 13 August 2026). Reliabilities and correlations for all outcome variables are presented in Table 5. Study 2 followed the same procedure as Study 1, with two changes. First, we recruited a national racially diverse sample of Black, Latine, and White students from Prolific. Second, participants were randomly assigned (via Qualtrics) to view a Zoom background of Dr. Johnson’s office that displayed no flag, an American flag, or an inclusive “All Are Welcome Here” flag. Participants then completed the same attention checks and outcome measures as in Study 1.

### 4.2. Results

To examine whether participant race influenced perceptions across flag conditions, we conducted separate 2 (participant race) × 3 (flag condition) between-subjects ANOVAs for each dependent variable. See Table 6 for means and standard deviations and Table 7 for contrasts between conditions and full ANOVA results. Bonferroni corrections were applied to account for multiple comparisons.

First, for comfort and interest in interacting with the professor, there were significant main effects of participant race (*p* = 0.001) and flag condition (*p* < 0.001; see Table 7). White participants reported greater comfort and interest in interacting with the professor than Black/Latine participants. The inclusive flag increased comfort and interest compared to both the no flag and American flag conditions (*ps* < 0.001), which did not differ (*p* = 0.078; see Table 7). These effects were qualified by a significant participant race by flag condition interaction (*p* = 0.009; see Table 7). In the American flag condition, White participants reported greater comfort and interest than Black/Latine participants (*mean difference* = 0.91, *standard error* = 0.21, *p* < 0.001, *d* = 0.59; see Figure 3), but this effect was not significant in the inclusive flag (*mean difference* = 0.13, *standard error* = 0.21, *p* = 0.526, *d* = 0.11) or no flag conditions (*mean difference* = 0.14, *standard error* = 0.21, *p* = 0.509, *d* = 0.10).^6^

For perceived racism and sexism, there was a significant main effect of the flag condition (*ps* < 0.001; see Table 7). The American flag increased perceptions of racism and sexism relative to both the no flag and inclusive flag conditions (*ps* < 0.001). Moreover, the inclusive flag led to lower perceptions of racism and sexism than the no flag condition (*ps* < 0.001). Neither the main effect of participant race (all *ps* > 0.106) nor the interactions (all *ps* > 0.058; see Table 7) were significant.

For perceived professor SDO, there were significant main effects of participant race (*p* = 0.038) and flag condition (*p* < 0.001; see Table 7). Black/Latine participants perceived the professor as higher in SDO than White participants. The American flag increased perceived SDO relative to both the no flag and inclusive flag conditions (*ps* < 0.001), and the inclusive flag reduced perceived SDO compared to the no flag condition (*p* < 0.001). The interaction was not significant (*p* = 0.221; see Table 7).

Finally, for professor selection, there was a significant main effect of flag condition (*p* < 0.001; see Table 7). Participants were more willing to select the professor when the inclusive flag was displayed compared to when both no flag and the American flag were displayed (*ps* < 0.001), which did not differ (*p* = 0.095). The main effect of participant race (*p* = 0.268) and the interaction (*p* = 0.261) were not significant (see Table 7).

#### Moderation by Participant Social Dominance Orientation (SDO)

To examine whether participant SDO moderated the effects of the flag condition, similar to Study 1, we ran a regression model with the flag condition dummy coded and the no flag condition as the reference group, Participant SDO, and all interactions. Initial analyses revealed significant Flag Condition × Participant SDO interactions for the comparison between the no flag and inclusive flag conditions on all outcome variables (all *ps* < 0.001; see Table 8). Moreover, we found significant Flag Condition × Participant SDO interactions for the comparison between the no flag and American flag conditions for comfort and interest (*p* = 0.001), professor SDO (*p* = 0.044), and professor selection (*p* = 0.012; see Table 8). We did not find significant Flag Condition (No flag vs. American flag) × Participant SDO interactions for perceived racism and sexism (all *ps* > 0.351; see Table 8).

When interactions were significant, we examined the effect of the flag condition at one standard deviation above and below the mean on Participant SDO (see Table 8). When examining the simple effects of the Flag condition (No flag vs. Inclusive flag) on comfort and interest in interacting with the professor, participants reported more comfort and interest in the inclusive flag versus no flag condition at both one standard deviation below (*p* < 0.001) and above (*p* = 0.009) the mean on Participant SDO; however, the effect was stronger for participants lower in SDO (see Table 8). At low levels of Participant SDO, participants perceived the professor in the no flag versus inclusive flag condition as more racist and sexist (*ps* < 0.001); these effects were not significant for participants high in SDO (*ps* > 0.123; see Table 8). For professor SDO, participants perceived the professor in the no flag versus inclusive flag condition as higher in SDO at both one standard deviation below (*p* < 0.001) and above (*p* < 0.001) the mean on Participant SDO; however, the effect was stronger for participants lower in SDO (see Table 8). For professor selection, at low levels of Participant SDO, participants were more willing to select the professor in the inclusive flag versus no flag condition (*p* < 0.001); this effect was not significant for participants high in SDO (*p* = 0.168; see Table 8).

Next, when examining the simple effects of Flag Condition × Participant SDO interactions for the comparison between the no flag and American flag conditions, at low levels of Participant SDO, participants reported more comfort and interest in interacting with the professor (*p* < 0.001; see Figure 4) and more willingness to select the professor (*p* < 0.001) in the no flag versus American flag condition; these effects were not significant for participants high in SDO (*ps* > 0.677; see Table 8). Finally, for professor SDO, participants perceived the professor in the American flag versus no flag condition as higher in SDO at both one standard deviation below (*p* < 0.001) and above (*p* < 0.001) the mean on the Participant SDO scale; however, the effect was stronger for participants lower in SDO (see Table 8).

### 4.3. Discussion

Consistent with Study 1, in Study 2, the American flag functioned not merely as a neutral national symbol, but as a socially and ideologically loaded cue that impacted perceptions of the professor. Supporting H1 and replicating Study 1, the presence of the American flag increased perceptions of racism, sexism, and SDO. However, contrary to H1 and consistent with Study 1, the American flag and no flag conditions did not significantly differ in participants’ comfort and interest in interacting with or likelihood of selecting the professor. Consistent with H4, the inclusive “All Are Welcome Here” flag produced consistently more positive evaluations among not only Black/Latine participants, but also White participants, as well. These positive evaluations included greater comfort and interest in interacting with the professor and a higher likelihood of selecting the professor. Notably, the inclusive flag also reduced perceptions of racism, sexism, and SDO. Together, these findings suggest that symbols do not merely signal neutrality; rather, they actively shape expectations about interpersonal treatment and comfort (Callahan & Ledgerwood, 2016).

Critically, these effects were qualified by participant race. Black/Latine participants generally reported lower comfort and interest and perceived the professor as higher in SDO than White participants. Moreover, we only found a significant Participant Race by Flag Condition interaction for comfort and interest, indicating that the racial differences in perceptions emerged most strongly when the American flag was present but were attenuated in the inclusive flag and no flag conditions, partially aligning with H3.

Furthermore, consistent with H2 and Study 1, participants lower in SDO generally exhibited stronger negative reactions to the American flag, including heightened perceptions of SDO, reduced comfort and interest in interacting with the professor, and a reduced likelihood of selecting the professor. Unlike Study 1, in Study 2, a Flag Condition (No Flag vs. American Flag) by Participant SDO interaction did not emerge for perceived racism and sexism. Finally, in Study 2, using a new comparison flag, we found that participants lower in SDO generally exhibited stronger *positive* reactions to the inclusive flag, including heightened perceptions of comfort and interest in interacting, more willingness to select the professor, and reduced perceptions of racism, sexism, and SDO. Importantly, for both Flag Condition × Participant SDO interaction comparisons, these effects were attenuated—and, in some cases, reversed or nonsignificant—among participants higher in SDO. These findings provide initial evidence that responses to national and inclusive symbols are shaped not only by individuals’ broader ideological orientations toward hierarchy and inequality (i.e., SDO) but also by racial identity.

## 5. Study 3

Studies 1 and 2 demonstrated that the presence of the American flag can function as a threatening cue in university settings, particularly among Black/Latine students and students low in SDO. In Study 3, we shifted the context from a university setting to a workplace setting by examining perceptions of a coworker who displayed the flag, thereby providing a more stringent test of whether the effects of the American flag generalize across professional environments.

Study 3 was preregistered on OSF: https://osf.io/utkxc/overview?view_only=34d2bd5b2e614e3082885e001ad82959 (accessed on 1 July 2024). We predicted that participants assigned to the American flag condition, compared to those in the no flag condition, would perceive the coworker more negatively (i.e., as more racist, more sexist, and higher in SDO) and report less comfort and interest in interacting with them (H1). We further expected that these effects would be stronger among individuals lower in SDO compared to those with higher SDO scores (H2), and among Black participants than White participants (H3). In addition, we explored whether these effects varied across three timepoints (4 July, mid-2024 Summer Olympics, and post-2024 Summer Olympics) in order to examine whether the Olympics and 4 July would moderate our findings. Contrary to expectations, we did not find any significant effects of timepoint; however, we may have been underpowered to find the effect.^7^ These timepoint results are reported in the Supplementary Materials.

### 5.1. Method

#### 5.1.1. Participants

This study had a 2 (participant race: Black vs. White) × 2 (flag condition: no flag vs. American flag) between-subjects design. We recruited 600 Black and White adults from Prolific, compensating participants $4.00. After exclusions, the final sample included 556 participants (*M*_age_ = 37.50, *SD*_age_ = 12.51; 50% White; 50% Black; 50% women; 88 [15.8%] identified as extremely liberal, 106 [19.1%] identified as mostly liberal, 89 [16.0%] identified as somewhat liberal, 142 [25.5%] identified as neither liberal nor conservative, 57 [10.3%] identified as somewhat conservative, 48 [8.6%] identified as mostly conservative, 26 [4.7%] identified as extremely conservative). Details regarding attention checks, exclusions across conditions, and demographic information are provided in the Supplementary Materials (see Tables S1 and S2). A post hoc sensitivity analysis conducted using G*Power (Faul et al., 2007) determined we were adequately powered to find small effect sizes (*f* = 0.12) at 80% power and *p* < 0.05.

#### 5.1.2. Procedure and Measures

The materials, data, and syntax are available on Study 3’s OSF page: https://osf.io/rfzh4/overview?view_only=a28957d8f7404947a7e5c4f7e787875f (accessed on 13 August 2026). Reliabilities and correlations for all outcome variables are presented in Table 9. After providing consent, participants were informed that they would view the Zoom background of a (fictitious) coworker, Alex Johnson. Participants were told that the job is hybrid and that coworkers frequently meet with Alex via Zoom; the picture shown was the Zoom background of Alex’s office that coworkers see during meetings. Participants were then randomly assigned (via Qualtrics) to view either a Zoom background picture of Alex’s office with no flag (a blank background) or an American flag. Similar to the previous studies, Alex Johnson was not depicted, and no sociodemographic characteristics were specified.^8^ Participants then completed the same attention checks used in the previous studies and reported their perceptions of Alex’s *perceived racism* and *sexism* (Sanchez et al., 2017), perceived social dominance orientation (*coworker SDO*; modified from Sanchez et al., 2017), and *comfort and interest* in interacting with Alex (Pietri et al., 2018). Participants also provided their general impressions of Alex in an open-ended response. Finally, participants reported their own social dominance orientation (*participant SDO*; Kteily et al., 2011).^9^ The study concluded with demographic questions and debriefing.

### 5.2. Results

Across measures, we conducted separate 2 (participant race) × 2 (flag condition) between-subjects ANOVAs (see Table 10 and Table 11 for means, standard deviations, contrasts between conditions, and full ANOVA results). We found a significant main effect of flag condition on all outcome variables (all *ps* < 0.001) except for comfort and interest in interacting with the coworker (*p* = 0.122; see Table 11). When the coworker displayed the American flag (versus no flag), participants perceived the coworker as more racist, more sexist, and higher in SDO (*ps* < 0.001; see Table 11). We did not find a main effect of participant race (all *ps* > 0.087) nor significant interactions (all *ps* > 0.128) across all outcomes.

#### Moderation by Participant Social Dominance Orientation (SDO)

To examine whether Participant SDO moderated the flag condition, we ran a regression model with the flag condition (0 = no flag and 1 = American flag), Participant SDO, and all interactions. We found significant Flag Condition × Participant SDO interactions for all outcome variables (all *ps* < 0.001; see Table 12), and thus, we probed those interactions and examined the effect of the flag condition at low (one standard deviation below the mean) and high (one standard deviation above the mean) levels of Participant SDO.

Showing similar patterns as Study 1, at one standard deviation below the mean on Participant SDO, participants in the American flag condition indicated less comfort and interest in interacting with the coworker, compared to those in the no flag condition (*p* = 0.001; see Table 12). In contrast, at one standard deviation above the mean on Participant SDO, the opposite effects emerged. Participants in the American flag condition reported more comfort and interest in interacting with the coworker relative to those in the no flag condition (*p* = 0.049; see Table 12 and Figure 5).

Additionally, participants at one standard deviation below the mean on Participant SDO perceived the coworker in the American versus no flag condition as more racist (*p* < 0.001) and more sexist (*p* < 0.001), and these effects were not significant for participants at one standard deviation above the mean on Participant SDO (*ps* > 0.063; see Table 12). Finally, at one standard deviation below (*p* < 0.001) and above (*p* = 0.024) the mean on Participant SDO, participants viewed the coworker as higher in SDO in the American flag versus no flag condition; however, this effect was stronger for participants lower in SDO (see Table 12).

### 5.3. Discussion

Consistent with Study 1, Study 2, and H1, displaying the American flag increased perceptions of racism, sexism, and SDO, indicating that observers inferred hierarchy-enhancing beliefs from its presence. These findings suggest that even within a workplace context, where norms of professionalism might be expected to constrain negative judgments, participants who noticed the American flag used it as an ideological cue when forming impressions of the coworker, highlighting the potency of subtle environmental cues in guiding social judgements (Callahan & Ledgerwood, 2016; Purdie-Vaughns & Walton, 2011).

Surprisingly, unlike Studies 1 and 2, we did not find the predicted (H1) main effect of the flag condition for comfort and interest in interacting with the coworker, and contrary to Study 2, we did not find a main effect of participant race, nor any hypothesized Participant Race by Flag Condition interactions (H3), on any of the outcome variables. This divergence suggests that while the American flag reliably influenced perceptions of bias-related constructs (e.g., racism, sexism, SDO), these inferences did not translate into broader interpersonal evaluations within workplace settings.

Importantly, consistent with H2, moderation analyses revealed that responses to the American flag varied as a function of Participant SDO. Participants lower in SDO generally reacted more negatively to the American flag (vs. no flag), perceiving the coworker as more racist, more sexist (aligning with Study 1), and higher in SDO, and reported less comfort and interest in interacting with the coworker (aligning with both Studies 1 and 2). In contrast, participants higher in SDO showed attenuated—or, in some cases, reversed—patterns. Overall, these findings demonstrate that the effects of the American flag on negative perceptions generalize beyond academic settings to workplace environments. However, unlike in the academic context, these perceptions did not extend to comfort and interest in interacting with the coworker, suggesting that workplace norms may constrain the extent to which individuals express negative interpersonal judgments, even when they infer problematic beliefs.

## 6. Internal Mega-Analysis

To explore the consistency of the effects of the flag condition and the Participant Race by Flag Condition interaction across studies, we conducted an internal mega-analysis (e.g., Cooper & Patall, 2009; Curran & Hussong, 2009). Specifically, we ran multilevel models with flag condition and participant race as fixed factors and study as a random effect with random intercepts (see Table 13). Analyses focused on the outcome measures that were consistent across all three studies: comfort and interest, perceived racism, perceived sexism, and professor/coworker SDO. Across all outcomes, we found a significant main effect of flag condition (all *ps* < 0.001; see Table 13). Compared with the comparison flags condition (i.e., no flag, Denver Broncos flag, inclusive flag), participants in the American flag condition perceived the target person (i.e., professor and coworker) as more racist, more sexist, and higher in SDO, and reported less comfort and interest in interacting with them. Moreover, we only found a significant main effect of participant race for comfort and interest (*p* = 0.022; see Table 13), such that Black/Latine versus White participants reported less comfort and interest in interacting with the target person. Finally, we only found a significant Flag Condition by Participant Race interaction for comfort and interest (*p* = 0.005; see Table 13). Specifically, in the American flag condition, Black/Latine participants reported less comfort and interest in interacting with the target person than White participants. In contrast, in the comparison flags condition, comfort and interest did not significantly differ between Black/Latine and White participants (*p* = 0.706; see Table 13).

### General Discussion

The present research examined whether the American flag—one of the most ubiquitous national symbols in the U.S.—functions as a neutral background symbol or as a meaningful social cue that shapes interpersonal perceptions. Across Studies 1–3, the presence of the American flag consistently increased perceptions that a professor or coworker was more racist, more sexist, and higher in SDO, supporting H1. These effects emerged in both educational (Studies 1 and 2) and workplace (Study 3) contexts, suggesting that the social meanings attached to the flag generalize across settings. Consistent with H2, participants lower in SDO generally reacted more negatively to the American flag. Relative to participants higher in SDO, they perceived a professor and coworker displaying the American flag as more racist, more sexist (Studies 1 and 3), and higher in SDO. They also reported less comfort and interest in interacting with these individuals (Studies 1–3) and were less willing to select the professor (Studies 1 and 2). In contrast, participants higher in SDO showed attenuated—and, in some cases, reversed—responses to the American flag.

Beyond individual differences in SDO, the present research provides initial evidence that these negative reactions may be stronger among Black and Latine students than White students. Consistent with H3, Black/Latine (vs. White) students in Study 2 reported less comfort and interest in interacting with the professor displaying the American flag. However, contrary to H3, participant race did not influence perceptions of the American flag in a workplace context in Study 3. Finally, the inclusive flag in Study 2 led to more positive perceptions of the professor among not only Black/Latine participants (supporting H4), but also White participants, as well. Together, these findings align with prior work demonstrating that the American flag is associated with nationalism, dominance, and an intergroup hierarchy (Kemmelmeier & Winter, 2008; Ferguson & Hassin, 2007). Extending this literature, the present research shows that these associations are not only internalized but are also projected onto others who display the flag, shaping interpersonal judgments and anticipated interactions.

## 7. Theoretical Implications

These findings contribute to several theoretical perspectives on social perception, intergroup relations, and cues that spark feelings of identity-threat and -safety. First, across all three studies, the findings provide strong support for research demonstrating that subtle environmental cues can shape person perception (Callahan & Ledgerwood, 2016). Even in the absence of direct behavioral information, participants who noticed and evaluated the presence of a background symbol drew meaningful inferences about the person displaying it, underscoring the power of minimal cues in guiding social judgments. Notably, the current research extends this literature by demonstrating a national symbol specifically (i.e., the American flag) impacts these perceptions. The American flag appears to signal not only national identity but is also associated with ideological inferences, leading observers to infer beliefs, attitudes, and behaviors about the person displaying it (Castano et al., 2023).

The current findings also highlight the importance of considering perceiver characteristics when examining responses to national symbols. In particular, the results provide some evidence that reactions to the American flag vary across racial groups. In Study 2, Black/Latine participants reported lower comfort and interest in interacting with the professor than White participants, particularly when the professor displayed an American flag. This finding is consistent with research on identity-threat, which shows that marginalized individuals are especially attentive to environmental cues that signal whether a context is safe or threatening (Chaney et al., 2016; Davis et al., 2024; Steele et al., 2002). Given the historical and ongoing associations between the American national identity and racial hierarchy (Devos & Banaji, 2005), the flag may function as a threatening cue for some individuals, prompting heightened vigilance and negative expectations.

Importantly, however, this racial difference did not replicate in Study 3. Participant race did not significantly moderate responses to the American flag on any outcome, and the effects of the flag were limited to bias-related perceptions (e.g., racism, sexism, SDO). Unlike Studies 1 and 2, the American flag did not significantly influence interpersonal evaluations/feelings relevant to identity-safety and -threat, such as comfort and interest in interacting with the target. However, our internal mega-analysis across studies revealed a significant Flag Condition by Participant Race interaction for comfort and interest. Across the combined data, Black/Latine participants reported less comfort and interest than White participants when the target displayed the American flag, whereas no racial difference emerged in the comparison flags condition. This aggregate pattern suggests that racial differences in anticipated interpersonal interactions may be more consistent than the Study 3 results alone indicate. The nonsignificant interaction in Study 3 may therefore reflect, in part, the lower statistical power of an individual study to detect a potentially modest interaction effect. Nevertheless, additional adequately powered research is needed to establish the robustness of this effect. Together, these findings suggest that the social meaning of the American flag is specifically shaped by perceptions of the target’s ideological beliefs, whereas its influence on anticipated interpersonal interactions may be smaller and more difficult to detect across individual studies.

Differences in samples and settings may also help to explain variation across individual studies. Studies 1 and 2 recruited college students who evaluated a professor in an academic setting, whereas Study 3 recruited a broader adult sample who evaluated a coworker in a workplace setting. These differences are noteworthy because student samples differ in meaningful ways from general adult samples (e.g., age, education; Hanel & Vione, 2016). Thus, although the mega-analysis suggests an overall racial difference in interpersonal responses, the magnitude of this effect may depend on context. For example, racial differences in responses to the American flag may be more pronounced in educational settings among students than in workplace settings among adults. Additional research is needed to determine the conditions under which these differences emerge.

Another possibility concerns the target’s role and relative power. In Studies 1 and 2, participants evaluated a professor whom they were explicitly instructed to imagine is their instructor (e.g., a person with authority over their grades, evaluations, and academic opportunities). In contrast, the target in Study 3 was a coworker occupying a relatively equal-status role with no direct authority over participants. Notably, the only outcome effect that failed to replicate in Study 3 was that involving anticipated interpersonal interactions (comfort and interest), whereas inferences about the target’s beliefs (i.e., perceived racism, sexism, and SDO) remained. This pattern suggests that although participants consistently infer ideological characteristics from the display of the American flag, those inferences translate into interpersonal concern primarily when the target occupies a position of authority or power. This interpretation is consistent with research that finds that power and authority can be an important cue for those at risk of experiencing social identity-threat (Park et al., 2018). Future research should directly manipulate the target’s role and authority while holding the broader context constant to determine whether authority influences perceptions of the target when displaying the American flag.

Finally, consistent with prior work linking SDO to intergroup attitudes (Sidanius & Pratto, 1999), participants lower in SDO also exhibited stronger negative reactions to the presence of the American flag. Notably, in Study 2, these individuals responded especially positively to the inclusive flag, which increased positive perceptions, whereas the American flag heightened perceptions of inequality and threat. In contrast, participants higher in SDO showed attenuated patterns of responses. This divergence aligns with research indicating that ideological orientations shape how individuals interpret political symbols (Chan, 2017; Wilson & Sibley, 2013). Symbols associated with hierarchy (e.g., the American flag in this context) may be perceived as affirming existing social hierarchies, whereas inclusive symbols may be seen as challenging them. Interestingly, in Studies 1 and 3, participants low in SDO expressed less comfort and interest in interacting with the target displaying the American flag (relative to the target with no flag), whereas participants higher in SDO indicated more comfort and interest for the target associated with the American flag. Together, these findings suggest that the same environmental cue may function as either an identity-threat cue or an identity-safety cue depending on the perceiver. Rather than having universally fixed meanings, environmental symbols appear to be interpreted through the lens of social identities and ideological beliefs. More broadly, these results show the importance of considering individual differences when examining how environmental symbols shape perceptions of belonging, threat, and anticipated interpersonal interactions.

## 8. Practical Implications

This research has important implications for educational and organizational settings. Seemingly neutral or universally positive environmental cues, such as the display of a national flag, can shape perceptions of comfort, bias, and anticipated treatment—particularly for marginalized individuals, as these symbols may carry historical and contemporary associations with exclusion. Although the American flag improved interpersonal evaluations for participants high in SDO, it consistently was harmful for those low in SDO. Thus, individuals may consider how others (particularly their students or coworkers) may react when displaying national symbols. While such symbols may be helpful for some, they may run the risk of being harmful to others. These perceptions may, in turn, influence individuals’ willingness to engage, collaborate, or pursue opportunities within these environments. However, the inclusion of an explicit general signal of inclusion (e.g., the “All Are Welcome Here” flag) may mitigate these effects and foster more inclusive environments.

Notably, in Study 2, the inclusive flag increased positive perceptions among both White and Black/Latine participants who were lower in SDO, while neither benefiting nor harming White participants who were higher in SDO. This suggests that safety cues can promote inclusion without negatively impacting majority group members. More broadly, creating environments that support diverse identities may benefit all individuals, not only those from marginalized groups (Gurin et al., 2002; Howansky et al., 2022). Thus, institutions should carefully consider how environmental symbols are interpreted by diverse audiences. Efforts to promote inclusion may benefit from not only removing potentially ambiguous or exclusionary cues but also actively incorporating signals that communicate belonging and equity.

## 9. Limitations and Future Research

Several limitations should be noted. First, while the samples were racially and nationally diverse, they were predominantly liberal. Future research should examine more politically representative samples to better capture the full range of responses to the American flag. Importantly, the possibility that national symbols may also function as identity-threatening cues for White Americans, particularly those who identify with political ideologies that conflict with the values they associate with the flag, warrants further investigation. The American flag is often associated with patriotism, nationalism, and conservatism (Carney et al., 2008; Kemmelmeier & Winter, 2008), and these associations may make the symbol less identity-safe for White Americans who identify as liberal. For these individuals, the flag may signal an ideological or social environment that conflicts with their values or suggests potential interpersonal differences, even in the absence of racial marginalization. Consistent with this possibility, among the predominantly White participants in Study 1, exploratory analyses indicated that participants at one standard deviation below the mean on the Participant Political Orientation scale (1 = *Extremely Liberal* to 7 = *Extremely Conservative*) reported less comfort and interest in interreacting with the professor and were less likely to select the professor in the American flag versus no flag condition; and these effects were not significant for participants one standard deviation above the mean (see Supplementary Materials for full results). Second, additional research is needed to explore how other identities (e.g., ethnicity, immigration status) and contextual factors (e.g., political climate or geographic region) shape perceptions of national symbols.

Third, the study was presented as an investigation of “Perceptions of Zoom Backgrounds”, and participants were explicitly instructed to evaluate the background. Consequently, the flag became a focal object of attention rather than an incidental environmental cue. This design may have produced larger effect sizes than would occur under more naturalistic conditions and may have heightened participants’ attention to potentially threatening cues, particularly among individuals higher in vigilance (e.g., Black/Latine participants and those lower in SDO). Although this does not threaten internal validity, because all participants received identical instructions across studies, it may limit the construct and external validity of the findings. Importantly, the inclusion of the Denver Broncos flag condition in Study 1 partially addresses this concern by demonstrating that merely making a flag of any kind salient is insufficient to produce the same observed effects of the American flag. Future work should examine whether similar findings emerge when participants are not explicitly instructed to attend to the environment or when the flag is encountered incidentally.

Fourth, Study 3 was initially designed as a 3 (timepoint) × 2 (participant race) × 2 (flag condition) between-subjects experiment. To simplify the design, we removed the inclusive flag condition. In retrospect, however, the no flag condition may not have been the strongest control because differences could partially reflect responses to a personalized office versus a relatively blank office rather than to the American flag itself. Future studies should include additional nonpolitical comparison flags to better isolate the symbolic meaning of the American flag.

Lastly, participants were not provided with explicit sociodemographic information about the professor or coworker (e.g., race or gender). However, we did ask participants about their perceptions of the professor and coworker’s race, gender, and age range. Across all three studies, most participants inferred that the professor or coworker was a White man (see Supplementary Materials Table S3; see also the Supplementary Materials for results examining whether responses to the flag differed based on participants’ perceptions of the target). This pattern raises the possibility that the American flag amplified a preexisting stereotype associated with the target—a “see-American-flag–think-White-man” effect—such that the flag and the inferred identity reinforced one another. Notably, participants’ spontaneous convergence on a White male target, despite the absence of demographic information, is itself consistent with evidence that the national category “American” is more strongly associated with White Americans than with other racial groups (Devos & Banaji, 2005). Future research should directly test this mechanism by experimentally manipulating the target’s race and gender. If the observed effects reflect a flag–target congruence, displaying the American flag alongside a woman or a racially marginalized target (vs. a White male target) may attenuate—or even reverse—inferences of bias, instead eliciting perceptions of belonging or patriotism rather than dominance. Such findings would help distinguish whether the American flag independently generates these inferences or primarily amplifies preexisting stereotypes associated with the person displaying it.

## 10. Conclusions

In conclusion, the present research demonstrates that the American flag is not merely a passive symbol of national identity but an active social cue that shapes how individuals perceive and evaluate others. Across contexts, the flag signaled ideological meaning and evoked perceptions of bias and hierarchy, particularly among individuals more attuned to issues of inequality. Together, these findings highlight the powerful role of symbols in influencing social perception and underscore the importance of thoughtfully considering how everyday environmental symbols shape experiences of belonging, inclusion, and threat.

## Figures and Tables

**Figure 1:**
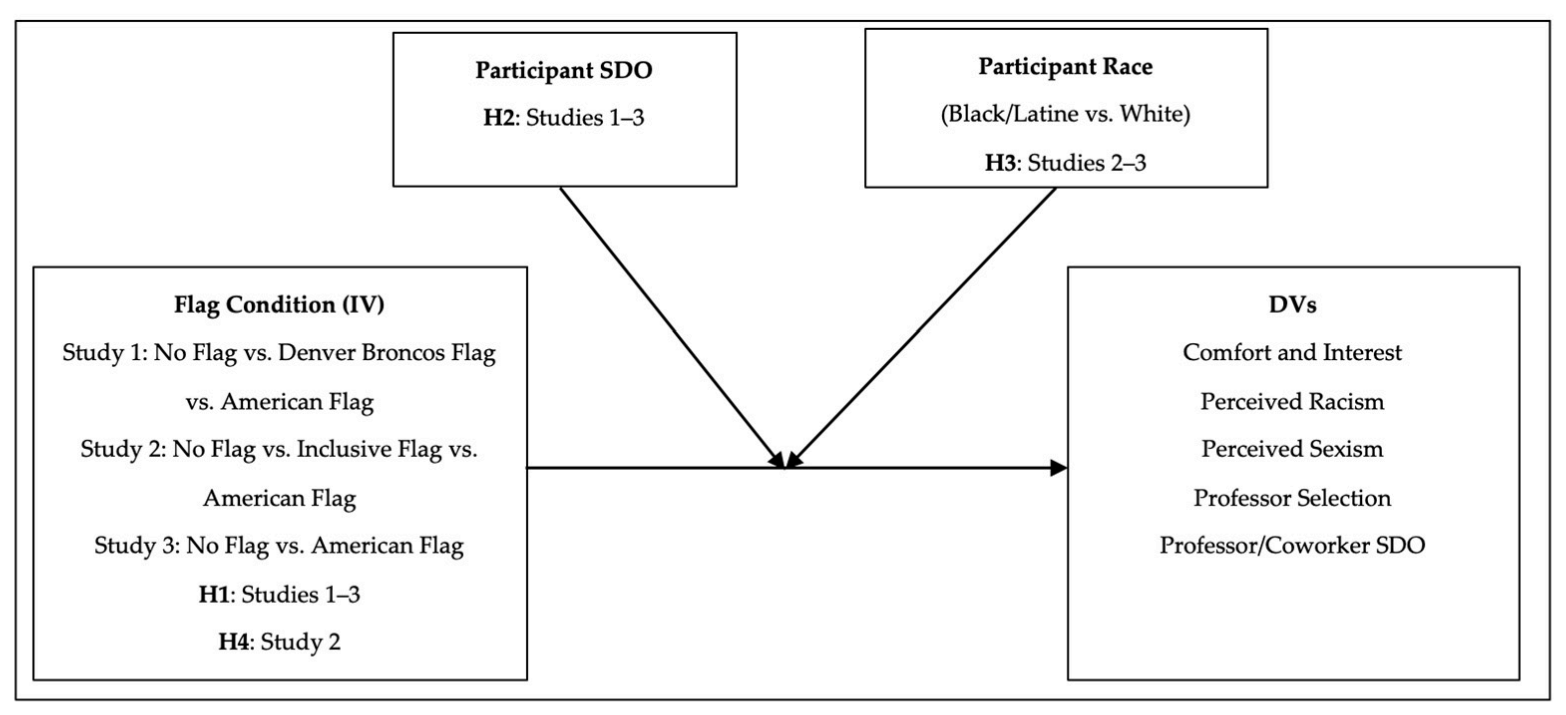
A conceptual model of the flag condition, moderators, and hypothesized effects across Studies 1–3). **Note:** Across all studies, we included a measure of perceived target SDO as an outcome variable (*Professor* [Studies 1 and 2] and *Coworker* [Study 3] *SDO*) and a measure of participants’ SDO as a moderator (*Participant SDO*).

**Figure 2:**
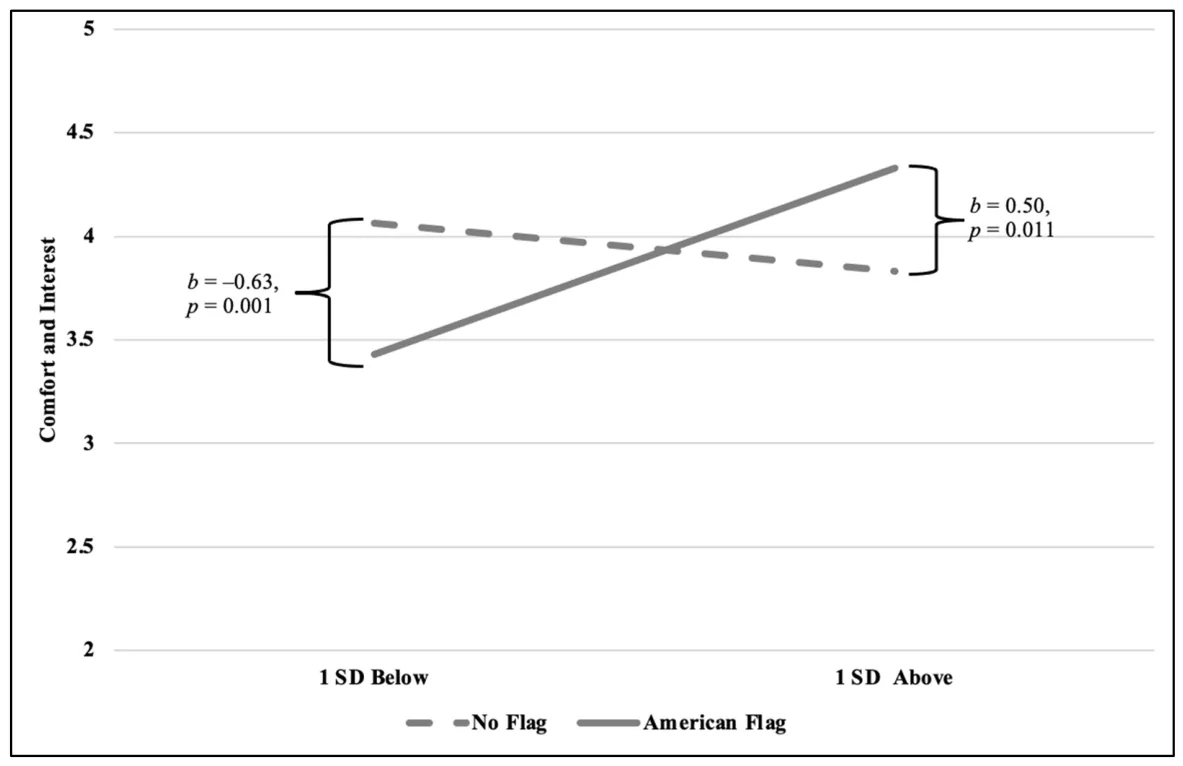
Study 1 simple effects of flag condition (no flag vs. American flag) and Participant SDO predicting Comfort and Interest at 1 standard deviation below and above the mean of the Participant SDO scale.

**Figure 3:**
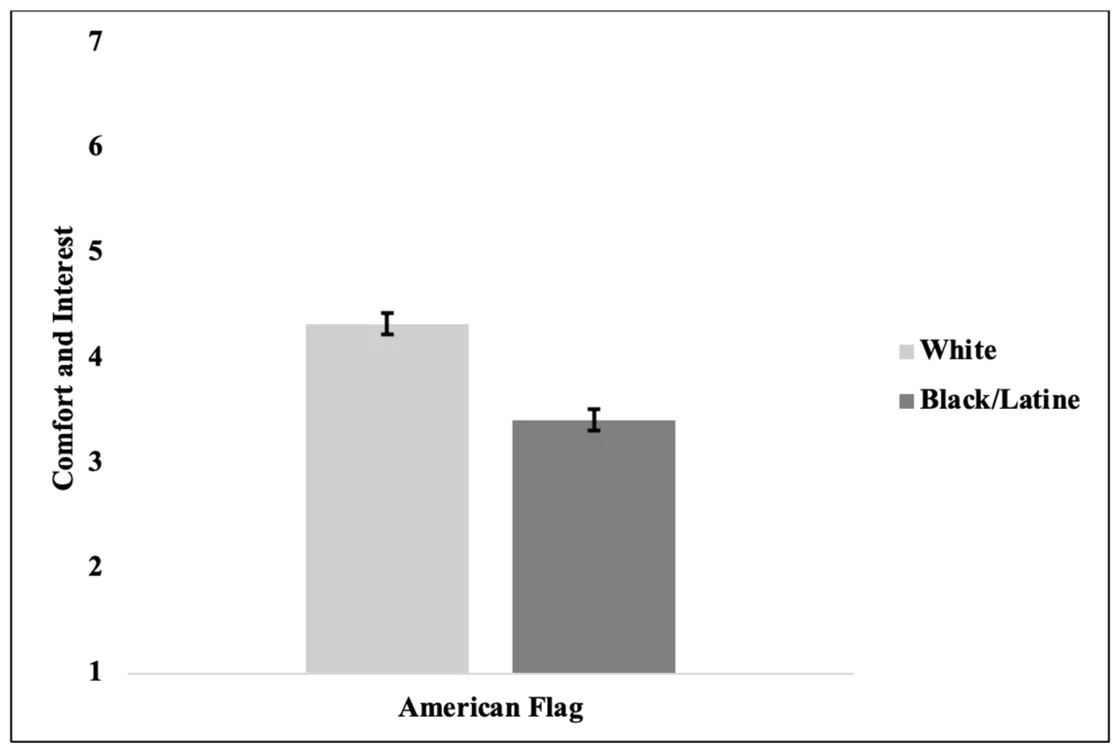
Study 2 participant race by flag condition interaction for Comfort and Interest. **Note:** Error bars show standard errors.

**Figure 4:**
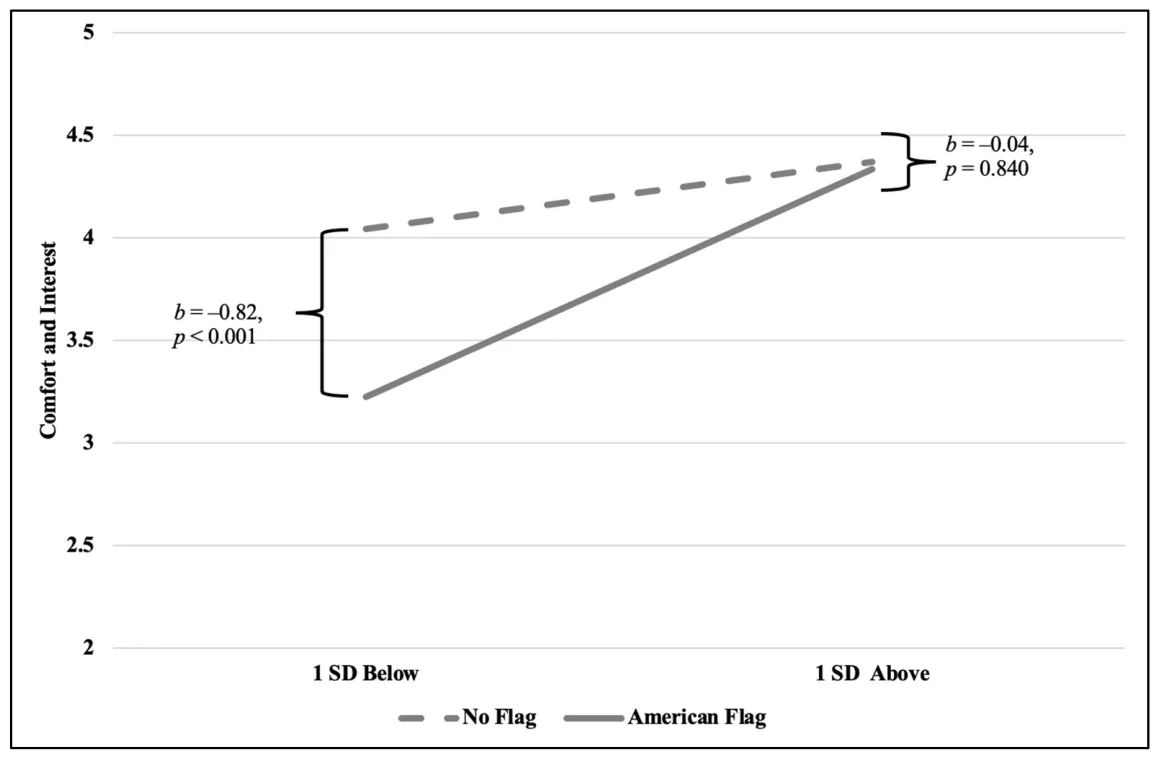
Study 2 simple effects of flag condition (no flag vs. American flag) and participant SDO on predicting Comfort and Interest at 1 standard deviation below and above the mean of the participant SDO scale.

**Figure 5:**
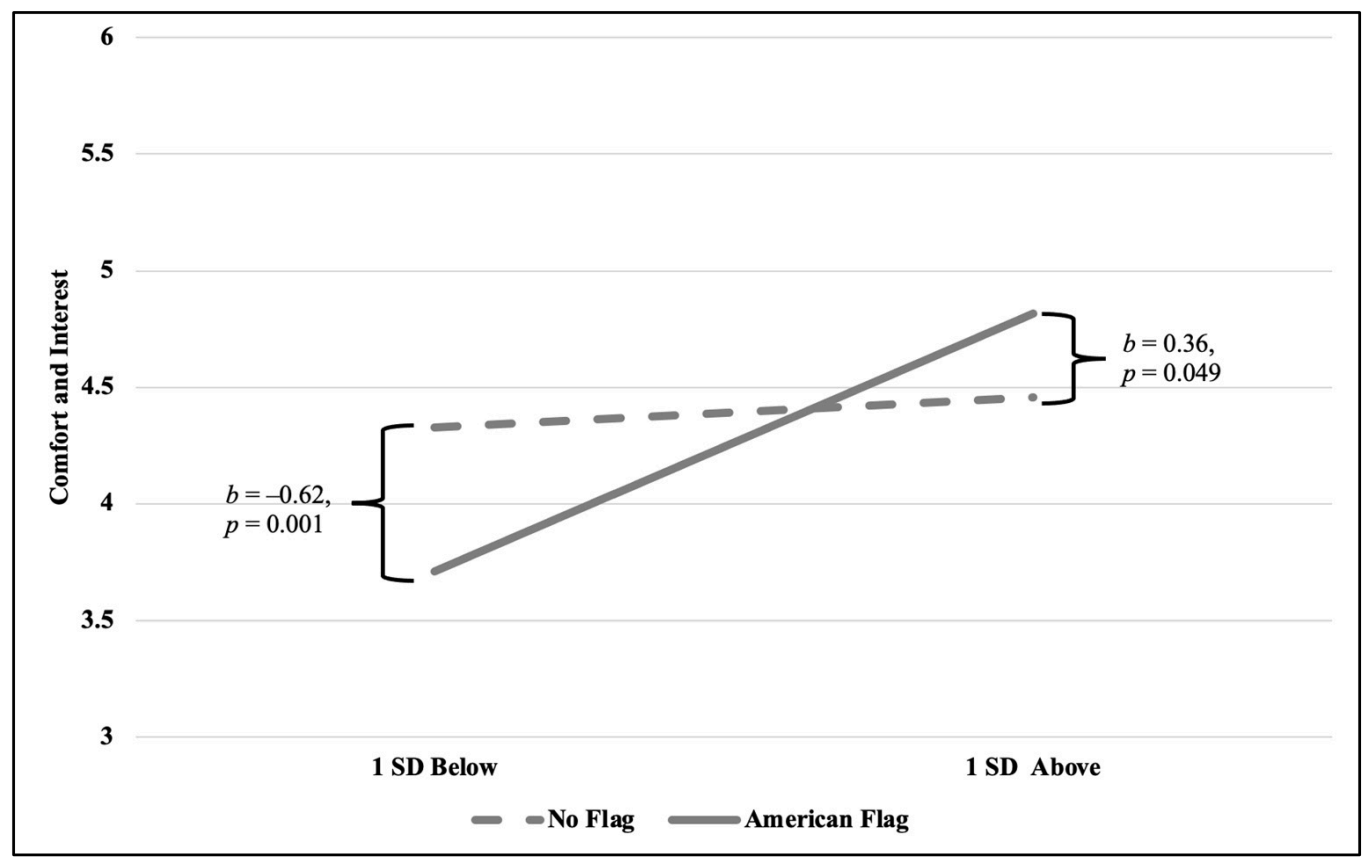
Study 3 simple effects of flag condition and Participant SDO on predicting Comfort and Interest at 1 standard deviation below and above the mean of the Participant SDO scale.

**Table 1:** Measure information and statistics across three experiments.

Measures	Example Item	Scale End Points	# of Items
Comfort and Interest	“I think I would feel comfortable interacting with Dr. Johnson.”	1—*Not at all agree* to 7—*Strongly agree*	3
Perceived Racism	“How likely is it that this person has racist beliefs?”	1—*Very slightly or not at all* to 5—*Extremely or a lot*	4
Perceived Sexism	“How likely is it that this person has sexist beliefs?”	1—*Very slightly or not at all* to 5—*Extremely or a lot*	4
Professor SDO	“It is unjust to try to make groups equal”	1—*Dr. Johnson would strongly oppose* to 7—*Dr. Johnson would strongly favor*	8
Professor Selection	“Would you want Dr. Johnson as your own professor?”	1—*Definitely not* to 7—*Yes*	1
Participant SDO	“We should do what we can to equalize conditions for different groups”	1—*Strongly disagree* to 7—*Strongly agree*	4
Political Orientation	“How would you describe your political orientation?”	1—*Extremely liberal* to 7—*Extremely conservative*	1

**Note:** For Study 3, participants examined a Zoom background of a coworker. Thus, measure items were framed towards their coworker, Alex Johnson.

**Table 2:** Measure information and statistics, reliabilities, and correlations for Study 1.

Measure	M (SD)	1	2	3	4	5	6	7
1. Comfort and Interest	4.13 (1.40)	(0.91)						
2. Perceived Racism	2.55 (0.88)	−0.53 ***	(0.79)					
3. Perceived Sexism	2.68 (0.89)	−0.50 ***	0.72 ***	(0.84)				
4. Professor SDO	3.71 (1.04)	−0.48 ***	0.61 ***	0.59 ***	(0.85)			
5. Professor Selection	4.00 (1.44)	0.64 ***	−0.54 ***	−0.51 ***	−0.47 ***	(NA)		
6. Participant SDO	1.88 (0.87)	0.06	−0.05	−0.06	0.13 **	0.04	(0.75)	
7. Political Orientation	3.11 (1.30)	0.28 ***	−0.23 ***	−0.22 ***	−0.05	0.24 ***	0.38 ***	(NA)

**Note:** The reliability for each scale is shown on the diagonal. ** *p* < 0.01, *** *p* < 0.001.

**Table 3:** Study 1 means, standard deviation, one-way ANOVA results across conditions.

**Means, Standard Deviation, One-Way ANOVA Results Across Conditions**									
**Measures**	**No Flag**		**Denver Broncos Flag**		**American Flag**		***F* (556)**	***p***	***η_p_* ^2^**
	**M**	**SD**	**M**	**SD**	**M**	**SD**			
Comfort and Interest	3.94	1.29	4.59	1.26	3.85	1.52	16.31	<0.001	0.055
Perceived Racism	2.35	0.76	2.21	0.71	3.07	0.89	64.78	<0.001	0.189
* Perceived Sexism	2.49	0.83	2.48	0.77	3.08	0.93	31.47	<0.001	0.102
* Professor SDO	3.45	0.91	3.35	0.96	4.30	0.97	57.11	<0.001	0.170
* Professor Selection	3.77	1.35	4.71	1.27	3.55	1.44	38.67	<0.001	0.122
**Differences Between Flag Conditions**									
**Measures**	**No Flag vs. Denver Broncos**			**No Flag vs. American**			**Denver Broncos vs. American**		
	**MD (SE)**	***p***	***d***	**MD (SE)**	***p***	***d***	**MD (SE)**	***p***	***d***
Comfort and Interest	0.65 (0.14)	<0.001	0.51	0.09 (0.14)	1.00	0.06	0.74 (0.14)	<0.001	0.53
Perceived Racism	0.15 (0.08)	0.220	0.20	0.72 (0.08)	<0.001	0.87	0.87 (0.08)	<0.001	1.08
Perceived Sexism	0.01 (0.09)	1.00	0.01	0.59 (0.09)	<0.001	0.68	0.60 (0.09)	<0.001	0.71
Professor SDO	0.10 (0.10)	0.887	0.11	0.85 (0.10)	<0.001	0.90	0.95 (0.10)	<0.001	0.98
Professor Selection	0.94 (0.14)	<0.001	0.72	0.22 (0.14)	0.347	0.16	1.16 (0.14)	<0.001	0.86

**Note:** MD = Mean Difference; SE = Standard Error; * df = 557.

**Table 4:** Interactions with Participant SDO and Flag Conditions from Study 1.

**Measures**	**Flag Condition** **(No Flag vs. Denver Broncos Flag) × Participant SDO**			**Flag Condition** **(No Flag vs. American Flag) × Participant SDO**		
	***b* (SE)**	***t* (553)**	***p***	***b* (SE)**	***t* (553)**	***p***
Comfort and Interest	0.13 (0.16)	0.84	0.400	0.65 (0.16)	4.09	<0.001
Perceived Racism	−0.13 (0.09)	−1.45	0.147	−0.37 (0.09)	−3.92	<0.001
* Perceived Sexism	−0.16 (0.10)	−1.67	0.095	−0.35 (0.10)	−3.58	<0.001
* Professor SDO	0.02 (0.11)	0.19	0.852	−0.36 (0.11)	−3.30	0.001
* Professor Selection	0.15 (0.16)	0.97	0.333	0.52 (0.16)	3.24	0.001
**Simple Effects of Flag Condition (No Flag vs. American Flag) at 1 Standard Deviation Above and Below the Mean on the Participant SDO Scale**						
**Measures**	**1 Standard Deviation Below**			**1 Standard Deviation Above**		
	***b* (SE)**	***t* (553)**	***p***	***b* (SE)**	***t* (553)**	***p***
Comfort and Interest	−0.63 (0.20)	−3.23	0.001	0.50 (0.20)	2.54	0.011
Perceived Racism	1.03 (0.11)	9.02	<0.001	0.40 (0.11)	3.51	<0.001
Perceived Sexism	0.89 (0.12)	7.34	<0.001	0.28 (0.12)	2.30	0.022
Professor SDO	1.18 (0.14)	8.72	<0.001	0.55 (0.13)	4.08	<0.001
Professor Selection	−0.66 (0.20)	−3.35	0.001	0.24 (0.20)	1.21	0.226

**Note:** *b* = unstandardized regression coefficient; SE = Standard Error; * df = 554.

**Table 5:** Measure information and statistics, reliabilities, and correlations for Study 2.

Measure	M (SD)	1	2	3	4	5	6	7
1. Comfort and Interest	4.58 (1.66)	(0.94)						
2. Perceived Racism	2.24 (1.00)	−0.72 ***	(0.87)					
3. Perceived Sexism	2.24 (0.98)	−0.69 ***	0.83 ***	(0.90)				
4. Professor SDO	3.10 (1.46)	−0.65 ***	0.73 ***	0.68 ***	(0.93)			
5. Professor Selection	4.40 (1.67)	0.83 ***	−0.67 ***	−0.66 ***	−0.64 ***	(NA)		
6. Participant SDO	1.85 (1.04)	0.01	0.04	0.03	0.20 ***	−0.04	(0.87)	
7. Political Orientation	2.89 (1.55)	0.11 **	−0.12 **	−0.12 **	−0.02	0.08	0.58 ***	(NA)

**Note:** The reliability for each scale is shown on the diagonal. ** *p* < 0.01, *** *p* < 0.001.

**Table 6:** Study 2 means and standard deviation across conditions.

Means and Standard Deviation Across Conditions														
**Measures**	**No Flag**				**Inclusive Flag**					**American Flag**				
	**White** **(*n* = 84)**		**Black/Latine** **(*n* = 92)**		**White** **(*n* = 83)**			**Black/Latine** **(*n* = 94)**		**White** **(*n* = 83)**		**Black/Latine** **(*n* = 106)**		
	**M**	**SD**	**M**	**SD**	**M**	**SD**		**M**	**SD**	**M**	**SD**	**M**		**SD**
Comfort and Interest	4.27	1.39	4.13	1.37	5.87	1.33		5.73	1.12	4.33	1.62	3.41		1.50
Perceived Racism	2.21	0.70	2.27	0.83	1.53	0.75		1.47	0.65	2.73	0.91	3.07		0.95
Perceived Sexism	2.31	0.84	2.27	0.86	1.58	0.74		1.56	0.70	2.77	1.00	2.88		0.85
Professor SDO	3.22	0.94	3.39	0.99	1.57	0.67		1.98	0.96	4.14	1.26	4.15		1.41
Professor Selection	3.99	1.31	4.07	1.31	5.60	1.51		5.52	1.42	3.90	1.64	3.49		1.50
**Measures**	**No Flag**		**Inclusive Flag**		**American Flag**				**White**		**Black/Latine**			
	**M**	**SD**	**M**	**SD**	**M**		**SD**		**M**	**SD**	**M**		**SD**	
Comfort and Interest	4.19	1.38	5.80	1.22	3.81		1.61		4.82	1.63	4.38		1.66	
Perceived Racism	2.24	0.77	1.50	0.70	2.92		0.94		2.16	0.93	2.30		1.05	
Perceived Sexism	2.29	0.85	1.57	0.72	2.83		0.92		2.22	0.99	2.26		0.97	
Professor SDO	3.31	0.97	1.79	0.86	4.14		1.34		2.98	1.45	3.21		1.46	
Professor Selection	4.03	1.31	5.56	1.46	3.67		1.57		4.50	1.68	4.33		1.65	

**Table 7:** Study 2 between-subjects results for flag condition.

**Measures**	**Flag Condition**			**Participant Race Condition**						**Flag × Participant Race**		
	***F* (536)**	***p***	***η_p_* ^2^**	***F* (536)**	***p***	***η_p_* ^2^**	***MD (SE)***		***d***	***F* (536)**	***p***	***η_p_* ^2^**
Comfort and Interest	98.17	<0.001	0.268	10.79	0.001	0.020	0.40 (0.12)		0.26	4.72	0.009	0.017
Perceived Racism	136.46	<0.001	0.337	2.61	0.107	0.005	0.11 (0.07)		−0.15	2.85	0.059	0.011
Perceived Sexism	102.95	<0.001	0.278	0.06	0.813	<0.001	0.02 (0.07)		−0.04	0.51	0.603	0.002
Professor SDO	222.15	<0.001	0.453	4.30	0.038	0.008	0.19 (0.09)		−0.16	1.51	0.221	0.006
Professor Selection	83.92	<0.001	0.238	1.23	0.268	0.002	0.14 (0.13)		0.10	1.35	0.261	0.005
**Differences Between Flag Conditions**												
**Measures**	**No Flag vs. Inclusive Flag**			**No Flag vs. American**						**Inclusive Flag vs. American**		
	**MD (SE)**	***p***	***d***	**MD (SE)**		***p***		***d***		**MD (SE)**	***p***	***d***
Comfort and Interest	1.60 (0.15)	<0.001	1.23	0.33 (0.15)		0.078		0.25		1.93 (0.15)	<0.001	1.39
Perceived Racism	0.74 (0.09)	<0.001	1.01	0.67 (0.09)		<0.001		0.80		1.40 (0.09)	<0.001	1.72
Perceived Sexism	0.72 (0.09)	<0.001	0.92	0.53 (0.09)		<0.001		0.61		1.26 (0.09)	<0.001	1.53
Professor SDO	1.53 (0.12)	<0.001	1.66	0.84 (0.11)		<0.001		0.71		2.37 (0.11)	<0.001	2.09
Professor Selection	1.54 (0.16)	<0.001	1.10	0.33 (0.15)		0.095		0.25		1.87 (0.15)	<0.001	1.24

**Note:** MD = Mean Difference; SE = Standard Error.

**Table 8:** Interactions with participant SDO and flag conditions from Study 2.

**Measures**	**Flag Condition** **(No Flag vs. Inclusive Flag) × Participant SDO**			**Flag Condition** **(No Flag vs. American Flag) × Participant SDO**		
	***b* (SE)**	***t* (536)**	***p***	***b* (SE)**	***t* (536)**	***p***
Comfort and Interest	−0.89 (0.15)	−6.10	<0.001	0.41 (0.13)	3.17	0.002
Perceived Racism	0.47 (0.09)	5.39	<0.001	−0.07 (0.08)	−0.93	0.352
Perceived Sexism	0.45 (0.09)	5.02	<0.001	−0.07 (0.08)	−0.81	0.419
Professor SDO	0.47 (0.12)	4.02	<0.001	−0.21 (0.10)	−2.02	0.044
Professor Selection	−1.04 (0.15)	−7.01	<0.001	0.34 (0.13)	2.54	0.012
**Simple Effects of Flag Condition (No Flag vs. Inclusive Flag) at** **1 Standard Deviation Above and Below the Mean on the Participant SDO Scale**						
**Measures**	**1 Standard Deviation Below**			**1 Standard Deviation Above**		
	***b* (SE)**	***t* (536)**	***p***	***b* (SE)**	***t* (536)**	***p***
Comfort and Interest	2.24 (0.18)	12.53	<0.001	0.56 (0.21)	2.62	0.009
Perceived Racism	−1.08 (0.11)	−10.14	<0.001	−0.20 (0.13)	−1.54	0.124
Perceived Sexism	−1.05 (0.11)	−9.56	<0.001	−0.20 (0.13)	−1.53	0.126
Professor SDO	−1.81 (0.14)	−12.79	<0.001	−0.94 (0.17)	−5.50	<0.001
Professor Selection	2.27 (0.18)	12.43	<0.001	0.30 (0.22)	1.38	0.168
**Simple Effects of Flag Condition (No Flag vs. American Flag) at** **1 Standard Deviation Above and Below the Mean on the Participant SDO Scale**						
**Measures**	**1 Standard Deviation Below**			**1 Standard Deviation Above**		
	***b* (SE)**	***t* (536)**	***p***	***b* (SE)**	***t* (536)**	***p***
Comfort and Interest	−0.82 (0.18)	−4.50	<0.001	−0.04 (0.19)	−0.20	0.840
Professor SDO	1.03 (0.14)	7.11	<0.001	0.63 (0.15)	4.26	<0.001
Professor Selection	−0.72 (0.19)	−3.86	<0.001	−0.08 (0.19)	−0.42	0.678

**Table 9:** Measure information and statistics, reliabilities, and correlations for Study 3.

Measure	M (SD)	1	2	3	4	5	6
1. Comfort and Interest	4.29 (1.56)	(0.92)					
2. Perceived Racism	2.78 (0.94)	−0.61 ***	(0.82)				
3. Perceived Sexism	2.71 (0.93)	−0.60 ***	0.76 ***	(0.86)			
4. Coworker SDO	3.85 (1.30)	−0.55 ***	0.65 ***	0.62 ***	(0.89)		
5. Participant SDO	2.07 (1.15)	0.21 ***	−0.13 **	−0.12 **	−0.02	(0.80)	
6. Political Orientation	3.40 (1.69)	0.31 ***	−0.21 ***	−0.25 ***	−0.21 ***	0.39 ***	(NA)

**Note:** The reliability for each scale is shown on the diagonal. ** *p* < 0.01, *** *p* < 0.001.

**Table 10:** Study 3 means and standard deviation across conditions.

Means and Standard Deviation Across Conditions								
**Measures**	**White**				**Black**			
	**No Flag** **(*n* = 137)**		**American Flag** **(*n* = 140)**		**No Flag** **(*n* = 132)**		**American Flag** **(*n* = 147)**	
	***M***	***SD***	***M***	***SD***	***M***	***SD***	***M***	***SD***
Comfort and Interest	4.28	1.25	4.28	1.82	4.52	1.47	4.11	1.64
Perceived Racism	2.39	0.76	3.03	1.08	2.55	0.79	3.13	0.87
Perceived Sexism	2.49	0.80	2.90	1.08	2.53	0.84	2.89	0.90
Coworker SDO	3.54	0.92	4.28	1.46	3.39	1.15	4.15	1.38
**Measures**	**No Flag**		**American Flag**		**White**		**Black**	
	***M***	***SD***	***M***	***SD***	***M***	***SD***	***M***	***SD***
Comfort and Interest	4.40	1.36	4.19	1.73	4.28	1.56	4.30	1.57
Perceived Racism	2.47	0.77	3.08	0.98	2.71	0.99	2.85	0.88
Perceived Sexism	2.51	0.82	2.90	0.99	2.70	0.97	2.72	0.89
Coworker SDO	3.47	1.04	4.21	1.42	3.92	1.27	3.79	1.33

**Table 11:** Study 3 between-subjects ANOVA results.

	ANOVA Results												
Measures	Participant Race Condition					Flag Condition					Participant Race × Flag Condition		
	*F* (1,552)	*p*	*η_p_* ^2^	*MD* (*SE*)	*d*	*F* (1,552)	*p*	*η_p_* ^2^	*MD* (*SE*)	*d*	*F* (1,552)	*p*	*η_p_* ^2^
Comfort and Interest	0.06	0.812	0.000	0.03 (0.13)	−0.01	2.39	0.123	0.004	0.21 (0.13)	0.13	2.31	0.129	0.004
Perceived Racism	2.92	0.088	0.005	0.13 (0.08)	−0.15	65.93	<0.001	0.107	0.61 (0.08)	−0.69	0.21	0.647	0.000
Perceived Sexism	0.05	0.816	0.000	0.02 (0.08)	−0.02	25.38	<0.001	0.044	0.39 (0.08)	−0.43	0.14	0.710	0.000
Coworker SDO	1.87	0.172	0.003	0.15 (0.11)	0.10	49.63	<0.001	0.082	0.75 (0.11)	−0.60	0.02	0.899	0.000

**Note:** MD = Mean Difference; SE = Standard Error.

**Table 12:** Interactions with participant SDO and flag condition from Study 3.

**Testing for 2-Way Interactions with Flag Condition and Participant SDO**							
**Measures**	**Flag × SDO**						
	***b* (SE)**		***t* (552)**		***p***		
Comfort and Interest	0.44 (0.11)		3.93		<0.001		
Perceived Racism	−0.35 (0.06)		−5.52		<0.001		
Perceived Sexism	−0.32 (0.07)		−4.94		<0.001		
Coworker SDO	−0.36 (0.09)		−3.89		<0.001		
**Simple Effects of Flag Condition at 1 Standard Deviation Above and Below the Mean on the Participant SDO Scale**							
**Measures**	**1 Standard Deviation Below**			**1 Standard Deviation Above**			
	***b* (SE)**	***t* (552)**	***p***	***b* (SE)**	***t* (552)**	***p***	
Comfort and Interest	−0.62 (0.18)	−3.51	0.001	0.36 (0.18)	1.97	0.049	
Perceived Racism	0.97 (0.10)	9.72	<0.001	0.19 (0.10)	1.86	0.064	
Perceived Sexism	0.72 (0.10)	6.98	<0.001	−0.00 (0.11)	0.00	1.00	
Coworker SDO	1.13 (0.14)	7.84	<0.001	0.34 (0.15)	2.27	0.024	

**Table 13:** Internal mega-analysis.

**Measures**	**Flag Condition**				**Participant Race Condition**			**Flag Condition × Participant Race**		
	***b* (SE)**	***t* (1094)**		***p***	***b* (SE)**	***t* (1093)**	***p***	***b* (SE)**	***t* (1093)**	***p***
Comfort and Interest	−0.66 (0.10)	−6.77		<0.001	−0.22 (0.10)	−2.29	0.022	−0.53 (0.19)	−2.78	0.005
Perceived Racism	0.81 (0.05)	14.99		<0.001	0.13 (0.05)	1.09	2.43	0.14 (0.11)	1.31	0.191
Perceived Sexism	0.63 (0.06)	11.26		<0.001	0.02 (0.06)	0.39	0.698	0.06 (0.11)	0.57	0.570
Target SDO	1.15 (0.08)	14.69		<0.001	0.01 (0.08)	0.13	0.900	−0.13 (0.15)	−0.81	0.417
** Simple Effects**										
**Measures**	**Simple Effects of Participant Race in the** **Comparison Flags Condition**					**Simple Effects of Participant Race in** **American Flag Condition**				
	***b* (SE)**		***t* (1093)**		***p***	***b* (SE)**		***t* (1093)**	***p***	
Comfort and Interest	−0.05 (0.13)		−0.38		0.706	0.49 (0.14)		3.37	<0.001	
Perceived Racism	−0.06 (0.07)		−0.86		0.393	−0.20 (0.08)		−2.48	0.013	
Perceived Sexism	0.01 (0.07)		0.14		0.891	−0.05 (0.08)		−0.67	0.525	
Target SDO	−0.07 (0.10)		−0.71		0.476	0.05 (0.12)		0.46	0.649	

**Note:** Comfort and Interest degrees of freedom for flag condition = 1046 and participant race = 1094.

## Data Availability

The current research was pre-registered on the Open Science Framework (OSF) at https://osf.io/qwxc6/overview?view_only=ef8c686f16d6485bbd3bf749b4abffee (accessed on 4 April 2024) (Study 2) and https://osf.io/utkxc/overview?view_only=34d2bd5b2e614e3082885e001ad82959 (accessed on 1 July 2024) (Study 3). The data and materials supporting the findings of this manuscript are available on OSF at https://osf.io/n2a85/overview?view_only=7cf6ae79ec31441bbe14d39b56ef6fa7 (accessed on 13 August 2026) (Studies 1 and 2) and https://osf.io/rfzh4/overview?view_only=a28957d8f7404947a7e5c4f7e787875f (accessed on 13 August 2026) (Study 3).

## Notes

1 We asked participants their perceptions of Dr. Johnson’s age, race, and gender (Studies 1 and 2). The majority of participants perceived Dr. Johnson as a White man between the ages of 51 and 55. These findings are reported in the Supplementary Materials.

2 Across all studies, we excluded participants who failed the flag attention check because we wanted to examine how people who are conscious of the flag’s presence perceived a person (i.e., professor or coworker) who displayed it. Among participants who failed the flag attention check (Study 1: *n* = 9; Study 2: *n* = 6; Study 3: *n* = 8), only one participant in all three studies failed the flag attention check in the American flag condition.

3 Across all studies, we also measured perceived likability and positive and negative affect. In Studies 1 and 2, we additionally measured perceived similarity, perceived prejudice, and race- and gender-based classroom mistreatment. Results for these measures are reported in the Supplementary Materials.

4 In Study 1, we found that the flag condition did not impact participant SDO (*p* = 0.293). In Study 2, we found a main effect of the flag condition (*p* = 0.008), such that participants reported higher SDO in the American flag versus inclusive flag condition (*p* = 0.006). However, this effect was not significant for the no flag versus inclusive flag and American flag contrasts (*ps* > 0.292). In Study 3, participants reported higher SDO in the no flag versus American flag condition (*p* = 0.032).

5 Our comparisons focus on the presence, direction, and pattern of moderation effects rather than their absolute magnitude.

6 We also examined whether the strength of the flag effect differed between White and Black/Latine participants. These results are reported in the Supplementary Materials.

7 A post hoc sensitivity analysis conducted using G*Power (Faul et al., 2007) determined we were adequately powered to find small effect sizes (*f* = 0.13) at 80% power and *p* < 0.05. Although, we had a large sample size, the study design including the timepoint condition was a 3 (timepoint) × 2 (participant race) × 2 (flag condition) between-subjects design; thus, we had 12 conditions and may have been underpowered to find the effect.

8 We asked participants their perceptions of the coworker Alex Johnson’s age, race, and gender. The majority of participants perceived Alex Johnson as a White man between the ages of 31 and 35. These findings are reported in the Supplementary Materials.

9 We also measured participants’ general perceptions of the American flag, including whether they perceive it as threatening. These results are reported in the Supplementary Materials.

## Supplementary Materials

The following supporting information can be downloaded at: https://www.mdpi.com/article/10.3390/bs16101776/s1, Table S1: Attention Check Items and Exclusions Across Studies; Table S2: Demographic Information Across Initial Supplemental Study, Study 1, Study 2, and Study 3; Table S3: Perceived Target Demographic Information Across Studies; Table S4; Supplemental Measure Information and Statistics, Reliabilities, and Correlations for Study 1 (N. J. Goldstein et al., 2014; Watson et al., 1988); Table S5: Study 1 Supplemental Means, Standard Deviations, and One-way ANOVA Results Across Conditions; Table S6: Study 1 Means and Standard Deviations for Participant Gender and Flag Condition; Table S7: Study 1 Between-Subjects Results for Participant Gender and Flag Condition; Table S8: Study 1 Means and Standard Deviations for Differences in Perceptions of Professor Race and Flag Condition; Table S9: Study 1 Between-Subjects Results for Differences in Perceptions of Professor Race and Flag Condition; Table S10: Study 1 Means and Standard Deviations for Differences in Perceptions of Professor Gender and Flag Condition; Table S11: Study 1 Between-Subjects Results for Differences in Perceptions of Professor Gender and Flag Condition; Table S12: Interactions with Political Orientation and Flag Conditions from Study 1; Table S13: Participant Race by Flag Condition Interactions for Study 2; Table S14: Interactions with Political Orientation, Participant Race (Black/Latine vs. White), and Flag Conditions from Study 2; Table S15: Simple Effects of Flag Condition at 1 Standard Deviation Below and Above the Mean on the Political Orientation Scale from Study 2; Table S16: Interactions with Participant SDO, Participant Race (Black/Latine vs. White), and Flag Conditions from Study 2; Figure S1: Simple Effects of Flag Condition for White and Black/Latine Participants at 1 Standard Deviation Below (left panel) and Above (right panel) the Mean on the Participant SDO Scale Predicting Likability; Figure S2: Simple Effects of Flag Condition for White and Black/Latine Participants at 1 Standard Deviation Below (left panel) and Above (right panel) the Mean on the Participant SDO Scale Predicting Comfort and Interest; Figure S3: Simple Effects of Flag Condition for White and Black/Latine Participants at 1 Standard Deviation Below (left panel) and Above (right panel) the Mean on the Participant SDO Scale Predicting Professor Selection; Table S17: Study 2 Means and Standard Deviations for Participant Gender and Flag Condition; Table S18: Study 2 Between-Subjects Results for Participant Gender and Flag Condition; Table S19: Study 2 Means and Standard Deviations for Differences in Perceptions of Professor Race and Flag Condition; Table S20: Study 2 Between-Subjects Results for Differences in Perceptions of Professor Race and Flag Condition; Table S21: Study 2 Means and Standard Deviations for Differences in Perceptions of Professor Gender and Flag Condition; Table S22: Study 2 Between-Subjects Results for Differences in Perceptions of Professor Gender and Flag Condition; Table S23: Supplemental Measure Information and Statistics, Reliabilities, and Correlations for Study 2; Table S24: Supplemental Study 2 Means and Standard Deviation for Black vs. Latine Participants; Table S25: Supplemental Study 2 Between-Subjects ANOVA Results for Black vs. Latine participants; Table S26: Interactions with Participant SDO, Participant Race (Black vs. Latine), and Flag Conditions from Supplemental Study 2; Table S27: Simple Effects of Flag Condition at 1 Standard Deviation Below and Above the Mean on the Participant SDO Scale from Supplemental Study 2; Table S28: Interactions with Political Orientation, Participant Race (Black vs. Latine), and Flag Conditions from Supplemental Study 2; Table S29: Simple Effects of Flag Condition at 1 Standard Deviation Below and Above the Mean on the Political Orientation Scale from Supplemental Study 2; Table S30: Supplemental Measure Information and Statistics, Reliabilities, and Correlations for Study 3; Table S31: Study 3 Supplemental Means and Standard Deviation Across Conditions; Table S32: Study 3 Supplemental Between-Subjects ANOVA Results for Timepoint, Flag, and Participant Race Conditions; Table S33: Study 3 Supplemental Interactions with Participant SDO, Timepoint, Participant Race, and Flag Condition; Table S34: Study 3 Supplemental Interactions with Political Orientation, Timepoint, Participant Race, and Flag Condition; Table S35: Study 3 Means and Standard Deviations for Participants Gender and Flag Conditions; Table S36: Study 3 Between-Subjects ANOVA Results for Participants Gender and Flag Conditions; Table S37: Study 3 Means and Standard Deviations for Perceptions of Coworker Race and Flag Conditions; Table S38: Study 3 Between-Subjects ANOVA Results for Perceptions of Coworker Race and Flag Conditions; Table S39: Study 3 Means and Standard Deviations for Perceptions of Coworker Gender and Flag Conditions; Table S40: Study 3 Between-Subjects ANOVA Results for Perceptions of Coworker Gender and Flag Conditions; Table S41: Measure Information and Statistics, Reliabilities, and Correlations for the Initial Supplemental Study; Table S42: Initial Supplemental Study Means, Standard Deviations, T-test Results Across Conditions.
