# Supplementary material for: Stars, Stripes, and Perceptions: Examining the Impact of the American Flag on Anticipated Interactions
Source: Behav Sci (Basel). 2026 Sep 28;16(10):1776. doi: 10.3390/bs16101776 (PMC13701950; doi:10.3390/bs16101776)
Supplement: Supplementary file 1 [file behavsci-16-01776-s001.zip › behavsci-4352261-supplementary.pdf]

Stars, Stripes, and Perceptions: Examining the Impact of the American Flag on Anticipated Interactions: Supplemental Materials

Table of Contents

Table of Contents 1
Additional Information about Participant Exclusions Across Studies 2
Attention Items Check Wording 2
Additional Measures, Analyses, and Results for Study 1 6
Additional Measures, Analyses, and Results for Study 2 21
Additional Measures, Analyses, and Results for Study 3 51
Initial Supplemental Study 66

Supplemental Materials

Additional Information about Participant Exclusions Across Studies

For the Initial Supplemental Study, we excluded 1 participant for failing attention checks. In Study 1, we excluded 15 participants for failing attention checks. For Study 2, we excluded 21 participants for not identifying as Black, Latine, or White, 29 participants for not identifying as current students, and 6 participants for failing attention checks. Finally for Study 3, we excluded 35 participants for not identifying as Black or White, and 8 participants for failing attention checks. In both Studies 1 and 2, participants who failed attention checks did not vary systematically across conditions (see Table S1). In Study 3, significantly more participants in the no flag ($N = 7$) versus American flag ($N = 1$) failed the flag manipulation check (see Table S1).

Table S1

Attention Check Items and Exclusions Across Studies

	Initial Supplemental Study		Study 1		Study 2		Study 3	
Attention check	Participants excluded	Chi-square analyses for exclusion across groups	Participants excluded	Chi-square analyses for exclusion across groups	Participants excluded	Chi-square analyses for exclusion across groups	Participants excluded	Chi-square analyses for exclusion across groups
Flag Manipulation Check	0 (0.0%)	N/A	9 (1.6%)	χ^2 (2, N = 576) = 2.65, p = .265	6 (1.0%)	χ^2 (2, N = 577) = 3.09, p = .214	8 (1.4%)	χ^2 (1, N = 564) = 4.83, p = .028
Open-ended Attention Check	1 (0.4%)	χ^2 (1, N = 254) = 1.00, p = .316	6 (1.1%)	χ^2 (2, N = 567) = 1.03, p = .598	0 (0.0%)	N/A	0 (0.0%)	N/A

Attention Items Check Wording

Open-ended question

- Wording for the Initial Supplemental Study, 1, 2:
    - *Briefly describe your impression of Dr. Johnson (the person whose office is pictured in the zoom background picture). For example, what stood out to you about Dr. Johnson?*
- Wording for Study 3:
    - *Briefly describe your impression of Alex Johnson (the person whose office is pictured in the zoom background picture). For example, what stood out to you about Alex Johnson?*

Flag Attention Check

- Wording for the Initial Supplemental Study, Study 1, and Study 2:

- ○ *What kind of flag was in Dr. Johnson's office in the Zoom background picture?*
- Wording for Study 3:
  - ○ *What kind of flag was in Alex Johnson's office in the Zoom background picture?*

Table S2

Demographic Information Across Studies Initial Supplemental Study, Study 1, Study 2, and Study 3

	Race	School Level	School Type	First Gen Student	Year in College	Employment	Education	Country of Origin	Income	Political Orientation	Age
Initial Study	190 (75.1%) White, 11 (4.3%) Black, 11 (4.3%) Asian, 17 (6.7%) Latino, 5 (2.0%) Middle Eastern, 1 (0.4%) South Asian, 15 (5.9%) Multiple Racial	N/A	N/A	N/A	N/A	N/A	N/A	232 (91.7%) USA	31 (12.3%) under $25k, 13 (5.1%) $25-49K, 28 (11.1%) $50-74K, 32 (12.6%) $75-99K, 45 (17.9%) $100-149K, 102 (40.3%) over $150K	N/A	18-24 (*M* = 19.19, *SD* = 1.22)
Study 1	412 (73.6%) White, 11 (2.0%) Black, 22 (3.9%) Asian, 52 (9.3%) Latino, 8 (1.4%) Middle Eastern, 11 (2.0%) South Asian, 42 (7.5%) Multiple Racial	N/A	N/A	N/A	N/A	N/A	N/A	509 (90.9%) USA	60 (10.7%) under $25k, 35 (6.3%) $25k-49k, 57 (10.2%) $50-74K, 71 (12.7%) $75-99K, 121 (21.6%) $100-149K, 213 (38.0%) over $150K	35 (6.3%) identified as extremely liberal, 199 (35.5%) identified as mostly liberal, 107 (19.1%) identified as somewhat liberal, 132 (23.6%) identified neither liberal nor conservative, 62 (11.1%) identified as somewhat conservative, 23	18-59 (*M* = 18.92, *SD* = 2.12)

										(4.1%) identified as mostly conservative, 2 (0.4%) identified as extremely conservative	
Study 2	250 (46.1%) White, 292 (53.9%) Black/Latine	17 (3.1%) High School/GED, 109 (20.1%) 2 year college, 326 (60.1%) 4 year college, 69 (12.7%) Graduate School, 16 (3.0%) Professional	260 (48.0%) Predominately White Institution, 22 (4.1%) Historically Black College, 260 (48.0%) Neither	154 (28.4%) Yes, 114 (21.0%) No	55 (10.1%) are 1st year, 105 (19.4%) are 2nd year, 115 (21.2%) are 3rd year, 130 (24.0%) are 4th year, 30 (5.5%) are 5th year or above	N/A	N/A	531 (98.0%) USA	137 (25.3%) under $25k, 134 (24.7%) $25k-49k, 88 (16.2%) $50-74K, 68 (12.5%) $75-99K, 76 (14.0%) $100-149K, 39 (7.2%) over $150K	113 (20.8%) identified as extremely liberal, 156 (28.8%) identified as mostly liberal, 85 (15.7%) identified as somewhat liberal, 100 (18.5%) identified neither liberal nor conservative, 51 (9.4%) identified as somewhat conservative, 28 (5.2%) identified as mostly conservative, 9 (1.7%) identified as extremely conservative	18-27 (*M* =22.01, *SD* = 1.95)
Study 3	277 (49.8%) White, 279 (50.2%) Black	N/A	N/A	N/A	N/A	241 (43.3%) Full-Time Outside Home, 52 (9.4%) Part-Time Outside Home, 70 (12.6%) Full-Time at Home, 37 (6.7%) Part-Time at Home, 18 (3.2%) Stay-at-Home Parent, 39 (7.0%) Part-Time at Home and Part-Time Outside the Home, 51 (9.2%) Not employed but	4 (0.7%) Less than High School, 161 (29.0%) High School/GED, 77 (13.8%) 2-year College Degree, 206 (37.1%) 4-year College Degree, 94 (16.9%) Master's degree, 6 (1.1%) Doctorate degree, 8 (1.4%) Professional degree	524 (94.2%) USA	74 (13.3%) under $25k, 124 (22.3%) $25k-49k, 114 (20.5%) $50-74K, 90 (16.2%) $75-99K, 108 (19.4%) $100-149K, 46 (8.3%) over $150K	88 (15.8%) identified as extremely liberal, 106 (19.1%) identified as mostly liberal, 89 (16.0%) identified as somewhat liberal, 142 (25.5%) identified neither liberal nor conservative, 57 (10.3%)	18-79 (*M* =37.50, *SD* = 12.51)

looking, 19 (3.4%) Not employed and not looking

identified as somewhat conservative, 48 (8.6%) identified as mostly conservative, 26 (4.7%) identified as extremely conservative

Table S3

Perceived Target Demographic Information Across Studies.

	Target Age	Target Race	Target Gender
Initial Supplemental Study	N/A	N/A	N/A
Study 1	15 (2.7%) 26-30, 39 (7.0%) 31-35, 73 (13.0%) 36-40, 90 (16.1%) 41-45, 111 (19.8%) 46-50, 120 (21.4%) 51-55, 80 (14.3%) 56-60, 24 (4.3%) 61-65, 7 (1.3%) 66-70, 1 (0.2%) 71-75	4 (0.7%) American-Indian or Alaskan Native, 3 (0.5%) Asian, 9 (1.6%) Black or African American, 4 (0.7%) Hispanic or Latinx, 535 (95.5%) White or Caucasian, 5 (0.9%) Other	529 (94.5%) Man, 27 (4.8%) Woman
Study 2	17 (3.1%) 26-30, 62 (11.4%) 31-35, 85 (15.7%) 36-40, 89 (16.4%) 41-45, 84 (15.5%) 46-50, 106 (19.6%) 51-55, 69 (12.7%) 56-60, 27 (5.0%) 61-65, 2 (0.4%) 66-70, 1 (0.2%) 71-75	5 (0.9%) American-Indian or Alaskan Native, 11 (2.0%) Asian, 28 (5.2%) Black or African American, 8 (1.5%) Hispanic or Latinx, 2 (0.4%) Native Hawaiian or Other Pacific Islander, 483 (89.1%) White or Caucasian, 5 (0.9%) Other	489 (90.2%) Man, 50 (9.2%) Woman
Study 3	94 (16.9%) 26-30, 141 (25.4%) 31-35, 117 (21.0%) 36-40, 85 (15.3%) 41-45, 44 (7.9%) 46-50, 44 (7.9%) 51-55, 23 (4.1%) 56-60, 6 (1.1%) 61-65, 2 (0.4%) 66-70	13 (2.3%) American-Indian or Alaskan Native, 7 (1.3%) Asian, 36 (6.5%) Black or African American, 3 (0.5%) Hispanic or Latinx, 1 (0.2%) Native Hawaiian or Other Pacific Islander, 482 (86.7%) White or Caucasian, 14 (2.5%) Other	541 (97.3%) Man, 8 (1.4%) Woman

Note. The target in Study 1 and Study 2 was a fictitious professor (Dr. Johnson). The target in Study 3 was a fictitious coworker (Alex Johnson).

Additional Measures, Analyses, and Results for Study 1

Method

Measures

The descriptives and correlations for all supplemental outcome variables in Study 1 are available in Table S4. In addition to our measures in the main document, participants also completed measures assessing their perceptions of Dr. Johnson, including *likability* (1 item; 1 – *Not likable at all* to 7 – *Very likable*; e.g., “How likable do you find Dr. Johnson”; modified from Wallace et al., 2020), *perceived similarity* (4 items; 1 – *Strongly disagree* to 5 – *Strongly agree*; e.g., “This person’s values and my values are similar”; modified from Goldstein et al., 2014), and participants completed the *Positive and Negative Affect Scale* (PANAS; Watson et al., 1988). In this 20-item scale participants rated (1 – *very slightly or not at all* to 5 – *extremely*) how positive (10 items; e.g., proud) and negative (10 items; e.g., nervous) Dr. Johnson made them feel. In addition, participants assessed Dr. Johnson on *perceived prejudice* (14 items; 1 – *Strongly disagree* to 5 – *Strongly agree*; e.g., “Dr. Johnson is…prejudiced”; Aikawa et al., 2026), and anticipated *race-based* (3 items; 1 – *Not at all* to 7 – *Very much*; e.g., “If you were enrolled in a class with Dr. Johnson, do you think Dr. Johnson would… discount your opinion in class because of your race?”; modified from Chaney et al., 2021) and *gender-based* (3 items; 1 – *Not at all* to 7 – *Very much*; e.g., “If you were enrolled in a class with Dr. Johnson, do you think Dr. Johnson would… discount your opinion in class because of your gender?”; modified from Chaney et al., 2021) *classroom mistreatment*.

Table S4

Supplemental Measure information and statistics, reliabilities, and correlations for Study 1.

Measure	M (SD)	1	2	3	4	5	6	7
1. Likability	5.01 (1.56)	(NA)						
2. Perceived Similarity	2.55 (0.80)	.57***	(0.90)					
3. Positive Affect	2.01 (0.82)	.47***	.33***	(0.92)				
4. Negative Affect	1.60 (0.70)	-.34***	.34***	.24***	(0.93)			
5. Perceived Prejudice	3.67 (0.82)	-.63***	-.55***	-.38***	.42***	(0.90)		
6. Race-based Classroom Mistreatment	2.21 (1.37)	-.30***	-.31***	-.11**	.29***	.47***	(0.96)	
7. Gender-based Classroom Mistreatment	2.56 (1.46)	-.37***	-.40***	-.13**	.40***	.56***	.72***	(0.95)

Note. The reliability for each scale is shown on the diagonal. $^{**}p < .01$, $^{***}p < .001$

Results

To examine the influence of the flags on perceptions of the professor, we conducted separate one-way ANOVAs (No flag vs. Denver Broncos flag vs. American flag) for each supplemental outcome. Means, standard deviation, contrasts between conditions, and full ANOVA results are reported in Table S5. Bonferroni corrections were applied to account for multiple comparisons. We found significant effects for all variables (all *ps* < .001), except for positive affect (*p* = .067; see Table S5). Compared to the no flag and American flag conditions, displaying the Broncos flag led to higher perceived likability (*ps* < .001), higher perceptions of similarity (*ps* < .010), less negative affect (*ps* < .001), and less perceived prejudice (*ps* < .003; see Table S5). There were no significant differences between the no flag and American flag condition for likability, perceived similarity, and negative affect (*ps* > .703). In addition, participants anticipated more race and gender-based mistreatment, and reported higher perceptions of prejudice perceived when assigned to the American flag (vs. no flag and Broncos flag) conditions (*ps* < .001; see Table S5). There were no significant

differences between the no flag and Broncos flag conditions for race and gender-based classroom mistreatment (*ps* > .999; see Table S5).

Table S5

Study 1 Supplemental Means, Standard Deviation, and One-way ANOVA across conditions.

Means, Standard Deviation, One-Way ANOVA Results Across Condition									
Measures	No Flag		Denver Broncos Flag		American Flag				
	M	SD	M	SD	M	SD	*F* (556)	*p*	η_p^2
*Likability	4.65	1.50	5.68	1.39	4.69	1.58	27.90	<.001	.091
*Perceived Similarity	2.50	0.75	2.75	0.76	2.41	0.86	9.31	<.001	.032
Positive Affect	1.91	0.79	2.11	0.88	2.00	0.79	2.71	.067	.010
Negative Affect	1.73	0.74	1.34	0.57	1.74	0.70	21.13	<.001	.071
Perceived Prejudice	3.62	0.75	3.34	0.78	4.02	0.77	37.65	<.001	.119
Race-based Classroom Mistreatment	2.07	1.27	1.94	1.11	2.62	1.60	13.44	<.001	.046
Gender-based Classroom Mistreatment	2.32	1.38	2.36	1.30	2.97	1.61	11.93	<.001	.041

Differences between Flag Conditions									
Measures	No Flag vs. Denver Broncos			No Flag vs. American			Denver Broncos vs. American		
	MD (*SE*)	*p*	*d*	*MD* (*SE*)	*p*	*d*	*MD* (*SE*)	*p*	*d*
Likability	1.02 (0.16)	<.001	-0.71	0.04 (0.15)	1.00	-0.03	0.98 (0.15)	<.001	0.67
Perceived Similarity	0.25 (0.08)	.009	-0.33	0.10 (0.08)	.704	0.12	0.34 (0.08)	<.001	0.42
Positive Affect	0.20 (0.09)	.061	-0.24	0.09 (0.09)	.927	-0.11	0.11 (0.09)	.557	0.13
Negative Affect	0.39 (0.07)	<.001	0.59	0.01(0.07)	1.00	-0.01	0.40 (0.07)	<.001	-0.62
Perceived Prejudice	0.28 (0.08)	.002	0.36	0.40 (0.08)	<.001	-0.53	0.68 (0.08)	<.001	-0.88
Race-based Classroom Mistreatment	0.13 (0.14)	1.00	0.11	0.55 (0.14)	<.001	-0.38	0.68 (0.14)	<.001	-0.49
Gender-based Classroom Mistreatment	0.04 (0.15)	1.00	-0.03	0.64 (0.15)	<.001	-0.43	0.61 (0.15)	<.001	-0.42

Note. MD = Mean Difference. SE = Standard Error. *df = 557

Moderation by Participant Gender

In addition to the primary results in the main manuscript, we also explored whether participant gender moderated the effects across studies. Specifically, we conducted separate 2 (Participant Gender: men vs. women) x 3 (Flag Condition: no flag vs. Denver Broncos flag vs. American flag) between-subjects ANOVAs on our outcome variables. See Table S6 for means and standard deviations, and Table S7 for contrasts between conditions and full ANOVA results. Bonferroni corrections were applied to account for multiple comparisons.

Table S6

Study 1 Means and Standard Deviations for Participant Gender and Flag Condition.

Means and Standard Deviation Across Condition												
Measures	No Flag				Denver Broncos Flag				American Flag			
	Men (*n* = 51)		Women (*n* = 133)		Men (*n* = 54)		Women (*n* = 131)		Men (*n* = 45)		Women (*n* = 145)	
	M	SD	M	SD	M	SD	M	SD	M	SD	M	SD
Comfort and Interest	4.43	1.15	3.75	1.29	4.93	1.19	4.45	1.27	4.41	1.68	3.68	1.43
Perceived Racism	2.16	0.75	2.43	0.75	2.04	072	2.27	0.70	276	1.05	317	0.81
Perceived Sexism	2.28	0.75	256	0.84	2.19	084	2.60	0.70	2.56	0.94	3.24	0.86
Race-based Classroom Mistreatment	1.87	1.24	2.15	1.28	1.79	1.04	2.00	1.13	2.21	143	2.74	1.64
Gender-based Classroom Mistreatment	1.87	1.26	2.50	1.38	1.92	1.19	2.54	1.30	1.79	1.14	3.3	1.56
Professor SDO	3.34	0.80	3.50	0.94	3.31	0.89	3.37	0.99	3.88	1.04	4.43	0.91
Professor Selection	4.00	1.27	3.68	1.37	4.72	1.19	4.70	1.30	4.09	1.64	3.38	1.33

Measures	No Flag		Denver Flag		American Flag		Men		Women	
	M	SD	M	SD	M	SD	M	SD	M	SD
Comfort and Interest	3.94	1.29	4.59	1.26	3.85	1.52	4.60	136	3.95	1.38
Perceived Racism	2.35	076	2.21	071	307	0.89	2.29	0.89	2.64	0.85
Perceived Sexism	2.49	0.83	2.48	0.77	3.08	093	2.33	0.85	2.81	0.87
Race-based Classroom Mistreatment	2.07	1.27	1.94	1.11	2.62	1.60	1.94	1.24	2.31	1.41
Gender-based Classroom Mistreatment	2.32	1.38	2.36	1.30	2.97	1.61	1.86	1.19	2.81	1.48
Professor SDO	3.45	0.91	3.35	0.96	4.30	0.97	3.49	0.94	3.79	1.06
Professor Selection	3.77	1.35	4.71	1.27	3.55	1.44	4.29	1.39	3.90	1.45

Table S7

Study 1 Between-Subjects Results for Flag condition.

Measures	Flag Condition			Participant Gender Condition					Flag × Participant Gender		
	F (553)	*p*	η_p^2	*F* (553)	*p*	η_p^2	*MD (SE)*	*d*	*F* (553)	*p*	η_p^2
Comfort and Interest	10.77	<.001	.042	24.00	<.001	.038	0.63 (0.13)	0.48	0.38	.684	.001
Perceived Racism	43.53	<.001	.136	17.06	<.001	.030	0.31 (0.08)	-0.40	0.53	.591	.002
Perceived Sexism	17.18	<.001	.058	33.91	<.001	.058	0.46 (0.08)	-0.56	2.19	.113	.008
Race-based Classroom Mistreatment	7.51	<.001	.026	7.16	.008	.013	0.34 (0.13)	-0.28	0.59	.555	.002
Gender-based Classroom Mistreatment	3.21	.041	.011	50.46	<.001	.084	0.93 (0.13)	-0.70	5.26	.005	.019
Professor SDO	32.59	<.001	.105	8.26	.004	.015	0.26 (0.09)	-0.30	2.80	.062	.010
Professor Selection	23.76	<.001	.079	7.43	.007	.013	0.35 (0.13)	0.27	2.39	.093	.009

Differences between Flag Conditions									
Measures	No Flag vs. Broncos Flag			No Flag vs. American			Broncos Flag vs. American		
	MD (*SE*)	*p*	*d*	*MD* (*SE*)	*p*	*d*	*MD* (*SE*)	*p*	*d*
Comfort and Interest	0.60 (0.15)	<.001	-0.51	0.05 (0.16)	1.00	0.06	0.65 (0.16)	<.001	0.53
Perceived Racism	0.14 (0.09)	.390	0.20	0.67 (0.09)	<.001	-0.87	0.81 (0.09)	<.001	-1.08
Perceived Sexism	0.03 (0.09)	1.00	0.01	0.48 (0.10)	<.001	-0.68	0.51 (0.10)	<.001	-0.71
Race-based Classroom Mistreatment	0.12 (0.16)	1.00	0.11	0.47 (0.16)	.010	-0.38	0.58 (0.16)	<.001	-0.49
Gender-based Classroom Mistreatment	0.05 (0.16)	1.00	-0.03	0.38 (0.16)	.060	-0.43	0.33 (0.16)	.119	-0.42
Professor SDO	0.08 (0.11)	1.00	0.11	0.73 (0.11)	<.001	-0.90	0.82 (0.11)	<.001	-0.98
Professor Selection	0.87 (0.16)	<.001	-0.72	0.11 (0.16)	1.00	0.16	0.98 (0.16)	<.001	0.86

Note. MD = Mean Difference. SE = Standard Error.

We found a significant main effect of participant gender across all outcome variables (all *ps* < .009; see Table S7). Women (versus men) participants reported less comfort and interest interacting with the professor, were less likely to select the professor,

anticipated more race- and gender-based mistreatment, and perceived the professor as more racist, more sexist, and higher in SDO. Additionally, we found a significant main effect of flag condition across all outcome variables (all *ps* $< .009$; see Table S7). Participants reported more comfort and interest interacting with, and more of a willingness to select, the professor in the Broncos flag versus no flag condition (*ps* $< .001$; see Table S7). Compared to the no flag and Broncos flag conditions, participants perceived the professor in the American flag condition as more racist (*ps* $< .001$), more sexist (*ps* $< .001$), higher in SDO (*ps* $< .001$), and expected more race-based classroom mistreatment (*ps* $< .011$; see Table S7). Moreover, participants in the American flag (versus Broncos flag) condition reported less comfort and interest interacting with, and less of a willingness to select, the professor (*ps* $< .001$). Lastly, we only found a significant participant gender by flag condition interaction for gender-based mistreatment ($p = .005$; see Table S7). In the no flag condition, *mean difference* = 0.63, SE = 0.23, $p = .005$, $d = 0.48$, Broncos flag condition, *mean difference* = 0.62, SE = 0.22, $p = .005$, $d = 0.58$, and American flag condition, *mean difference* = 1.54, SE = 0.23, $p < .001$, $d = 1.13$, women participants anticipated more gender-based classroom mistreatment than men participants.

Perceptions of Professor Race

We then examined whether the minority of participants who perceived the professor as other than White (vs. White) responded differently to the flag conditions. Specifically, we conducted separate 2 (Professor Race: other race vs. White) x 3 (Flag Condition: no flag vs. Denver Broncos flag vs. American flag) between-subjects ANOVAs on our outcome variables. See Table S8 for means and standard deviations, and Table S9 for contrasts between conditions and full ANOVA results. Bonferroni corrections were applied to account for multiple comparisons.

Table S8

Study 1 Means and Standard Deviations for Differences in Perceptions of Professor Race.

Means and Standard Deviation Across Condition												
Measures	No Flag				Denver Broncos Flag				American Flag			
	Other Race (n = 11)		White (n = 173)		Other Race (n = 7)		White (n = 178)		Other Race (n = 7)		White (n = 183)	
	M	SD	M	SD	M	SD	M	SD	M	SD	M	SD
Comfort and Interest	4.42	1.10	3.91	1.30	3.95	1.98	4.62	1.23	5.52	1.60	3.79	1.49
Perceived Racism	2.39	0.47	2.35	0.78	2.07	0.73	2.21	0.71	2.71	0.87	3.09	0.89
Perceived Sexism	2.30	0.58	2.50	0.84	2.07	0.59	2.49	0.77	2.71	0.85	3.09	0.93
Professor SDO	2.86	0.92	3.49	0.89	4.05	0.20	3.32	0.97	3.63	1.24	4.33	0.96
Professor Selection	3.91	1.14	3.76	1.36	4.57	1.13	4.71	1.27	4.86	1.68	3.50	1.41

Measures	No Flag		Broncos Flag		American Flag		Other Race		White	
	M	SD	M	SD	M	SD	M	SD	M	SD
Comfort and Interest	3.94	1.29	4.59	1.26	3.85	1.52	4.60	1.58	4.10	1.39
Perceived Racism	2.35	0.76	2.21	0.71	3.07	0.89	2.39	0.69	2.56	0.89
Perceived Sexism	2.49	0.83	2.48	0.77	3.08	0.93	2.35	0.68	2.70	0.89
Professor SDO	3.45	0.91	3.35	0.96	4.30	0.97	3.41	1.01	3.72	1.04
Professor Selection	3.77	1.35	4.71	1.27	3.55	1.44	4.36	1.32	3.99	1.45

Table S9

Study 1 Between-Subjects Results for Flag condition.

Measures	Flag Condition			Professor Race Condition					Flag × Professor Race		
	F (553)	p	η_p^2	F (553)	p	η_p^2	MD (SE)	d	F (553)	p	η_p^2
Comfort and Interest	1.11	.331	.004	3.52	.061	.006	0.53 (0.28)	0.33	5.32	.005	.019
Perceived Racism	6.63	.001	.023	0.92	.338	.002	0.16 (0.17)	-0.21	0.54	.582	.002
Perceived Sexism	4.30	.014	.015	3.59	.059	.006	0.33 (0.18)	-0.44	0.17	.847	.001
Professor SDO	6.34	.002	.022	1.03	.310	.002	0.20 (0.20)	-0.30	5.29	.005	.019
Professor Selection	2.94	.054	.010	2.62	.106	.005	0.46 (0.28)	0.27	2.43	.089	.009

Differences between Flag Conditions									
Measures	No Flag vs. Broncos Flag			No Flag vs. American			Broncos Flag vs. American		
	MD (SE)	p	d	MD (SE)	p	d	MD (SE)	p	d

Comfort and Interest	0.12 (0.33)	1.00	-0.51	0.49 (0.33)	.428	0.06	0.37 (0.37)	.937	0.53
Perceived Racism	0.2 (0.20)	.739	0.20	0.53 (0.20)	.021	-0.87	0.76 (0.22)	.001	-1.08
Perceived Sexism	0.11 (0.21)	1.00	0.01	0.51 (0.21)	.046	-0.68	0.62 (0.23)	.021	-0.71
Professor SDO	0.51 (0.23)	.084	0.11	0.80 (0.23)	.002	-0.90	0.29 (0.26)	.785	-0.98
Professor Selection	0.81 (0.33)	.047	-0.72	0.34 (0.33)	.912	0.16	0.47 (0.37)	.618	0.86

Note. MD = Mean Difference. SE = Standard Error.

We found a significant main effect of flag condition for all outcome variables (all *ps* < .015), except for comfort and interest (*p* = .331) and professor selection (*p* = .054; see Table S9). Compared to the no flag and Broncos flag conditions, participants in the American flag condition perceived the professor as more racist (*ps* < .022) and more sexist (ps < .047; see Table S9). Participants also perceived the professor in the American flag versus no flag condition as higher in SDO (*p* = .002); however, this effect was not found in the Broncos flag versus American flag comparison (*p* = .785; see Table S9).

Moreover, we found a significant flag condition by professor race interaction on comfort and interest (*p* = .005) and professor SDO (*p* = .005; see Table S9). In the American flag condition, participants who perceived the professor as White (vs. other race) reported less comfort and interest interacting with the professor, *mean difference* = 1.74, standard error = 0.52, *p* < .001, *d* = 1.12. This effect was not found for the no flag (*p* = .219) and Broncos flag (*p* = .203) conditions. Lastly, for professor SDO, in the no flag condition, participants who perceived the professor as White (vs. other race) also perceived the professor as higher in SDO, *mean difference* = 0.63, standard error = 0.29, *p* = .032, *d* = 0.69. In contrast, in the Broncos flag condition, participants who perceived the professor as White (vs. other race) also perceived the professor as lower in SDO, *mean difference* = 0.73, standard error = 0.36, *p* =

.044, *d* = 1.05. We did not find any significant differences between perceptions of professor race in the American flag condition (*p* = .053). We also did not find any significant main effects of professor race (all *ps* > .058; see Table S9).

Perceptions of Professor Gender

We also examined whether the minority of participants who perceived the professor as a woman (vs. man) responded differently to the flag conditions. Specifically, we conducted separate 2 (Professor Gender: woman vs. man) x 3 (Flag Condition: no flag vs. Denver Broncos flag vs. American flag) between-subjects ANOVAs on our outcome variables. See Table S10 for means and standard deviations, and Table S11 for contrasts between conditions and full ANOVA results. Bonferroni corrections were applied to account for multiple comparisons.

Table S10

Study 1 Means and Standard Deviations for Differences in Perceptions of Professor Gender.

Measures	Means and Standard Deviation Across Condition											
	No Flag				Denver Broncos Flag				American Flag			
	Woman (n = 11)		Man (n = 173)		Woman (n = 7)		Man (n = 178)		Woman (n = 7)		Man (n = 183)	
	M	SD	M	SD	M	SD	M	SD	M	SD	M	SD
Comfort and Interest	3.88	1.27	4.50	1.36	4.56	1.25	5.25	1.40	3.82	1.51	5.00	1.58
Perceived Racism	2.38	0.76	2.07	0.74	2.22	0.71	1.78	0.67	3.07	0.89	3.15	0.60
Perceived Sexism	2.08	0.59	2.53	0.84	1.91	0.94	2.50	0.75	3.35	0.49	3.07	0.93
Race-based Classroom Mistreatment												
Gender-based Classroom Mistreatment												
Professor SDO	3.50	0.90	3.05	0.85	3.36	0.95	3.09	1.05	4.30	0.96	4.15	1.35
Professor Selection	3.69	1.35	4.50	1.15	4.72	1.27	4.38	1.19	3.55	1.45	3.60	0.89

Measures	No Flag		Broncos Flag		American Flag		Woman		Man	
	M	SD	M	SD	M	SD	M	SD	M	SD
Comfort and Interest	3.94	1.29	4.59	1.26	3.85	1.52	4.09	1.39	4.77	1.40
Perceived Racism	2.35	0.76	2.21	0.71	3.07	0.89	2.57	0.88	2.17	0.82
Perceived Sexism	2.49	0.83	2.48	0.77	3.08	0.93	2.24	0.83	2.71	0.88
Professor SDO	3.45	0.91	3.35	0.96	4.30	0.97	3.73	1.03	3.24	1.04
Professor Selection	3.77	1.35	4.71	1.27	3.55	1.44	3.98	1..46	4.32	1.14

Table S11

Study 1 Between-Subjects Results for Flag condition.

Measures	Flag Condition			Professor Gender Condition					Flag × Professor Gender		
	F (553)	p	η_p^2	F (553)	p	η_p^2	MD (SE)	d	F (553)	p	η_p^2
Comfort and Interest	2.91	.056	.010	8.47	.004	.015	0.83 (0.29)	0.77	0.32	.726	.001
Perceived Racism	12.67	<.001	.044	1.86	.173	.003	0.23 (0.17)	0.47	0.68	.507	.002
Perceived Sexism	10.26	<.001	.036	2.08	.150	.004	0.25 (0.18)	0.55	1.81	.165	.006
Professor SDO	8.53	<.001	.030	2.14	.144	.004	0.29 (0.20)	0.48	0.22	.801	.001

Professor Selection	3.16	.043	.011	0.37	.543	.001	0.17 (0.28)	-0.26	2.09	.125	.007

	Differences between Flag Conditions								
Measures	No Flag vs. Broncos Flag			No Flag vs. American			Broncos Flag vs. American		
	MD (*SE*)	*p*	*d*	*MD* (*SE*)	*p*	*d*	*MD* (*SE*)	*p*	*d*
Comfort and Interest	0.72 (0.30)	.049	-0.51	0.22 (0.35)	1.00	0.06	0.50 (0.39)	.626	-0.53
Perceived Racism	0.22 (0.17)	.591	0.20	0.88 (0.20)	<.001	-0.87	1.11 (0.23)	<.001	-1.08
Perceived Sexism	0.10 (0.18)	1.00	0.01	0.90 (0.22)	<.001	-0.68	1.01 (0.24)	<.001	-0.71
Professor SDO	0.05 (0.21)	1.00	0.11	0.95 (0.24)	<.001	-0.90	1.00 (0.27)	<.001	-0.98
Professor Selection	0.46 (0.30)	.374	-0.72	0.52 (0.35)	.406	0.16	0.98 (0.39)	.039	0.86

Note. MD = Mean Difference. SE = Standard Error.

We only found a significant main effect of professor gender for comfort and interest (*p* = .004; see Table S11), such that participants who perceived the professor as a man reported more comfort and interest interacting with the professor than participants who perceived the professor as a woman. Moreover, we found a significant main effect of flag condition for all outcome variables (all *ps* < .044), except for comfort and interest (*p* = .056; see Table S11). Compared to the no flag and Broncos flag conditions, participants in the American flag condition perceived the professor as more racist, more sexist, and higher in SDO (all *ps* < .001; see Table S11). Participants were also more willing to select the professor in the Broncos flag versus American flag condition (*p* = .039; see Table S11). We did not find any significant flag condition by professor gender interactions (all *ps* > .124; see Table S11).

Moderation by Participant Political Orientation

Next, we explored whether participants reported political orientation (1 = *Extremely Liberal* to 7 = *Extremely Conservative*) moderated the effects of flag condition. Thus, we ran a regression model with flag condition dummy coded and the no flag condition as the reference group, political orientation, and all interactions. Initial analyses revealed only a significant Flag Condition x Political Orientation interaction for the comparison between the no flag and Denver Broncos flag conditions on perceived similarity (p = .017; see Table S12). We therefore probed the significant Flag condition (no flag vs. Denver Broncos flag) x Political Orientation interaction on perceived similarity by examining the effect of the no flag versus the Denver Broncos flag condition at one standard deviation below and above the mean on political orientation (see Table S12 for simple effects). At one standard deviation above the mean, participants perceived the professor in the Denver Broncos flag versus no flag condition as more similar to them (p < .001); however, this effect was not found for participants at one standard deviation below the mean on political orientation (p = .548; see Table S12).

We also observed significant Flag Condition x Political Orientation interactions for the comparison between the no flag and American flag conditions on all outcome variables (all *ps* < .040), except for race-based classroom mistreatment (p = .251) and positive affect (p = .695; see Table S12). We probed the significant Flag Condition (no flag vs. American flag) x Political Orientation interactions by examining the effect of the no flag versus the American flag condition at one standard deviation below and above the mean on political orientation (see Table S12 for simple effects).

At one standard deviation above the mean on political orientation, participants perceived the professor in the American flag versus no flag condition as more similar to them (p = .017); however, this effect was not found for participants at one standard deviation below the mean (p = .548; see Table S12). At one standard deviation below the mean on political orientation, the professor in the American flag versus no flag condition elicited more negative affect (p = .021). At one standard deviation above the mean, the opposite pattern occurred (p = .042), the no flag versus American flag condition elicited more negative affect among conservative leaning participants (see Table S12).

At one standard deviation below on the mean on political orientation, participants liked the professor more in the no flag compared the American flag condition; however, this effect did not reach significance (p = .098). At one standard deviation above the mean on political orientation, the effect was also non-significant (p = .063), but participants were showing the opposite pattern, liking the professor more in the American flag versus no flag condition. For comfort and interest, a similar pattern emerged. At one below the mean on political orientation, participants expressed more comfort and interest in the no flag compared to American flag condition (p = .007); however, this effect was not found for participants at one standard deviation above the mean on political orientation (p = .105) At one standard deviation below the mean on political orientation, participants viewed the professor as lower prejudice, lower in racism, lower in sexism, lower in expected gender-based classroom mistreatment, and lower in SDO in the no flag compared to the American flag condition (all ps < .001). At one standard deviation above the mean on political orientation, participants also viewed the professor as lower in racism (p < .001), sexism (p = .004), and SDO (p < .001) in the no flag versus American flag condition; however, these effects were weaker compared to more liberal participants (i.e., those at one standard

deviation below the mean). Among participants at one standard deviation above the mean on political orientation, there was no significant difference between the no flag and American flag condition on perceived prejudice and gender-based classroom mistreatment. Lastly, for professor selection, at one standard deviation below the mean, participants indicated a higher likelihood of selecting the professor in the no flag versus the American flag condition ($p = .002$); however, this effect was not significant for participants at one standard deviation above the mean ($p = .529$; see Table S12).

Table S12

Interactions with Political Orientation and Flag Conditions from Study 1.

Measures	Flag Condition (No Flag vs. Denver Broncos Flag) × Political Orientation			Flag Condition (No Flag vs. American Flag) × Political Orientation		
	b (SE)	*t* (553)	*p*	*b* (SE)	*t* (553)	*p*
*Perceived Similarity	0.14 (0.06)	2.40	.017	0.29 (0.06)	4.65	< .001
Positive Affect	-0.07 (0.06)	-1.09	.278	-0.03 (0.07)	-0.39	.695
Negative Affect	-0.04 (0.05)	-0.72	.472	-0.17 (0.05)	-3.02	.003
*Likability	-0.06 (0.11)	-0.57	.572	0.29 (0.12)	2.44	.015
Comfort and Interest	-0.05 (0.10)	-0.46	.644	0.32 (0.11)	3.00	.003
Perceived Prejudice	-0.04 (0.06)	-0.63	.526	-0.23 (0.06)	-3.77	<.001
Perceived Racism	-0.01 (0.06)	-0.17	.867	-0.19 (0.06)	-3.05	.002
*Perceived Sexism	-0.07 (0.06)	-1.16	.247	-0.19 (0.07)	-2.88	.004
Race-based Classroom Mistreatment	-0.07 (0.11)	-0.63	.530	-0.13 (0.11)	-1.15	.251
Gender-based Classroom Mistreatment	-0.09 (0.11)	-0.86	.391	-0.24 (0.12)	-2.07	.039
*Professor SDO	0.00 (0.07)	0.03	.976	-0.21 (0.08)	-2.77	.006
*Professor Selection	0.06 (0.10)	0.56	.579	0.28 (0.11)	2.57	.011
Simple of Effects of Flag Condition (No Flag vs. Denver Broncos Flag) at 1 Standard Deviation Above and Below the Mean on the Political Orientation Scale						
Measures	1 Standard Deviation Below			1 Standard Deviation Above		
	b (SE)	*t* (553)	*p*	*b* (SE)	*t* (553)	*p*
Perceived Similarity	0.07 (0.11)	0.61	.548	0.44 (0.11)	3.96	< .001
Simple of Effects of Flag Condition (No Flag vs. American Flag) at 1 Standard Deviation Above and Below the Mean on the Political Orientation Scale						
Measures	1 Standard Deviation Below			1 Standard Deviation Above		
	b (SE)	*t* (553)	*p*	*b* (SE)	*t* (553)	*p*
*Perceived Similarity	-0.48 (0.11)	0.60	.548	0.26 (0.11)	2.39	.017
Negative Affect	0.23 (0.10)	2.32	.021	-0.20 (0.10)	-2.04	.042
Likability	-0.36 (0.22)	-1.66	.098	0.40 (0.21)	1.86	.063
Comfort and Interest	-0.52 (0.19)	-2.69	.007	0.31 (0.19)	1.62	.105
Perceived Prejudice	0.71 (0.11)	6.43	<.001	0.11 (0.11)	1.04	.299
Perceived Racism	0.98 (0.11)	8.62	<.001	0.48 (0.11)	4.30	<.001
Perceived Sexism	0.85 (0.12)	7.00	<.001	0.35 (0.12)	2.91	.004
Gender-based Classroom Mistreatment	0.97 (0.21)	4.62	<.001	0.35 (0.21)	1.68	.094
Professor SDO	1.13 (0.14)	8.06	<.001	0.57 (0.14)	4.13	<.001
Professor Selection	-0.60 (0.20)	-3.06	.002	0.12 (0.19)	0.63	.529

Note. *b* = unstandardized regression coefficient. SE = Standard Error. *df = 554.

Additional Measures, Analyses, and Results for Study 2

In addition to our main analyses, we also probed the participant race X flag condition interaction to examine whether the strength of the flag effect differs between White and Black/Latine participants (see Table S13). Compared to the no flag condition, White participants in the American flag condition perceived the professor as more racist, more sexist, and higher in SDO (all *ps* < .003; see Table S13). We did not find any significant flag condition differences on comfort and interest and professor selection (*ps* > .999; see Table S13). Compared to the inclusive flag condition, White participants in the American flag condition reported less comfort and interest interacting with, and a lower likelihood of selecting, the professor (*ps* < .001). They also perceived the professor as more racist, more sexist, and higher in SDO (all *ps* < .001; see Table S13). Compared to the no flag condition, White participants in the inclusive flag condition reported more comfort and interest interacting with, and a higher likelihood of selecting, the professor (*ps* < .001). They also perceived the professor as less racist, less sexist, and lower in SDO (all *ps* < .001; see Table S13).

For Black/Latine participants, compared to the no flag and inclusive flag conditions, participants in the American flag condition perceived the professor as more racist, more sexist, and higher in SDO (all *ps* < .001; see Table S13). These participants also reported less comfort and interest interacting with, and a lower likelihood of selecting, the professor (all *ps* < .018; see Table S13). Compared to the no flag condition, Black/Latine participants in the inclusive flag condition reported more comfort and interest

interacting with, and a higher likelihood of selecting, the professor (*ps* < .001). They also perceived the professor as less racist, less sexist, and lower in SDO (all *ps* < .001; see Table S13).

Table S13

Measures	Participant Race X Flag Condition Interaction																	
	White									Black/Latine								
	No Flag vs. Inclusive Flag			No Flag vs. American Flag			Inclusive Flag vs. American Flag			No Flag vs. Inclusive Flag			No Flag vs. American Flag			Inclusive Flag vs. American Flag		
	MD (*SE*)	*p*	*d*	*MD* (*SE*)	*p*	*d*	*MD* (*SE*)	*p*	*d*	*MD* (*SE*)	*p*	*d*	*MD* (*SE*)	*p*	*d*	*MD* (*SE*)	*p*	*d*
Comfort and Interest	1.60 (0.22)	< .001	-1.18	0.06 (.22)	1.00	-0.04	1.54 (0.22)	< .001	1.04	1.61 (0.21)	< .001	-1.29	0.72 (0.20)	.001	0.50	2.32 (0.20)	< .001	1.75
Perceived Racism	0.68 (0.13)	<.001	0.94	0.53 (0.13)	<.001	-0.65	1.21 (0.13)	<.001	-1.45	0.79 (0.12)	<.001	1.06	0.80 (0.12)	<.001	-0.90	1.60 (0.11)	<.001	-1.97
Perceived Sexism	0.74 (0.13)	<.001	0.94	0.45 (0.13)	.002	-0.49	1.19 (0.13)	<.001	-1.35	0.71 (0.12)	<.001	0.90	0.62 (0.12)	<.001	-0.72	1.32 (0.12)	<.001	-1.70
Professor SDO	1.65 (0.17)	<.001	2.02	0.92 (0.17)	<.001	-0.82	2.56 (0.17)	<.001	2.54	1.41 (0.16)	<.001	1.44	0.76 (0.15)	<.001	-0.62	2.17 (0.15)	<.001	-1.80
Professor Selection	1.61 (0.23)	<.001	-1.14	0.08 (0.23)	1.00	0.06	1.70 (0.23)	<.001	1.08	1.46 (0.21)	<.001	-1.06	0.58 (0.21)	.017	0.41	2.03 (0.21)	<.001	1.39

Additional Measures, Analyses, and Results for Study 2: Part 2

We next examined whether participants reported political orientation (1 = *Extremely Liberal* to 7 = *Extremely Conservative*) moderated the race and flag condition, we ran a regression model with condition dummy and the no flag condition as the reference group, race (0 = White participants and 1 = Black/Latine participants), Political Orientation, and all interactions. For all outcome measures, we examined two-way interactions between condition dummy coded (with no flag condition as the reference group), and political orientation (all two-way interactions are reported in Table S14). When interactions were significant, we examined the effect of condition at one standard deviation below and above the mean on political orientation (see Table S15).

At one standard deviation below (*ps* $< .001$) and above (*ps* $< .005$) the mean on political orientation, participants perceived the professor in the inclusive flag versus no flag condition as more likable and reported more comfort and interest interacting; however, these effects were larger for liberal leaning participants (see Table S15).

At one standard deviation below (*ps* < .001) and above (*ps* < .001) the mean on political orientation, participants perceived the professor in the professor in the no flag versus inclusive flag condition as more prejudiced and higher in SDO; however, these effects were larger for liberal leaning participants (see Table S15). At one standard deviation below the mean on political orientation, participants perceived the professor in the professor in the inclusive flag versus no flag condition as more racist, more sexist, and anticipated more race- and gender-based classroom mistreatment (*ps* < .001); however, these effects was not found for conservative leaning participants (*ps* > .131; see Table S15). At one standard deviation below the mean on political orientation, participants were more willing to select the professor in the inclusive flag versus no flag condition (*p* < .001); however, this effect was not significant for conservative leaning participants (*p* = .079; see Table S15).

At one standard deviation above the mean on political orientation, participants perceived the professor in the American flag versus no flag condition as more likable, reported more comfort and interest interacting, and were more willing to select the professor (*ps* < .037; see Table S15). At one standard deviation below the mean, the opposite pattern emerged, participants perceived the professor in the no flag versus the American flag condition as more likable, reported more comfort and interest interacting, and were more willing to select the professor (*ps* < .001; see Table S15). At one standard deviation below the mean on political orientation, participants perceived the professor in the American flag versus no flag condition as more prejudiced, higher in SDO, and anticipated more gender-based classroom mistreatment (*ps* < .001); however, this effect was not significant at one standard deviation above the mean (*ps* > .156; see Table S15). At one standard deviation below (*p* < .001) and above (*p* < .001) the mean on political orientation, participants perceived the professor in the American flag versus no flag

condition as more racist; however, this effect was larger for liberal leaning participants (see Table S15).

Table S14

Interactions with Political Orientation, Participant Race (Black/Latine vs. White), and Flag Conditions from Study 2.

Testing for 3-way Interactions with Flag Condition, Participant Race, and Political Orientation						
Measures	No flag vs. Inclusive Flag× Race × Political Orientation			No flag vs. American Flag × Race × Political Orientation		
	b (SE)	*t* (530)	*p*	*b* (SE)	*t* (530)	*p*
Likability	0.32 (0.20)	1.56	.119	-0.13 (0.20)	-0.63	.528
Comfort and Interest	0.21 (0.18)	1.22	.224	-0.06 (0.17)	-0.36	.721
Perceived Prejudice	-0.09 (0.12)	-0.81	.418	0.16 (0.12)	1.38	.169
Perceived Racism	-0.05 (0.10)	-0.47	.636	0.19 (0.10)	1.82	.070
Perceived Sexism	0.02 (0.11)	0.18	.855	0.28 (0.11)	2.56	.011
Race-based Classroom Mistreatment	-0.13 (0.17)	-0.76	.449	-0.19 (0.17)	-1.13	.261
Gender-based Classroom Mistreatment	-0.10 (0.18)	-0.57	.568	0.07 (0.18)	0.37	.711
Professor SDO	0.06 (0.14)	0.44	.656	-0.13 (0.14)	-0.91	.363
Professor Selection	0.29 (0.18)	1.62	.105	-0.12 (0.18)	-0.66	.512
Testing for 2-way interactions between Condition and Political Orientation						
Measures	No flag vs. Inclusive Flag × Political Orientation			No flag vs. American Flag × Political Orientation		
	b (SE)	*t* (530)	*p*	*b* (SE)	*t* (530)	*p*
Likability	-0.63 (0.10)	-6.23	< .001	0.38 (0.10)	3.72	< .001
Comfort and Interest	-0.42 (0.09)	-4.74	< .001	0.39 (0.09)	4.50	< .001
Perceived Prejudice	0.35 (0.06)	5.96	< .001	-0.14 (0.06)	-2.43	.015
Perceived Racism	0.27 (0.05)	5.24	< .001	-0.11 (0.05)	-2.04	.042
Perceived Sexism	0.24 (0.05)	4.36	< .001	-0.06 (0.05)	-1.07	.287
Race-based Classroom Mistreatment	0.22 (0.09)	2.59	.010	-0.10 (0.09)	-1.14	.256
Gender-based Classroom Mistreatment	0.22 (0.09)	2.43	.015	-0.19 (0.09)	-2.07	.039
Professor SDO	0.17 (0.07)	2.34	.019	-0.30 (0.07)	-4.24	< .001
Professor Selection	-0.50 (0.09)	-5.64	< .001	0.48 (0.09)	5.43	< .001

Table S15

Simple Effects of Flag Condition at 1 Standard Deviation Below and Above the Mean on the Political Orientation Scale from Study 2.

Measures	No Flag (0) versus Inclusive Flag (1)						No Flag (0) versus American Flag (1)					
	Low Political Orientation			High Political Orientation			Low Political Orientation			High Political Orientation		
	b (SE)	*t* (536)	*p*	*b* (SE)	*t* (536)	*p*	*b* (SE)	*t* (536)	*p*	*b* (SE)	*t* (536)	*p*
Likability	3.39 (0.24)	13.85	< .001	0.86 (0.27)	3.20	.002	-1.01 (0.25)	-4.02	< .001	0.62 (0.26)	2.42	.016
Comfort and Interest	2.38 (0.21)	11.16	< .001	0.67 (0.23)	2.87	.004	-1.22 (0.22)	-5.60	< .001	0.47 (0.23)	2.10	.036
Perceived Prejudice	-1.93 (0.14)	13.80	< .001	-0.51 (0.15)	-3.32	.001	0.73 (0.14)	5.14	< .001	0.09 (0.15)	0.58	.561
Perceived Racism	-1.24 (0.13)	-9.91	< .001	-0.16 (0.14)	-1.16	.248	0.93 (0.13)	7.32	< .001	0.44 (0.13)	3.34	< .001
Perceived Sexism	-1.17 (0.13)	-8.88	< .001	-0.22 (0.14)	-1.51	.132	0.69 (0.13)	5.18	< .001	0.40 (0.14)	2.86	.004
Race-based Classroom Mistreatment	-1.06 (0.22)	-4.90	< .001	-0.14 (0.24)	-0.58	.565	0.87 (0.22)	3.94	< .001	0.38 (0.23)	1.68	.094
Gender-based Classroom Mistreatment	-1.08 (0.22)	-4.97	< .001	-0.17 (0.24)	-0.73	.468	1.06 (0.22)	4.77	< .001	0.30 (0.23)	1.33	.185
Professor SDO	-1.84 (0.17)	-10.73	< .001	-0.12 (0.19)	-5.92	< .001	1.40 (0.18)	7.96	< .001	0.26 (0.18)	1.42	.157
Professor Selection	2.45 (0.21)	11.44	< .001	0.41 (0.24)	1.76	.079	-1.35 (0.22)	-6.19	< .001	0.66 (0.23)	2.93	.004

Interactions with Participants SDO, Participant Race, and Flag Conditions: Three-way Interactions from Study 2.

To examine whether participant SDO moderated the race and flag condition, we ran a regression model with flag condition dummy and the no flag condition as the reference group, race (0 = White participants and 1 = Black/Latine participants), participant SDO, and all interactions. We initially only found a significant three-way interaction between the no flag versus inclusive flag contrast, race, and participant SDO predicting likability, and comfort and interest (see Table S16). Although this three-way interaction

did not reach significance for professor selection (*p* = .071) because it showed a similar pattern, we decided to probe this interaction as well.

For likability, at low levels of participant SDO (i.e., one standard deviation below the mean), there was no difference between White and Black/Latine participants in the no flag, *b* = 0.44, *SE* = 0.30, *t* (530) = 1.46, *p* = .145, inclusive flag, *b* = -0.18, *SE* = 0.27, *t* (530) = -0.66, *p* = .510, or the American flag, *b* = -0.27, *SE* = 0.29, *t* (530) = -0.92, *p* = .356, conditions. At high levels of participant SDO (i.e., one standard deviation above the mean) predicting likability, there was again no difference between White and Black/Latine participants in the no flag condition, *b* = 0.04, *SE* = 0.31, *t* (530) = 0.14, *p* = .889. However, at high levels of participant SDO, compared to White participants, Black/Latine participants viewed the professor as more likable in the inclusive flag condition, *b* = 0.90, *SE* = 0.36, *t* (530) = 2.51, *p* = .012, and less likable in the American flag condition, *b* = -0.90, *SE* = 0.26, *t* (530) = -3.46, *p* < .001. Thus, at high (but not low) levels of participant SDO, participant race differences emerged in the inclusive flag and American flag conditions for likability.

For comfort and interest, at low levels of participant SDO (i.e., one standard deviation below the mean), there was no difference between White and Black/Latine participants in the no flag, *b* = -0.03, *SE* = 0.26, *t* (530) = -0.12, *p* = .903, and inclusive flag, *b* = -0.40, *SE* = 0.24, *t* (530) = -1.66, *p* = .100, conditions. However, even at low levels of SDO, there was a significant difference, with White participants reporting higher comfort and interest in the American flag condition than Black and Latine participants, *b* = -0.66, *SE* = 0.25, *t* (530) = -2.64, *p* = .009.

At high levels of participant SDO (i.e., one standard deviation above the mean) predicting comfort and interest, there was again no difference between White and Black/Latine participants in the no flag condition, $b = -0.28$, $SE = 0.26$, $t(530) = -1.07$, $p = .283$. In contrast, at high levels of participant SDO, compared to White participants, Black/Latine participants reported greater comfort and interest in the inclusive flag condition, $b = 0.65$, $SE = 0.31$, $t(530) = 2.11$, $p = .036$, and lower levels of comfort and interest in the American flag condition, $b = -0.89$, $SE = 0.23$, $t(530) = -3.99$, $p < .001$. Regardless of participant SDO levels, Black and Latine participants reported lower comfort and interest than White participants in the American flag condition. However, only at high levels of SDO did Black and Latine participants report higher comfort and interest than White participants in the inclusive flag condition.

Finally, for professor selection, at low levels of participant SDO (i.e., one standard deviation below the mean), there was no difference between White and Black/Latine participants in the no flag, $b = 0.18$, $SE = 0.27$, $t(530) = 0.67$, $p = .505$, inclusive flag, $b = -0.20$, $SE = 0.25$, $t(530) = -0.80$, $p = .426$, or the American flag, $b = 0.22$, $SE = 0.26$, $t(530) = 0.86$, $p = .389$, conditions. At high levels of participant SDO (i.e., one standard deviation above the mean) predicting professor selection, there was again no difference between White and Black/Latine participants in the no flag condition, $b = -0.06$, $SE = 0.27$, $t(530) = -0.21$, $p = .836$. However, at high levels of participant SDO, compared to White participants, Black/Latine participants reported higher (although non-significant) likelihood of selecting the professor in the inclusive flag condition, $b = 0.53$, $SE = 0.32$, $t(530) = 1.67$, $p = .096$, and a significantly lower likelihood of selecting the professor in the American flag condition, $b = -0.70$, $SE = 0.23$, $t(530) = -3.02$, $p = .003$. Therefore, at high (but not low) levels of participant SDO, participant race differences emerged in the inclusive flag and American flag conditions for the likelihood of selecting the professor.

Table S16

Interactions with Participant SDO, Participant Race, and Flag Conditions from Study 2.

Testing for 3-way Interactions with Flag Condition, Participant Race, and Participant SDO						
Measures	No flag vs. Inclusive Flag× Race × Participant SDO			No flag vs. American Flag × Race × Participant SDO		
	b (SE)	*t* (530)	*p*	*b* (SE)	*t* (530)	*p*
Likability	0.84 (0.34)	2.46	.014	-0.14 (0.30)	-0.45	.653
Comfort and Interest	0.74 (0.29)	2.51	.012	0.01 (0.26)	0.04	.967
Perceived Prejudice	-0.22 (0.19)	-1.12	.264	0.27 (0.17)	1.57	.116
Perceived Racism	-0.17 (0.18)	-0.95	.345	0.28 (0.15)	1.81	.070
Perceived Sexism	0.04 (0.18)	0.21	.831	0.29 (0.16)	1.79	.074
Race-based Classroom Mistreatment	-0.03 (0.29)	-0.12	.906	0.21 (0.25)	0.84	.400
Gender-based Classroom Mistreatment	0.12 (0.31)	0.39	.695	0.55 (0.27)	2.04	.042
Professor SDO	0.11 (0.24)	0.46	.647	-0.16 (0.21)	-0.78	.435
Professor Selection	0.55 (0.30)	1.81	.071	-0.39 (0.27)	-1.48	.140
**Testing for 2-way interactions between Condition and Participant SDO**						
Measures	No flag vs. Inclusive Flag × Participant SDO			No flag vs. American Flag × Participant SDO		
	b (SE)	*t* (530)	*p*	*b* (SE)	*t* (530)	*p*
Perceived Prejudice	0.65 (0.10)	6.74	< .001	-0.14 (0.09)	-1.59	.113
Perceived Racism	0.51 (0.09)	5.86	< .001	-0.04 (0.08)	-0.54	.587
Perceived Sexism	0.46 (0.09)	5.04	< .001	-0.05 (0.08)	-0.59	.558
Race-based Classroom Mistreatment	0.52 (0.14)	3.62	< .001	0.10 (0.13)	0.79	.430
Gender-based Classroom Mistreatment	0.53 (0.15)	3.50	< .001	0.01 (0.14)	0.09	.931
Professor SDO	0.42 (0.12)	3.55	< .001	-0.22 (0.10)	-2.08	.038

	**Simple of Effects of Flag Condition (No Flag vs. Inclusive Flag) at 1 Standard Deviation Above and Below the Mean on the Participant SDO Scale**											
Measures	White Participants						Black/Latine Participants					
	Low Participant SDO			High Participant SDO			Low Participant SDO			High Participant SDO		
	b (SE)	*t* (530)	*p*	*b* (SE)	*t* (530)	*p*	*b* (SE)	*t* (530)	*p*	*b* (SE)	*t* (530)	*p*

Likability	3.48 (0.29)	12.00	< .001	0.39 (0.36)	1.09	.278	2.85 (0.29)	9.79	< .001	1.25 (0.30)	4.12	< .001
Comfort and Interest	2.44 (0.25)	9.84	< .001	0.10 (0.31)	0.32	.747	2.08 (0.25)	8.33	< .001	1.03 (0.26)	3.96	< .001
Professor Selection	2.47 (0.26)	9.65	< .001	0.07 (0.32)	0.22	.827	2.10 (0.26)	8.14	< .001	0.65 (0.27)	2.44	.015
	**Simple of Effects of Flag Condition (No Flag vs. American Flag) at 1 Standard Deviation Above and Below the Mean on the Participant SDO Scale**											
Measures	White Participants						Black/Latine Participants					
	Low Participant SDO			High Participant SDO			Low Participant SDO			High Participant SDO		
	b (SE)	*t* (530)	*p*	*b* (SE)	*t* (530)	*p*	*b* (SE)	*t* (530)	*p*	*b* (SE)	*t* (530)	*p*
Likability	-0.08 (0.31)	-0.27	.788	0.42 (0.29)	1.47	.141	-0.79 (0.29)	-2.73	.007	-0.52 (0.28)	-1.85	.064
Comfort and Interest	-0.44 (0.26)	-1.68	.094	0.22 (0.25)	0.91	.362	-1.07 (0.25)	-4.30	< .001	-0.39 (0.24)	-1.60	.111
Professor Selection	-0.74 (0.27)	-2.76	.006	0.18 (0.25)	0.71	.479	-0.70 (0.26)	-2.72	.007	-0.46 (0.25)	-1.85	.064

Taken together, for likability, comfort and interest, and professor selection, race differences primarily only emerged at high levels of SDO. In Table S16, we also report no flag versus inclusive flag, and no flag versus American flag contrasts at high and low levels of participant SDO among White and Black/Latine participants for these three outcomes. As Figures 1 – 3 demonstrates, at low levels of SDO, White participants show similar significant condition effects as Black/Latine participants. However, at high levels of SDO, White participants no longer have significant condition effects, whereas Black/Latine participants still show these effects, which in part explains why race differences primarily emerge at high levels of SDO.

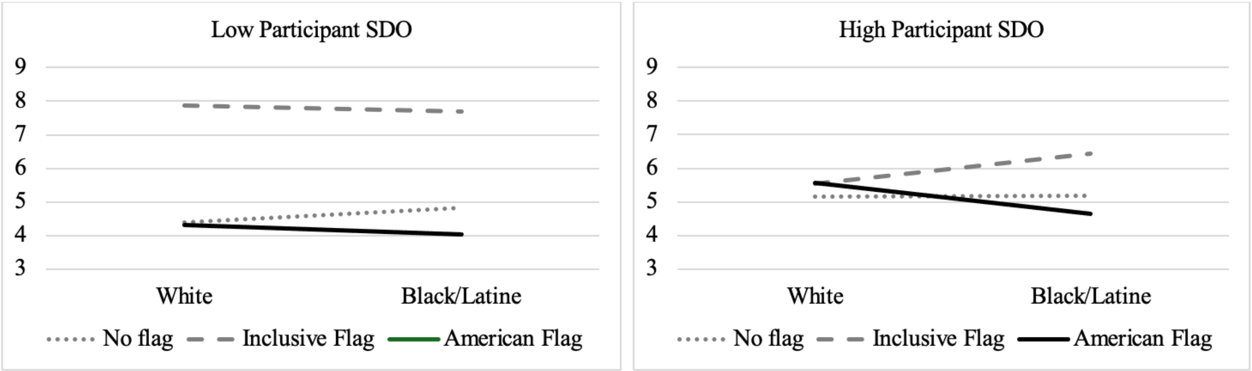

Figure 1. Simple Effects of Flag Condition for White and Black/Latine Participants at 1 Standard Deviation Below (left panel) and Above (right panel) the Mean on the Participant SDO Scale Predicting Likability.

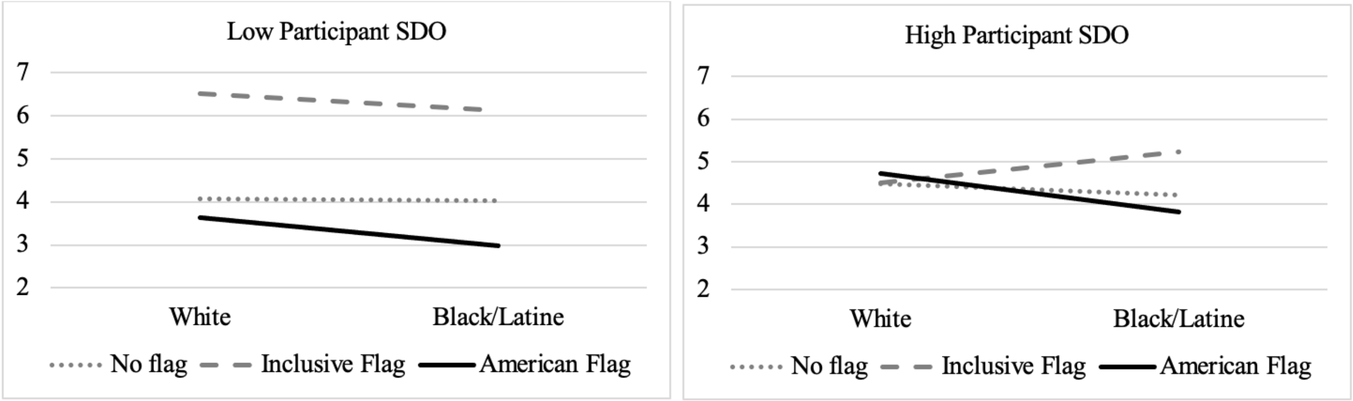

Figure 2. Simple Effects of Flag Condition for White and Black/Latine Participants at 1 Standard Deviation Below (left panel) and Above (right panel) the Mean on the Participant SDO Scale Predicting Comfort and Interest.

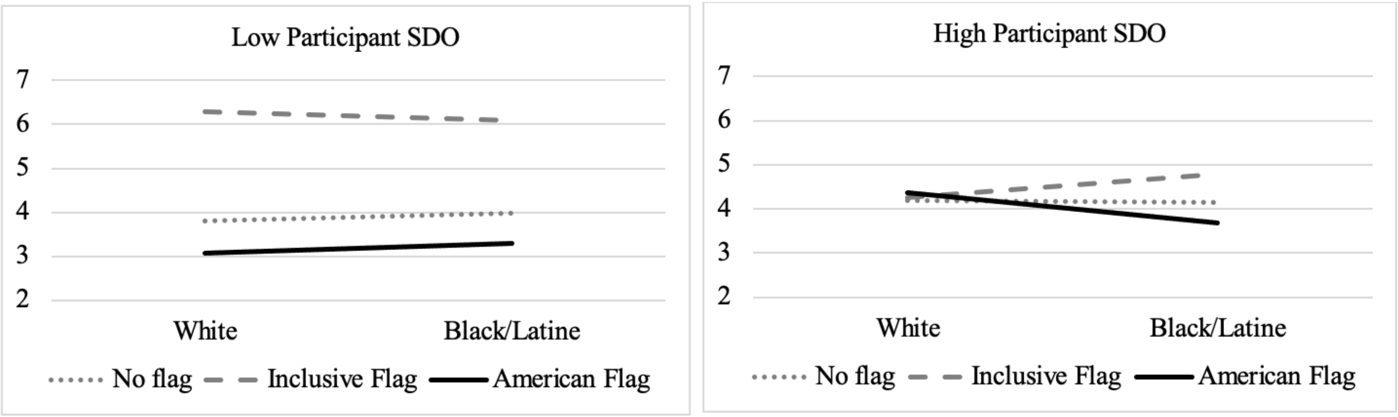

Figure 3. Simple Effects of Flag Condition for White and Black/Latine Participants at 1 Standard Deviation Below (left panel) and Above (right panel) the Mean on the Participant SDO Scale Predicting Professor Selection.

Additional Measures, Analyses, and Results for Study 2: Part 3

Moderation by Participant Gender

We found a significant main effect of participant gender for all outcome variables (*ps* < .042; see Table S18), except for professor SDO (*p* = .111). Women (vs. men) participants perceived the professor as more racist, more sexist, anticipated more race- and gender-based classroom mistreatment, and reported less comfort with, and a less likelihood of selecting, the professor (see Table S18). Across all outcome variables, we also found a significant main effect of flag condition (*ps* < .001; see Table 18). Compared to the no flag and inclusive flag conditions, the professor in the American flag condition was perceived as more racist (*ps* < .001), more sexist (*ps* < .001), higher in SDO (*ps* < .001), participants anticipated more race- and gender-based mistreatment (*ps* < .001), and reported less comfort and interest interacting with the professor (*ps* < .027; see Table S18). Participants also were more likely to select

the professor in the inclusive flag versus American flag condition ($p < .001$). For the no flag versus inclusive flag comparison, participants perceived the professor in the no flag condition as more racist, more sexist, higher in SDO, anticipated more race- and gender-based mistreatment, and reported less comfort with, and a less likelihood of selecting, the professor (all *ps* < .001; see Table S18). Lastly, we found significant participant gender by flag condition interactions on all outcome variables (all *ps* < .040; see Table S18). First for comfort and interest, in the no flag, *mean difference* = 0.52, SE = 0.21, p =.013, d = 0.38, and American flag, *mean difference* = 0.54, SE = 0.21, $p < .001$, d = 0.70 conditions, men reported more comfort and interest interacting with the professor than women participants. Conversely, in the inclusive flag condition, women reported more comfort and interest interacting with the professor than men participants, *mean difference* = 0.52, SE = 0.21, p = .010, d = 0.44. For perceived racism, in the no flag, *mean difference* = 0.37, SE = 0.12, p = .002, d = 0.50 and American flag, *mean difference* = 0.48, SE = 0.11, $p < .001$, d = 0.53 conditions, women participants reported more perceptions of racism than men. Whereas in the inclusive flag condition, men participants reported more perceptions of racism than women, *mean difference* = 0.41, SE = 0.12, $p < .001$, d = 0.60. For perceived sexism, in the no flag, *mean difference* = 0.36, SE = 0.12, p = .003, d = 0.44 and American flag *mean difference* = 0.55, SE = 0.12, $p < .001$, d = 0.63 conditions, women participants reported more perceptions of sexism than men participants. This effect was not found in the inclusive flag condition (p = .091). For anticipated race-based classroom mistreatment, in the no flag, *mean difference* = 0.61, SE = 0.20, p = .002, d = 0.49 and American flag, *mean difference* = 0.48, SE = 0.19, p = .012, d = 0.28 conditions, women participants reported more anticipated race-based classroom mistreatment than men participants. This effect was not found in the inclusive flag condition (p = .055). For anticipated gender-based classroom mistreatment, in the no flag, *mean difference* = 0.84, SE = 0.19, $p < .001$, d = 0.65 and

American flag, *mean difference* = 1.43, SE = 0.19, $p < .001$, $d = 0.91$ conditions, women participants reported more anticipated gender-based classroom mistreatment than men participants. This effect was not found in the inclusive flag condition ($p = .106$). For Professor SDO, in the American flag condition, women participants perceived the professor as higher in SDO than men participants, *mean difference* = 0.40, SE = 0.16, $p = .012$, $d = 0.30$. This effect was not found in the no flag ($p = .173$) and inclusive flag conditions ($p = .292$). For Professor selection, women participants were more willing to select the professor assigned to the inclusive flag than men participants, *mean difference* = 0.75, SE = 0.21, $p < .001$, $d = 0.52$. Conversely, men participants were more willing to select the professor assigned to the American flag than women participants, *mean difference* = 1.09, SE = 0.20, $p < .001$, $d = 0.74$. These effects were not found in the no flag condition ($p = .059$).

Table S17

Study 2 Means and Standard Deviations for Participant Gender and Flag Condition.

	Means and Standard Deviation Across Condition											
Measures	No Flag				Inclusive Flag				American Flag			
	Men (n = 86)		Women (n = 90)		Men (n = 78)		Women (n = 99)		Men (n = 93)		Women (n = 96)	
	M	SD	M	SD	M	SD	M	SD	M	SD	M	SD
Comfort and Interest	4.46	1.35	3.94	1.36	5.50	1.41	6.03	1.00	4.36	1.49	3.28	1.56
Perceived Racism	2.05	0.76	2.42	0.74	1.73	0.85	1.32	0.48	2.68	0.91	3.16	0.91
Perceived Sexism	2.10	0.85	2.47	0.81	1.68	0.86	1.47	0.57	2.55	0.82	3.10	0.93
Race-based Classroom Mistreatment	1.72	1.12	2.34	1.34	1.62	0.95	1.24	0.52	2.42	1.69	2.90	1.80
Gender-based Classroom Mistreatment	1.69	1.16	2.54	1.40	1.64	1.04	1.33	0.57	2.08	1.52	3.51	1.61
Professor SDO	3.19	0.92	3.42	1.01	1.88	0.99	1.71	0.73	3.94	1.28	4.34	1.38
Professor Selection	4.23	1.20	3.83	1.38	5.14	1.65	5.89	1.20	4.23	1.43	3.14	1.53

Measures	No Flag		Inclusive Flag		American Flag		Men		Women	
	M	SD	M	SD	M	SD	M	SD	M	SD
Comfort and Interest	4.19	1.38	5.80	1.22	3.81	1.61	4.74	1.50	4.45	1.78
Perceived Racism	2.24	0.77	1.50	0.70	2.92	0.94	2.18	0.93	2.29	1.06
Perceived Sexism	2.29	0.85	1.57	0.72	2.83	0.92	2.14	0.91	2.34	1.03

Race-based Classroom Mistreatment	2.04	1.27	1.40	0.76	2.66	1.76	1.94	1.36	2.15	1.49
Gender-based Classroom Mistreatment	2.13	1.35	1.47	0.82	2.81	1.72	1.82	1.28	2.45	1.56
Professor SDO	3.31	0.97	1.79	0.86	4.14	1.34	3.07	1.37	3.13	1.54
Professor Selection	4.03	1.31	5.56	1.46	3.67	1.57	4.51	1.49	4.31	1.81

Table S18

Study 2 Between-Subjects Results for Flag condition.

Measures	Flag Condition			Participant Gender Condition					Flag × Participant Gender		
	F (536)	*p*	η_p^2	*F* (536)	*p*	η_p^2	*MD (SE)*	*d*	*F* (536)	*p*	η_p^2
Comfort and Interest	101.04	<.001	.274	8.83	.003	.016	0.35 (0.12)	0.18	16.07	<.001	.057
Perceived Racism	143.30	<.001	.348	4.73	.030	.009	0.15 (0.07)	-0.11	17.21	<.001	.060
Perceived Sexism	107.51	<.001	.286	11.28	<.001	.021	0.24 (0.07)	-0.20	10.60	<.001	.038
Race-based Classroom Mistreatment	39.61	<.001	.129	4.35	.038	.008	0.24 (0.11)	-0.14	7.42	<.001	.027
Gender-based Classroom Mistreatment	48.49	<.001	.153	35.69	<.001	.062	0.65 (0.11)	-0.44	21.86	<.001	.075
Professor SDO	218.06	<.001	.449	2.55	.111	.005	0.15 (0.09)	-0.05	3.26	.039	.012
Professor Selection	86.15	<.001	.243	4.19	.041	.008	0.25 (0.12)	0.12	19.79	<.001	.069

Differences between Flag Conditions									
Measures	No Flag vs. Inclusive Flag			No Flag vs. American			Inclusive Flag vs. American		
	MD (*SE*)	*p*	*d*	*MD* (*SE*)	*p*	*d*	*MD* (*SE*)	*p*	*d*
Comfort and Interest	1.57 (0.15)	<.001	-1.23	0.38 (0.14)	.026	0.25	1.94 (0.14)	<.001	1.39
Perceived Racism	0.71 (0.08)	<.001	1.01	0.69 (0.08)	<.001	-0.80	1.40 (0.08)	<.001	-1.72
Perceived Sexism	0.71 (0.09)	<.001	0.92	0.54 (0.09)	<.001	-0.61	1.25 (0.09)	<.001	-1.53
Race-based Classroom Mistreatment	0.60 (0.14)	<.001	0.60	0.63 (0.14)	<.001	-0.41	1.23 (0.14)	<.001	-0.93
Gender-based Classroom Mistreatment	0.63 (0.14)	<.001	0.59	0.68 (0.13)	<.001	-0.44	1.31 (0.13)	<.001	-1.00
Professor SDO	1.51 (0.12)	<.001	1.66	0.83 (0.11)	<.001	-0.71	2.34 (0.11)	<.001	-2.09
Professor Selection	1.48 (0.15)	<.001	-1.10	0.35 (0.15)	.050	0.25	1.83 (0.15)	<.001	1.24

Note. MD = Mean Difference. SE = Standard Error.

Perceptions of Professor Race

We found a significant main effect of professor race for perceived racism (p = .008), perceived sexism (p < .001), professor selection (p = .021), but not for comfort and interest (p = .096) and professor SDO (p = .206; see Table S20). Participants who perceived the professor as White (vs. other race) also perceived the professor as more racist, sexist, and were less likely to select the professor. We also found a significant main effect of flag condition on all outcome variables (all *ps* < .001; see Table 20). Compared to participants in the no flag and American flag conditions, participants in the inclusive flag condition perceived the professor as less racist (*ps* < .001), less sexist (*ps* < .003), and lower in SDO (*ps* < .001). Moreover, participants reported more comfort and interest with, and a higher likelihood of selecting, the professor in the inclusive flag (vs. no flag and American flag; *ps* < .031; see Table S20) conditions. We did not find any significant differences between the no flag and American flag conditions (*ps* > .070; see Table S20). Lastly, we only found a significant flag condition by professor race interaction for professor selection (p = .017; see Table S20). In the no flag condition, participants who perceived the professor as White (vs. other race) were less likely to select the professor, *mean difference* = 0.62, *standard error* = 0.30, p = .040, d = 0.51. Similarly, in the American flag condition, participants who perceived the professor as White (vs. other race) were less likely to select the professor, *mean difference* = 1.61, *standard error* = 0.73, p = .028, d = 1.13. These effects were not significant in the inclusive flag condition (p = .343).

Table S19

Study 2 Means and Standard Deviations for Differences in Perceptions of Professor Race.

Measures	Means and Standard Deviation Across Condition											
	No Flag				Inclusive Flag				American Flag			
	Other Race (n = 27)		White (n = 149)		Other Race (n = 28)		White (n = 149)		Other Race (n = 4)		White (n = 185)	
	M	SD	M	SD	M	SD	M	SD	M	SD	M	SD
Comfort and Interest	4.42	1.19	4.15	1.41	5.79	1.24	5.80	1.22	4.92	2.06	3.79	1.60
Perceived Racism	2.12	0.77	2.26	0.77	1.40	0.56	1.52	0.72	1.94	0.94	2.94	0.93
Perceived Sexism	2.07	0.65	2.33	0.87	1.41	0.54	1.60	0.74	1.69	0.69	2.86	0.91
Professor SDO	3.35	0.76	3.30	1.00	1.97	0.85	1.75	0.86	3.09	1.13	4.16	1.34
Professor Selection	4.56	1.12	3.93	1.32	5.32	1.47	5.60	1.46	5.25	1.26	3.64	1.57

Measures	No Flag		Inclusive Flag		American Flag		Other Race		White	
	M	SD	M	SD	M	SD	M	SD	M	SD
Comfort and Interest	4.19	1.38	5.80	1.22	3.81	1.61	5.10	1.42	4.52	1.67
Perceived Racism	2.24	0.77	1.50	0.70	2.92	0.94	1.77	0.76	2.29	1.01
Perceived Sexism	2.29	0.85	1.57	0.72	2.83	0.92	1.73	0.67	2.30	1.00
Professor SDO	3.31	0.97	1.79	0.86	4.14	1.34	2.68	1.06	3.15	1.49
Professor Selection	4.03	1.31	5.56	1.46	3.67	1.57	4.97	1.34	4.34	1.69

Table S20

Study 2 Between-Subjects Results for Flag condition.

Measures	Flag Condition			Professor Race Condition					Flag × Professor Race		
	F (553)	p	η_p^2	F (553)	p	η_p^2	MD (SE)	d	F (553)	p	η_p^2
Comfort and Interest	27.94	<.001	.094	2.78	.096	.005	0.46 (0.28)	0.37	1.13	.324	.004
Perceived Racism	23.22	<.001	.080	7.16	.008	.013	0.42 (0.16)	-0.59	2.14	.118	.008
Perceived Sexism	18.41	<.001	.064	11.04	<.001	.020	0.54 (0.16)	-0.67	2.41	.091	.009
Professor SDO	49.20	<.001	.155	1.60	.206	.003	0.27 (0.21)	0.37	2.37	.094	.009
Professor Selection	17.11	<.001	.060	5.34	.021	.010	0.65 (0.28)	0.41	4.09	.017	.015

Measures	Differences between Flag Conditions								
	No Flag vs. Inclusive Flag			No Flag vs. American			Inclusive Flag vs. American		
	MD (SE)	p	d	MD (SE)	p	d	MD (SE)	p	d
Comfort and Interest	1.51 (0.21)	<.001	-1.23	0.07 (0.39)	1.00	0.25	1.44 (0.39)	<.001	1.39

Perceived Racism	0.73 (0.12)	<.001	1.01	0.25 (0.22)	.771	-0.80	0.98 (0.22)	<.001	-1.72
Perceived Sexism	0.70 (0.12)	<.001	0.92	0.07 (0.23)	1.00	-0.61	0.77 (0.23)	.002	-1.53
Professor SDO	1.47 (0.16)	<.001	1.66	0.30 (0.30)	.917	-0.71	1.77 (0.30)	<.001	-2.09
Professor Selection	1.22 (0.21)	<.001	-1.10	0.20 (0.40)	1.00	0.25	1.02 (0.39)	.030	1.24

Note. MD = Mean Difference. SE = Standard Error.

Perceptions of Professor Gender

We only found a significant main effect of professor gender for comfort and interest ($p = .027$; see Table S22). Participants who perceived the professor as a woman (vs. a man) reported more comfort and interest interacting with the professor. We also found a significant main effect of flag condition across all outcome variables (all *ps* < .001; see Table S22). Compared to the no flag and American flag conditions, participants in the inclusive flag condition perceived the professor as less racist (*ps* < .015), less sexist (*ps* < .018), and lower in SDO (*ps* < .001; see Table S22). Participants also reported more comfort and interest interacting with, and a higher likelihood of selecting, the professor in the inclusive flag versus no flag condition (*ps* < .001); however, this effect was not significant when comparing the inclusive flag versus American flag conditions (*ps* > .054; see Table S22). Moreover, we did not find any significant differences between the no flag and American flag conditions (all *ps* > .999; see Table S22). Lastly, we found significant flag condition by professor gender interactions across all outcome variables (all *ps* < .036; see Table S22).

First for comfort and interest, compared to the no flag, *mean difference* = 1.71, *standard error* = 0.16, $p < .001$, $d = -1.33$ and American flag, *mean difference* = 2.07, *standard error* = 0.16, $p < .001$, $d = 0.24$ conditions, participants who perceived the professor as a man reported more comfort and interest interacting with the professor in the inclusive flag condition. We did not find any

significant differences between the no flag and American flag conditions on comfort and interest among participants who perceived the professor as a man ($p = .058$). Moreover, we did not find any significant differences between the flag conditions on comfort and interest for participants who perceived the professor as a woman ($ps > .095$). For perceived racism, compared to the no flag, *mean difference* = 0.68, *standard error* = 0.87, $p < .001$, $d = 0.80$ and inclusive flag, *mean difference* = 1.53, *standard error* = 0.89, $p < .001$, $d = 1.98$ conditions, participants who perceived the professor as a man reported more perceptions of racism in the American flag condition. These participants also reported more perceptions of racism in the no flag versus no inclusive flag condition, *mean difference* = 0.84, *standard error* = 0.09, $p < .001$, $d = 1.24$. We did not find any significant differences between the flag conditions on perceived racism for participants who perceived the professor as a woman ($ps > .999$). For perceived sexism, compared to the no flag, *mean difference* = 0.52, *standard error* = 0.09, $p < .001$, $d = 0.58$ and inclusive flag, *mean difference* = 1.34, *standard error* = 0.09, $p < .001$, $d = 1.71$ conditions, participants who perceived the professor as a man reported more perceptions of sexism in the American flag condition. These participants also reported more perceptions of sexism in the no flag versus inclusive flag condition, *mean difference* = 0.82, *standard error* = 0.10, $p < .001$, $d = 1.10$. We did not find any significant differences between the flag conditions on perceived sexism for participants who perceived the professor as a woman ($ps > .999$). For professor SDO, compared to the no flag, *mean difference* = 0.84, *standard error* = 0.12, $p < .001$, $d = 0.72$ and inclusive flag, *mean difference* = 2.49, *standard error* = 0.12, $p < .001$, $d = 2.27$ conditions, participants who perceived the professor as a man reported more perceptions of SDO in the American flag condition. These participants also reported more perceptions of SDO in the no flag versus inclusive flag condition, *mean difference* = 1.65, *standard error* = 0.12, $p < .001$, $d = 1.85$. Moreover, compared to the inclusive flag condition, participants who perceived the

professor as a woman reported more perceptions of SDO in the no flag condition, *mean difference* = 0.82, *standard error* = 0.32, *p* = .029, *d* = 0.89. We did not find any significant differences between the American flag versus no flag and inclusive flag conditions on professor SDO for participants who perceived the professor as a woman (*ps* > .999). Lastly, for professor selection, compared to the no flag, *mean difference* = 1.66, *standard error* = 0.17, *p* < .001, *d* = 1.24 and American flag, *mean difference* = 2.01, *standard error* = 0.16, *p* < .001, *d* = 1.37 conditions, participants who perceived the professor as a man were more likely to select the professor in the inclusive flag condition. We did not find a significant difference between the no flag versus American flag conditions on professor selection among participants who perceived the professor as a man (*p* = .079). Moreover, we did not find any significant differences between the flag conditions on professor selection for participants who perceived the professor as a woman (*ps* > .168).

Table S21

Study 2 Means and Standard Deviations for Differences in Perceptions of Professor Gender.

	Means and Standard Deviation Across Condition											
Measures	No Flag				Inclusive Flag				American Flag			
	Woman (*n* = 11)		Man (*n* = 173)		Woman (*n* = 7)		Man (*n* = 178)		Woman (*n* = 7)		Man (*n* = 183)	
	M	SD	M	SD	M	SD	M	SD	M	SD	M	SD
Comfort and Interest	4.14	1.40	4.65	1.02	5.85	1.15	5.54	1.51	3.78	1.60	5.67	2.03
Perceived Racism	2.26	0.77	2.04	0.72	1.42	0.57	1.88	1.03	2.94	0.93	1.75	1.09
Perceived Sexism	2.33	0.86	1.94	0.69	1.51	0.62	1.84	1.02	2.85	0.91	1.92	0.95
Professor SDO	3.33	0.98	3.09	0.85	1.68	0.79	2.27	1.00	4.17	1.33	2.50	1.50
Professor Selection	4.00	1.33	4.28	1.13	5.66	1.36	5.09	1.80	3.65	1.56	5.00	2.00

Measures	No Flag		Inclusive Flag		American Flag		Other Gender		Man	
	M	SD	M	SD	M	SD	M	SD	M	SD
Comfort and Interest	4.19	1.38	5.80	1.22	3.81	1.61	4.51	1.66	5.25	1.43
Perceived Racism	2.24	0.77	1.50	0.70	2.92	0.94	2.27	1.00	1.92	0.92
Perceived Sexism	2.29	0.85	1.57	0.72	2.83	0.92	2.28	0.98	1.88	0.90
Professor SDO	3.31	0.97	1.79	0.86	4.14	1.34	3.16	1.49	2.56	1.03
Professor Selection	4.03	1.31	5.56	1.46	3.67	1.57	4.36	1.67	4.81	1.63

Table S22

Study 2 Between-Subjects Results for Flag condition.

Measures	Flag Condition			Professor Gender Condition					Flag × Professor Gender		
	F (553)	*p*	η_p^2	*F* (553)	*p*	η_p^2	*MD (SE)*	*d*	*F* (553)	*p*	η_p^2
Comfort and Interest	17.66	<.001	.062	4.95	.027	.009	0.69 (0.31)	-0.47	4.14	.016	.015
Perceived Racism	10.16	<.001	.037	3.23	.073	.006	0.32 (0.18)	0.36	7.73	<.001	.028
Perceived Sexism	8.51	<.001	.031	3.16	.076	.006	0.33 (0.18)	0.42	5.80	.003	.021
Professor SDO	30.36	<.001	.102	3.45	.064	.006	0.44 (0.24)	0.47	7.52	<.001	.027
Professor Selection	15.51	<.001	.055	1.22	.270	.002	0.35 (0.32)	-0.27	3.38	.035	.012

Differences between Flag Conditions									
Measures	No Flag vs. Inclusive Flag			No Flag vs. American			Inclusive Flag vs. American		
	MD (*SE*)	*p*	*d*	*MD* (*SE*)	*p*	*d*	*MD* (*SE*)	*p*	*d*
Comfort and Interest	1.30 (0.22)	<.001	-1.23	0.33 (0.45)	1.00	0.25	0.97 (0.43)	.076	1.39
Perceived Racism	0.51 (0.13)	<.001	1.01	0.20 (0.25)	1.00	-0.80	0.70 (0.25)	.014	-1.72
Perceived Sexism	0.46 (0.13)	.001	0.92	0.24 (0.26)	1.00	-0.61	0.71 (0.25)	.017	-1.53
Professor SDO	1.24 (0.17)	<.001	1.66	0.12 (0.34)	1.00	-0.71	1.36 (0.33)	<.001	-2.09
Professor Selection	1.24 (0.23)	<.001	-1.10	0.19 (0.46)	1.00	0.25	1.05 (0.45)	.055	1.24

Note. MD = Mean Difference. SE = Standard Error.

Additional Measures, Analyses, and Results for Study 2: Part 4

For Study 2, we also examined whether there were any differences between the Black and Latine participants. The descriptives and correlations for all supplemental outcome variables in Study 2 are available in Table S9.

Table S23

Supplemental Measure information and statistics, reliabilities, and correlations for Study 2.

Measure	M (SD)	1	2	3	4	5	6	7
1. Likability	5.54 (1.98)	(NA)						
2. Perceived Similarity	2.97 (1.13)	.81***	(0.95)					
3. Positive Affect	2.23 (1.05)	.61***	.57***	(0.95)				
4. Negative Affect	1.41 (0.64)	-.42***	-.41***	.03	(0.93)			
5. Perceived Prejudice	3.04 (1.17)	-.81***	-.79***	-.58***	.52***	(0.96)		
6. Race-based Classroom Mistreatment	2.05 (1.44)	-.48***	-.46***	-.25***	.43***	.59***	(0.95)	
7. Gender-based Classroom Mistreatment	2.15 (1.46)	-.50***	-.49***	-.27***	.49***	.63***	.76***	(0.96)

Note. The reliability for each scale is shown on the diagonal. $^{*}p < .05$, $^{**}p < .01$, $^{***}p < .001$

Results

To examine whether participant race influenced perceptions across flag conditions, we conducted separate 2 (participant race: Black vs. Latine) x 3 (flag condition: no flag vs. inclusive flag vs. gender condition) between-subjects ANOVA for each outcome. See Table S24 for means and standard deviations, and Table S25 for contrasts between conditions and full ANOVA results. Bonferroni corrections were applied to account for multiple comparisons.

Table S24

Supplemental Study 2 Means and Standard Deviation for Black vs. Latine participants.

	Means and Standard Deviation Across Condition											
Measures	No Flag				Inclusive Flag				American Flag			
	Latine (*n* = 52)		Black (*n* = 40)		Latine (*n* = 50)		Black (*n* = 44)		Latine (*n* = 58)		Black (*n* = 48)	
	M	SD	M	SD	M	SD	M	SD	M	SD	M	SD
Likability	4.88	1.56	5.18	1.57	7.22	1.50	7.05	1.73	4.45	1.82	4.25	1.44

Comfort and Interest	4.03	1.31	4.26	1.44	5.82	0.97	5.63	1.27	3.48	1.45	3.32	1.57
Perceived Similarity	2.64	0.83	2.61	0.81	3.76	0.84	3.73	1.08	2.25	0.91	2.31	1.13
Positive Affect	1.80	0.76	1.97	0.87	2.81	0.93	3.00	1.19	1.84	0.92	2.06	1.08
Negative Affect	1.55	0.75	1.43	0.74	1.11	0.30	1.23	0.57	1.72	0.79	1.64	0.72
Perceived Prejudice	3.47	0.84	3.17	0.89	2.19	0.94	2.03	0.83	3.82	0.93	3.94	0.86
Perceived Racism	2.26	0.79	2.27	0.89	1.52	0.72	1.43	0.57	3.00	0.93	3.16	0.96
Perceived Sexism	2.25	0.80	2.29	0.93	1.63	0.74	1.48	0.66	2.85	0.82	2.92	0.90
Race-based Classroom Mistreatment	2.01	1.22	2.38	1.45	1.50	0.87	1.33	0.62	2.93	1.79	3.65	1.74
Gender-based Classroom Mistreatment	1.87	1.17	2.31	1.53	1.53	1.02	1.45	0.84	2.71	1.73	3.00	1.65
Professor SDO	3.50	0.99	3.24	1.00	2.01	1.02	1.94	0.90	4.18	1.40	4.11	1.43
Professor Selection	3.92	1.23	4.25	1.39	5.62	1.16	5.41	1.68	3.50	1.49	3.48	1.53

Measures	No Flag		Inclusive Flag		American Flag		Latine		Black	
	M	SD	M	SD	M	SD	M	SD	M	SD
Likability	5.01	1.56	7.14	1.60	4.36	1.65	5.46	2.03	5.46	1.96
Comfort and Interest	4.13	1.37	5.73	1.12	3.41	1.50	4.39	1.61	4.38	1.72
Perceived Similarity	2.63	0.82	3.74	0.95	2.28	1.01	2.85	1.07	2.87	1.19
Positive Affect	1.87	0.81	2.90	1.06	1.94	1.00	2.13	0.98	2.35	1.15
Negative Affect	1.50	0.74	1.16	0.45	1.68	0.76	1.47	0.71	1.44	0.70
Perceived Prejudice	3.34	0.87	2.12	0.89	3.88	0.90	3.20	1.14	3.07	1.17
Perceived Racism	2.27	0.83	1.47	0.65	3.07	0.95	2.30	1.02	2.31	1.10
Perceived Sexism	2.27	0.86	1.56	0.70	2.88	0.85	2.27	0.93	2.25	1.03
Race-based Classroom Mistreatment	2.17	1.33	1.42	0.77	3.25	1.80	2.19	1.49	2.49	1.67
Gender-based Classroom Mistreatment	2.06	1.35	1.49	0.93	2.84	1.69	2.07	1.44	2.27	1.53
Professor SDO	3.39	0.99	1.98	0.96	4.15	1.41	3.28	1.47	3.12	1.46
Professor Selection	4..07	1.31	5.52	1.42	3.49	1.50	4.30	1.59	4.36	1.73

Table S25

Supplemental Study 2 Between-Subjects ANOVA Results for Black vs. Latine participants.

Measures	Flag Condition			Participant Race Condition					Flag × Participant Race		
	F (286)	*p*	η_p^2	*F* (286)	*p*	η_p^2	*MD (SE)*	*d*	*F* (286)	*p*	η_p^2
Likability	78.60	<.001	.355	0.02	.885	<.001	0.03 (0.19)	0.00	0.68	.506	.005
Comfort and Interest	75.65	<.001	.346	0.05	.822	<.001	0.04 (0.16)	0.01	0.69	.501	.005
Perceived Similarity	64.07	<.001	.309	0.00	.984	<.001	0.00 (0.98)	-0.02	0.07	.937	<.001
Positive Affect	33.32	<.001	.189	2.77	.097	.010	0.19 (0.11)	-0.20	0.02	.983	<.001
Negative Affect	14.70	<.001	.093	0.09	.763	<.001	0.02 (0.08)	0.05	0.82	.441	.006
Perceived Prejudice	102.05	<.001	.416	1.18	.279	.004	0.11 (0.10)	0.11	1.41	.245	.010

Perceived Racism	93.92	<.001	.396	0.06	.806	<.001	0.02, 0.10	-0.01	0.56	.572	.004
Perceived Sexism	66.70	<.001	.318	0.02	.886	<.001	0.01 (0.10)	0.03	0.46	.634	.003
Race-based Classroom Mistreatment	47.00	<.001	.247	3.46	.064	.012	0.30 (0.16)	-0.19	2.62	.075	.018
Gender-based Classroom Mistreatment	24.59	<.001	.147	1.79	.182	.006	0.22 (0.16)	-0.14	0.92	.402	.006
Professor SDO	88.33	<.001	.382	0.96	.327	.003	0.13 (0.14)	0.11	0.20	.821	.001
Professor Selection	52.36	<.001	.268	0.04	.850	<.001	0.03 (0.17)	-0.04	0.85	.429	.006

Differences between Message Conditions									
Measures	No Flag vs. Inclusive Flag			No Flag vs. American			Inclusive Flag vs. American		
	MD (*SE*)	*p*	*d*	*MD* (*SE*)	*p*	*d*	*MD* (*SE*)	*p*	*d*
Likability	2.10 (0.24)	<.001	-1.35	0.68 (0.23)	.011	0.40	2.78 (0.23)	<.001	1.71
Comfort and Interest	1.59 (0.20)	<.001	-1.29	0.74 (0.19)	<.001	0.50	2.32 (0.19)	<.001	1.75
Perceived Similarity	1.12 (0.14)	<.001	-1.26	0.35 (0.14)	.033	0.38	1.46 (0.13)	<.001	1.49
Positive Affect	1.03 (0.14)	<.001	-1.10	0.07 (0.14)	1.00	-0.08	0.95 (0.14)	<.001	0.93
Negative Affect	0.32 (0.10)	.004	0.54	0.19 (0.10)	.144	-0.25	0.51(0.10)	<.001	-0.84
Perceived Prejudice	1.21 (0.13)	<.001	1.39	0.56 (0.13)	<.001	-0.61	1.77 (0.13)	<.001	-1.97
Perceived Racism	0.80 (0.12)	<.001	1.06	0.81 (0.12)	<.001	-0.90	1.61 (0.12)	<.001	-1.97
Perceived Sexism	0.72 (0.12)	<.001	0.90	0.62 (0.12)	<.001	-0.72	1.33 (0.12)	<.001	-1.70
Race-based Classroom Mistreatment	0.78 (0.20)	<.001	0.69	1.10 (0.20)	<.001	0.69	1.88 (0.20)	<.001	1.33
Gender-based Classroom Mistreatment	0.60 (0.20)	.011	0.49	0.77 (0.20)	<.001	-0.51	1.36 (0.20)	<.001	-0.99
Professor SDO	1.40 (0.17)	<.001	1.44	0.77 (0.17)	<.001	-0.62	2.17 (0.17)	<.001	-1.80
Professor Selection	1.43(0.21)	<.001	-1.06	0.60 (0.20)	.011	0.41	2.03 (0.20)	<.001	1.39

Across all outcomes, there was no significant main effect of participant race (all *ps* > .063) nor any significant participant race by flag condition interactions (all *ps* > .074; see Table S25). However, we found a significant main effect of flag condition across all outcome variables (all *ps* < .001; see Table S25). Compared to the no flag and inclusive flag conditions, participants in the professor in

the American flag condition reported less likability (*ps* < .012), less comfort and interest (*ps* < .001), less similarity (*ps* < .034), less likelihood of selecting the professor (*ps* < .012), and a more perceptions of prejudice, racism, sexism, race- and gender-based classroom mistreatment, and SDO (all *ps* < .001; see Table S25). We did not find any significant differences between the no flag and inclusive flag conditions for positive and negative affect (*ps* > .999; see Table S25). Moreover, compared to the no flag condition, participants in the inclusive flag condition reported more likability, more comfort and interest, more perceived similarity, more positive affect, and were more willing to select the professor (all *ps* < .001; see Table S25). Additionally, participants in the no flag versus inclusive flag condition reported more negative affect (*p* = .004) perceived prejudice (*p* < .001), more racism (*p* < .001), more sexism (*p* < .001), more race-based classroom mistreatment (*p* < .001), more gender-based classroom mistreatment (*p* = .011), and more SDO (*p* < .001; see Table S25).

Moderation by Participant Social Dominance Orientation (SDO)

To examine whether Participant SDO moderated the race and flag condition, we ran a regression model with condition dummy and the no flag condition as the reference group, race (0 = Latine participants and 1 = Black participants), Participant SDO, and all interactions. We did not find any significant three-way interactions between the no flag versus inclusive flag contrast (*ps* > .216), race, and participant SDO predicting any of the outcome variables (see Table S26). We also did not find any significant three-way interactions between the no flag versus American flag contrast (*ps* > .189), race, and participant SDO predicting any of the outcome variables (see Table S26). However, for all outcome measures, we examined two-way interactions between condition dummy coded (with no flag condition as the reference group), and participant SDO (all two-way interactions are reported in Table S26).

Table S26

Interactions with Participant SDO, Participant Race (Black vs. Latine), and Flag Conditions from Supplemental Study 2.

Testing for 3-way Interactions with Flag Condition, Participant Race, and Participant SDO						
Measures	No flag vs. Inclusive Flag× Race × Participant SDO			No flag vs. American Flag × Race × Participant SDO		
	b (SE)	*t* (280)	*p*	*b* (SE)	*t* (280)	*p*
Likability	-0.10 (0.50)	-0.20	.844	0.24 (0.48)	0.50	.616
Comfort and Interest	-0.16 (0.42)	-0.39	.697	0.36 (0.40)	0.89	.372
Perceived Similarity	-0.01 (0.28)	-0.04	.967	0.17 (0.27)	0.61	.541
Positive Affect	-0.22 (0.30)	-0.72	.470	0.06 (0.29)	0.22	.826
Negative Affect	0.26 (0.21)	1.24	.217	0.09 (0.20)	0.44	.662
Perceived Prejudice	-0.10 (0.28)	-0.35	.726	-0.35 (0.26)	-1.31	.190
Perceived Racism	-0.03 (0.26)	-0.10	.918	0.08 (0.25)	0.31	.758
Perceived Sexism	-0.27 (0.26)	-1.07	.286	-0.21 (0.25)	-0.85	.398
Race-based Classroom Mistreatment	-0.12 (0.45)	-0.28	.782	-0.01 (0.43)	-0.02	.982
Gender-based Classroom Mistreatment	-0.20 (0.44)	-0.46	.646	-0.23 (0.43)	-0.55	.583
Professor SDO	0.04 (0.36)	0.12	.905	0.03 (0.34)	0.08	.937
Professor Selection	-0.36 (0.44)	-0.82	.414	0.31 (0.42)	0.75	.456
Testing for 2-way interactions between Condition and Participant SDO						
Measures	No flag vs. Inclusive Flag × Participant SDO			No flag vs. American Flag × Participant SDO		
	b (SE)	*t* (280)	*p*	*b* (SE)	*t* (280)	*p*
Likability	-0.92 (0.25)	-3.75	< .001	0.16 (0.24)	0.68	.498
Comfort and Interest	-0.62 (0.20)	-3.06	.002	0.36 (0.20)	1.84	.066
Perceived Similarity	-0.58 (0.14)	-4.21	< .001	0.25 (0.13)	1.86	.064
Positive Affect	-0.28 (0.15)	-1.90	.059	0.20 (0.14)	1.40	.163
Negative Affect	0.18 (0.10)	1.72	.086	0.20 (0.10)	1.99	.048
Perceived Prejudice	0.56 (0.13)	4.20	< .001	0.01 (0.13)	0.06	.956
Perceived Racism	0.45 (0.13)	3.53	< .001	0.11 (0.12)	0.87	.386
Perceived Sexism	0.46 (0.13)	3.64	< .001	0.07 (0.12)	0.57	.567
Race-based Classroom Mistreatment	0.48 (0.22)	2.23	.027	0.18 (0.21)	0.85	.399
Gender-based Classroom Mistreatment	0.55 (0.22)	2.51	.013	0.23 (0.21)	1.10	.273
Professor SDO	0.49 (0.17)	2.81	.005	-0.27 (0.17)	-1.63	.104
Professor Selection	-0.86 (0.21)	-4.02	< .001	0.09 (0.21)	0.46	.645

When interactions were significant, we examined the effect of condition at one standard deviation below and above the mean on participant SDO (see Table S27). At both low (*ps* < .001) and high (*ps* < .005) levels of SDO, participants perceived the professor in the inclusive flag versus no flag condition as more likeable, and reported more comfort and interest, more perceived similarity, and a higher likelihood of selecting the professor; however, these effects was larger for participants low in SDO (see Table S27). At both low (*ps* < .001) and high (*ps* < .034) levels of SDO, participants perceived the professor in the no flag versus inclusive flag condition as more prejudiced, more racist, more sexist, higher in SDO, and this professor elicited more negative affect; however, these effects were larger for participants low in SDO (see Table S27). At low levels of SDO, participants anticipated more race- and gender-based classroom mistreatment from the professor in the no flag versus inclusive flag condition (*ps* < .001); however, these effects were not significant for participants at high levels of SDO (*ps* > .435; see Table S27). Although not significant, at both low (*p* = .057) and high (*p* = .065) levels of SDO, the professor in the American flag versus no flag condition elicited more negative affect; however, this effect was larger for participants low in SDO (see Table S27).

Table S27

Simple Effects of Flag Condition at 1 Standard Deviation Below and Above the Mean on the Participant SDO Scale from Supplemental Study 2.

Measures	No Flag (0) versus Inclusive Flag (1)						No Flag (0) versus American Flag (1)					
	Low Participant SDO			High Participant SDO			Low Participant SDO			High Participant SDO		
	b (SE)	*t* (536)	*p*	*b* (SE)	*t* (536)	*p*	*b* (SE)	*t* (536)	*p*	*b* (SE)	*t* (536)	*p*
Likability	3.15 (0.21)	15.15	< .001	0.94 (0.23)	4.06	< .001	-0.47 (0.21)	-2.24	.026	-0.06 (0.20)	-0.30	.761
Comfort and Interest	2.24 (0.18)	12.53	< .001	0.68 (0.20)	3.41	< .001	-0.82 (0.18)	-4.50	< .001	-0.09 (0.18)	-0.53	.596
Perceived Similarity	1.71 (0.12)	14.85	< .001	0.37 (0.13)	2.88	.004	-0.50 (0.12)	-4.29	< .001	-0.02 (0.11)	-0.20	.842
Negative Affect	-0.39 (0.08)	-4.72	< .001	-0.23 (0.09)	-2.54	.011	0.16 (0.08)	1.91	.057	0.15 (0.08)	1.85	.065

Perceived Prejudice	-1.70 (0.12)	-14.78	< .001	0.58 (0.12)	4.91	< .001	-0.62 (0.13)	-4.69	< .001	0.30 (0.11)	2.65	.008
Perceived Racism	-1.08 (0.11)	-10.14	< .001	-0.26 (0.12)	-2.18	.030	0.77 (0.11)	7.16	< .001	0.65 (0.10)	6.20	< .001
Perceived Sexism	-1.05 (0.11)	-9.56	< .001	-0.26 (0.12)	-2.13	.033	0.62 (0.11)	5.57	< .001	0.51 (0.11)	4.72	< .001
Race-based Classroom Mistreatment	-0.96 (0.18)	-5.29	< .001	-0.16 (0.20)	-0.78	.436	0.62 (0.18)	3.35	< .001	0.66 (0.18)	3.68	< .001
Gender-based Classroom Mistreatment	-1.04 (0.18)	-5.69	< .001	-0.12 (0.21)	-0.58	.562	0.72 (0.19)	3.85	< .001	0.69 (0.18)	3.85	< .001
Professor SDO	-1.81 (0.14)	-12.79	< .001	-1.00 (0.16)	-6.29	< .001	1.03 (0.14)	7.11	< .001	0.66 (0.14)	4.74	< .001
Professor Selection	2.27 (0.18)	12.43	< .001	0.44 (0.20)	2.16	.031	-0.72 (0.19)	-3.86	< .001	-0.12 (0.18)	-0.69	.487

Moderation by Participant Political Orientation

We next examined whether participants reported political orientation (1 = *Extremely Liberal* to 7 = *Extremely Conservative*) moderated the race and flag condition, we ran a regression model with condition dummy and the no flag condition as the reference group, race (0 = Latine participants and 1 = Black participants), Political Orientation, and all interactions. We did not find any significant three-way interactions between the no flag versus inclusive flag contrast (*ps* > .230), race, and participant SDO predicting any of the outcome variables (see Table S13). We also did not find any significant three-way interactions between the no flag versus American flag contrast (*ps* > .139), race, and political orientation predicting any of the outcome variables (see Table S28). However, for all outcome measures, we examined two-way interactions between condition dummy coded (with no flag condition as the reference group), and political orientation (all two-way interactions are reported in Table S28).

Table S28

Interactions with Political Orientation, Participant Race (Black vs. Latine), and Flag Conditions from Supplemental Study 2.

Testing for 3-way Interactions with Flag Condition, Participant Race, and Political Orientation		
Measures	No flag vs. Inclusive Flag× Race × Political Orientation	No flag vs. American Flag × Race × Political Orientation

Measures	b (SE)	t (280)	p	b (SE)	t (280)	p
Likability	-0.22 (0.29)	-0.74	.458	-0.25 (0.30)	-0.83	.407
Comfort and Interest	-0.29 (0.25)	-1.20	.231	-0.24 (0.25)	-0.96	.340
Perceived Similarity	-0.11 (0.17)	-0.67	.505	0.04 (0.17)	0.21	.831
Positive Affect	0.09 (0.18)	0.48	.632	-0.00 (0.18)	-0.02	.984
Negative Affect	0.12 (0.13)	0.90	.367	0.20 (0.13)	1.48	.140
Perceived Prejudice	0.09 (0.16)	0.54	.591	0.10 (0.17)	0.58	.565
Perceived Racism	-0.05 (0.15)	-0.33	.740	0.12 (0.16)	0.75	.451
Perceived Sexism	-0.15 (0.15)	-0.99	.325	0.11 (0.15)	0.66	.511
Race-based Classroom Mistreatment	0.02 (0.26)	0.06	.952	0.30 (0.26)	1.13	.258
Gender-based Classroom Mistreatment	-0.06 (0.26)	-0.23	.816	0.11 (0.27)	0.43	.669
Professor SDO	-0.18 (0.22)	-0.81	.416	0.17 (0.22)	0.77	.441
Professor Selection	-0.19 (0.26)	-0.75	.455	-0.16 (0.27)	-0.61	.541
Testing for 2-way interactions between Condition and Political Orientation						
Measures	No flag vs. Inclusive Flag × Political Orientation			No flag vs. American Flag × Political Orientation		
	b (SE)	*t* (280)	*p*	*b* (SE)	*t* (280)	*p*
Likability	-0.48 (0.15)	-3.29	.001	0.33 (0.15)	2.21	.028
Comfort and Interest	-0.32 (0.12)	-2.59	.010	0.37 (0.13)	2.99	.003
Perceived Similarity	-0.38 (0.08)	-4.66	< .001	0.26 (0.08)	3.13	.002
Positive Affect	-0.00 (0.09)	-0.03	.976	0.29 (0.09)	3.19	.002
Negative Affect	0.10 (0.06)	1.53	.128	0.04 (0.07)	0.55	.580
Perceived Prejudice	0.30 (0.08)	3.70	< .001	-0.07 (0.08)	-0.88	.382
Perceived Racism	0.25 (0.08)	3.29	.001	-0.03 (0.08)	-0.39	.699
Perceived Sexism	0.25 (0.08)	3.32	.001	0.06 (0.08)	0.73	.466
Race-based Classroom Mistreatment	0.18 (0.13)	1.38	.170	-0.21 (0.13)	-1.58	.116
Gender-based Classroom Mistreatment	0.19 (0.13)	1.43	.155	-0.16 (0.14)	-1.21	.228
Professor SDO	0.19 (0.11)	1.79	.075	-0.39 (0.11)	-3.51	< .001
Professor Selection	-0.36 (0.13)	-2.79	.006	0.44 (0.13)	3.34	.001

When interactions were significant, we examined the effect of condition at one standard deviation below and above the mean on political orientation (see Table S29). At one standard deviation below (*ps* < .001) and above (*ps* < .005) the mean on political orientation, participants perceived the professor in the professor in the inclusive flag versus no flag condition as more likable and reported more comfort and interest interacting; however, these effects were larger for liberal leaning participants (see Table S29).

At one standard deviation below the mean political orientation, participants reported more perceived similarity and willingness to select the professor in the inclusive flag versus no flag condition (*ps* $< .001$); however, these effects were not found for conservative leaning participants (*ps* $> .078$; see Table S29). At both one standard deviation below ($p < .001$) and above ($p = .001$) the mean on political orientation, participants perceived the professor in the no flag versus inclusive flag condition as more prejudiced; however, this effect was larger for liberal leaning participants (see Table S29). At one standard deviation below the mean on political orientation, participants perceived the professor in the no flag versus inclusive flag condition as more racist and sexist (*ps* $< .001$); however, these effects were not found for conservative leaning participants (*ps* $> .131$; see Table S29).

At one standard deviation below the mean on political orientation, participants perceived the professor in the no flag versus American flag condition as more likable, more similar, and reported more comfort and interest, and a higher likelihood of selecting the professor (*ps* $< .001$; see Table S29). At one standard deviation above the mean, the opposite pattern emerged, participants perceived the professor in the American flag versus no flag condition as more likable, more similar, and reported more comfort and interest, and a higher likelihood of selecting the professor (*ps* $< .037$; see Table S29). At one standard deviation above the mean on political orientation, the professor in the American flag versus no flag condition elicited more positive affect ($p < .001$). Although not significant, at one standard deviation below the mean, the opposite pattern emerged ($p = .095$), with the professor in the no flag versus American flag condition eliciting more positive affect. Lastly, at one standard deviation below the mean on political orientation, participants perceived the professor in the American flag versus no flag condition as higher in SDO ($p < .001$); however, at one standard deviation above the mean this effect was not significant ($p = .157$; see Table S29).

Table S29

Simple Effects of Flag Condition at 1 Standard Deviation Below and Above the Mean on the Political Orientation Scale from Supplemental Study 2.

Measures	No Flag (0) versus Inclusive Flag (1)						No Flag (0) versus American Flag (1)					
	Low Political Orientation			High Political Orientation			Low Political Orientation			High Political Orientation		
	b (SE)	*t* (536)	*p*	*b* (SE)	*t* (536)	*p*	*b* (SE)	*t* (536)	*p*	*b* (SE)	*t* (536)	*p*
Likability	3.39 (0.24)	13.85	< .001	0.86 (0.27)	3.20	.002	-1.01 (0.25)	-4.02	< .001	0.62 (0.26)	2.42	.016
Comfort and Interest	2.38 (0.21)	11.16	< .001	0.67 (0.23)	2.87	.004	-1.22 (0.22)	-5.60	< .001	0.47 (0.23)	2.10	.036
Perceived Similarity	1.93 (0.13)	14.44	< .001	0.24 (0.15)	1.65	.100	-0.90 (0.14)	-6.56	< .001	0.49 (0.14)	3.44	.001
Positive Affect	1.36 (0.15)	9.20	< .001	0.63 (0.16)	3.89	< .001	-0.25 (0.15)	-1.67	.095	0.60 (0.16)	3.82	< .001
Perceived Prejudice	-1.93 (0.14)	-13.80	< .001	-0.51 (0.15)	-3.32	.001	0.73 (0.14)	5.14	< .001	0.09 (0.15)	0.58	.561
Perceived Racism	-1.24 (0.13)	-9.91	< .001	-0.16 (0.14)	-1.16	.248	0.93 (0.13)	7.32	< .001	0.44 (0.13)	3.34	.001
Perceived Sexism	-1.17 (0.13)	-8.88	< .001	-0.22 (0.14)	-1.51	.132	0.69 (0.13)	5.18	< .001	0.40 (0.14)	2.86	.004
Professor SDO	-1.84 (0.17)	-10.73	< .001	-1.12 (0.19)	-5.92	< .001	1.40 (0.18)	7.96	< .001	0.26 (0.18)	1.42	.157
Professor Selection	2.45 (0.21)	11.44	< .001	0.41 (0.24)	1.76	.079	-1.35 (0.22)	-6.19	< .001	0.66 (0.23)	2.93	.004

Additional Measures, Analyses, and Results for Study 3

Method

Measures

The descriptives and correlations for all supplemental outcome variables in Study 3 are available in Table S30. Our Study 3 design was initially a 3 (timepoint) x 2 (participant race) x 2 (flag condition) between-subjects design. We examined whether the flag effects varied across three timepoints (July 4th, mid 2024 Summer Olympics, and post 2024 Summer Olympics) in order to explore whether the Olympics and July 4th would moderate our findings. Moreover, in addition to our measures in the main document,

participants also rated their agreement (1 – *Strongly disagree* to 7 – *Strongly agree*) to which they perceive the American flag as positive (*American flag perceptions*; 6 items; e.g., "The American flag is representative of me.") or threatening (*American flag threat perceptions*; 2 items; e.g., "When I see the American flag, I feel threatened.").

Table S30

Supplemental Measure information and statistics, reliabilities, and correlations for Study 3.

Measure	M (SD)	1	2	3	4	5
1. Positive Affect	2.17 (1.14)	(0.96)				
2. Negative Affect	1.48 (0.71)	$.13^{**}$	(0.93)			
3. Likability	5.08 (1.96)	$.52^{***}$	$-.30^{***}$	(NA)		
4. American Flag Perceptions	4.32 (1.69)	$.53^{***}$	$-.15^{***}$	$.50^{***}$	(0.94)	
5. American Flag Threat Perceptions	2.45 (1.63)	$-.23^{***}$	$.39^{***}$	$-.36^{***}$	$-.63^{***}$	(0.93)

Note. The reliability for each scale is shown on the diagonal. $^{*}p < .05$, $^{**}p < .01$, $^{***}p < .001$

Results

Across supplemental measures, we conducted separate 2 (participant race) × 2 (flag condition) × 3 (timepoint) between-subjects ANOVAs (see Tables S31 and S32 for means, standard deviations, contrasts between conditions, and full ANOVA results). Contrary to expectations, there was no main effect of timepoint (all *ps* > .062; see Table S32) and no significant interactions across outcomes (all *ps* > .218; see Table S32); however, we may have been underpowered to find the effect.

Table S31

Study 3 Supplemental Means and Standard Deviation across conditions.

Means and Standard Deviation Across Condition

Measures	July 4th								Mid-Olympics								Post-Olympics							
	No Flag				American Flag				No Flag				American Flag				No Flag				American Flag			
	White (n = 43)		Black (n = 43)		White (n = 49)		Black (n = 47)		White (n = 48)		Black (n = 45)		White (n = 47)		Black (n = 50)		White (n = 46)		Black (n = 44)		White (n = 44)		Black (n = 50)	
	M	SD	M	SD	M	SD	M	SD	M	SD	M	SD	M	SD	M	SD	M	SD	M	SD	M	SD	M	SD
Positive Affect	1.72	0.88	2.65	1.41	2.16	1.13	2.61	1.17	1.82	0.90	2.36	1.33	2.04	1.12	2.55	1.05	1.77	0.89	2.14	1.18	1.95	1.05	2.20	1.12
Negative Affect	1.34	0.65	1.44	0.76	1.50	0.69	1.54	0.77	1.43	0.61	1.46	0.72	1.58	0.73	1.80	0.82	1.35	0.74	1.44	0.62	1.44	0.63	1.45	0.67
Likability	5.21	1.36	5.63	2.27	4.80	2.35	5.38	1.96	4.54	1.81	5.53	1.74	5.13	2.39	4.86	2.01	4.96	1.35	5.14	2.04	4.91	1.95	5.02	1.86
Comfort and Interest	4.47	1.12	4.60	1.66	4.40	1.98	4.38	1.60	4.06	1.40	4.62	1.30	4.38	1.88	4.05	1.58	4.35	1.19	4.30	1.43	4.04	1.57	3.91	1.72
Perceived Racism	2.43	0.77	2.65	0.92	3.01	1.12	2.93	0.94	2.39	0.79	2.53	0.56	3.11	1.14	3.11	0.85	2.35	0.73	2.48	0.85	2.98	0.98	3.34	0.80
Perceived Sexism	2.48	0.69	2.59	0.95	2.84	1.20	2.79	0.90	2.60	0.87	2.47	0.64	3.01	1.12	2.93	0.93	2.38	0.81	2.53	0.93	2.86	0.89	2.96	0.87
Coworker SDO	3.58	0.92	3.51	1.38	4.22	1.52	3.70	1.44	3.59	1.02	3.39	0.89	4.43	1.61	4.12	1.26	3.46	0.83	3.26	1.15	4.19	1.22	4.59	1.31
American Flag Perceptions	4.10	1.52	4.59	1.85	4.07	1.65	4.58	1.46	4.34	1.70	4.36	1.66	4.53	1.76	4.24	1.58	4.35	1.73	4.21	1.67	4.46	1.81	4.07	1.88
American Flag Threat Perceptions	2.48	1.52	2.35	1.67	2.69	1.78	2.60	1.59	2.23	1.64	2.17	1.52	2.22	1.63	2.61	1.70	2.50	1.73	2.27	1.56	2.42	1.52	2.74	1.69

Measures	July 4th		Mid-Olympics		Post-Olympics		No Flag		American Flag		White		Black	
	M	SD	M	SD	M	SD	M	SD	M	SD	M	SD	M	SD
Positive Affect	2.29	1.21	2.19	1.14	2.02	1.07	2.07	1.16	2.26	1.12	1.92	1.01	2.42	1.22
Negative Affect	1.46	0.72	1.57	0.74	1.42	0.66	1.41	0.68	1.55	0.73	1.44	0.68	1.53	0.74
American Flag Perceptions	4.33	1.63	4.37	1.66	4.27	1.77	4.32	1.68	4.32	1.69	4.31	1.69	4.33	1.68
American Flag Threat Perceptions	2.54	1.64	2.31	1.63	2.49	1.63	2.33	1.60	2.55	1.65	2.42	1.64	2.47	1.62
Likability	5.24	2.04	5.01	2.02	5.01	1.80	5.16	1.81	5.01	2.09	4.92	1.92	5.25	1.98
Comfort and Interest	4.46	1.62	4.27	1.57	4.14	1.49	4.40	1.36	4.19	1.73	4.28	1.56	4.30	1.57
Perceived Racism	2.77	0.97	2.79	0.91	2.80	0.93	2.47	0.77	3.08	0.98	2.71	0.98	2.85	0.88
Perceived Sexism	2.68	0.96	2.76	0.93	2.69	0.90	2.51	0.82	2.90	0.99	2.70	0.97	2.72	0.89
Coworker SDO	3.77	1.36	3.89	1.29	3.89	1.26	3.47	1.04	4.21	1.41	3.92	1.27	3.79	1.33

Table S32

Study 3 Supplemental Between-Subjects ANOVA Results.

Supplemental Materials

Measures	ANOVA results												
	Timepoint condition			Flag Condition					Participant Race Condition				
	F (1,544)	*p*	η_p^2	*F* (1,544)	*p*	η_p^2	*MD* (*SE*)	*d*	*F* (1,544)	*p*	η_p^2	*MD* (*SE*)	*d*
Positive Affect	2.78	.063	.010	3.51	.062	.006	0.18 (0.09)	-0.16	28.84	<.001	.050	0.51 (0.09)	-0.45
Negative Affect	2.19	.113	.008	5.62	.018	.010	0.14 (0.06)	-0.20	1.81	.179	.003	0.08 (0.06)	-0.12
Likability	0.95	.388	.003	0.83	.362	.002	0.15 (0.17)	0.08	4.11	.043	.008	0.34 (0.17)	-0.17
Comfort and Interest	1.84	.160	.007	2.48	.116	.005	0.21 (0.13)	0.13	0.07	.798	<.001	0.03 (0.13)	-0.01
Perceived Racism	0.08	.925	.000	64.99	<.001	.107	0.61 (0.08)	-0.69	2.83	.093	.005	0.13 (0.08)	-0.15
Perceived Sexism	0.40	.668	.001	25.09	<.001	.044	0.39 (0.08)	-0.43	0.06	.813	<.001	0.02 (0.08)	-0.03
Coworker SDO	0.64	.531	.002	49.43	<.001	.083	0.74 (0.11)	-0.60	1.99	.159	.004	0.15 (0.11)	0.10
American Flag Perceptions	0.15	.861	.001	0.00	.995	<.001	0.00 (0.14)	0.00	0.05	.822	<.001	0.03 (0.14)	-0.02
American Flag Threat Perceptions	0.96	.385	.004	2.39	.122	.004	0.22 (0.14)	-0.14	0.05	.819	<.001	0.03 (0.82)	-0.03

Measures	Differences between Timepoint Conditions								
	July 4th vs. Mid-Olympics			July 4th vs. Post-Olympics			Mid-Olympics vs. Post-Olympics		
	MD (*SE*)	*p*	*d*	*MD* (*SE*)	*p*	*d*	*MD* (*SE*)	*p*	*d*
Positive Affect	0.10 (0.12)	1.00	0.08	0.27 (0.12)	.062	0.24	0.18 (0.12)	.388	0.16
Negative Affect	0.11 (0.07)	.436	-0.15	0.04 (0.07)	1.00	0.06	0.15 (0.07)	.131	-0.15
Likability	0.24 (0.20)	.722	0.11	0.25 (0.21)	.675	0.12	0.10 (0.20)	1.00	0.00
Comfort and Interest	0.18 (0.16)	.811	0.11	0.31 (0.16)	.169	0.20	0.13 (1.6)	1.00	0.09
Perceived Racism	0.03 (0.09)	1.00	-0.02	0.03 (0.09)	1.00	-0.04	0.01 (0.09)	1.00	-0.01
Perceived Sexism	0.08 (0.10)	1.00	-0.08	0.01 (0.10)	1.00	-0.01	0.07 (0.10)	1.00	0.08
Coworker SDO	0.13 (0.13)	.943	-0.09	0.12 (0.13)	1.00	-0.10	0.01 (0.13)	1.00	-0.00
American Flag Perceptions	0.04 (0.18)	1.00	-0.02	0.06 (0.18)	1.00	0.04	0.10 (0.18)	1.00	-0.02
American Flag Threat Perceptions	0.22 (0.17)	.578	0.14	0.05 (0.17)	1.00	0.03	0.18 (0.17)	.897	0.14

Measures	Participant Race × Flag			Participant Race × Timepoint			Timepoint × Flag			Participant Race × Flag × Timepoint		
	F (1,544)	*p*	η_p^2	*F* (1,544)	*p*	η_p^2	*F* (1,544)	*p*	η_p^2	*F* (1,544)	*p*	η_p^2
Positive Affect	1.16	.282	.002	1.34	.262	.005	0.09	.919	<.001	0.53	.590	.002
Negative Affect	0.03	.869	<.001	0.14	.144	.866	0.86	.862	.003	0.49	.616	.002
Likability	1.36	.245	.002	0.39	.679	.001	0.29	.749	.001	1.79	.168	.007
Comfort and Interest	2.08	.150	.004	0.24	.785	.001	0.27	.767	.001	1.07	.345	.004
Perceived Racism	0.23	.632	<.001	0.55	.577	.002	1.57	.210	.006	1.08	.341	.004
Perceived Sexism	0.16	.688	<.001	0.76	.467	.003	0.50	.610	.002	0.14	.868	.001

Coworker SDO	0.01	.931	<.001	1.41	.245	.005	2.85	.059	.010	2.11	.122	.008
American Flag Perceptions	0.40	.530	.001	2.69	.069	.010	0.01	.988	<.001	0.13	.878	<.001
American Flag Threat Perceptions	1.52	.219	.003	0.33	.719	.001	0.01	.994	<.001	0.32	.725	.001

Note. MD = Mean Difference. SE = Standard Error.

Moderation by Participant Social Dominance Orientation (SDO)

To examine whether participant SDO moderated timepoint and flag condition, we ran a regression model with timepoint dummy coded and the Post-Olympics timepoint as the reference group, flag condition (0 = no flag and 1 = American flag), participant SDO, and all interactions. We found no significant interactions with participant SDO, timepoint, and flag condition ($p > .085$; see Table S33). We then examined whether participant SDO moderated participant race (0 = White participants and 1 = Black participants) and flag condition and found no significant three-way interactions (all $ps > .091$; see Table S33). However, we did find significant Flag Condition x Participant SDO interactions on all outcome variables (all $ps < .014$; see Table S33), except for negative affect ($p =$ .279) and American flag threat perceptions ($p = .101$; see Table S33), so we probed the significant interactions. At one standard deviation below the mean on SDO, participants in the American flag condition viewed the coworker as less likable and indicated less comfort and interest in interacting with the coworker, compared to those in the no flag condition ($ps < .004$; see Table S33). In contrast, at one standard deviation above the mean on participant SDO, the opposite effects emerged. Participants perceived the coworker in the American flag condition as more likable and reported more comfort and interest interacting with the coworker relative to those in the no flag condition ($ps < .046$; see Table 33).

Additionally, participants one standard deviation below the mean on participant SDO perceived the coworker in the American versus no flag condition as more racist (*p* < .001), as more sexist (*p* < .001), and as high in SDO (*p* < .001; see Table S33). Participants one standard deviation above the mean on participant SDO also viewed the coworker as higher in SDO in the American flag versus no flag condition (*p* = .030; see Table 33), although this effect was weaker compared to participants lower in participant SDO. Moreover, participants with high levels of participant SDO (one standard deviation above the mean) did not differ between the American and no flag condition on perceived coworker racism (*p* = .082) and sexism (*p* = .937; see Table 33). Finally, participants at one standard deviation above the mean on participant SDO, reported more positive affect and more positive American flag perceptions (*p* < .029); however, these effects were not significant for participants at one standard deviation below the mean (see Table S33).

Table S33

Study 3 Supplemental Interactions with Participant SDO, Timepoint, Participant Race, and Flag Condition.

Testing for 3-way Interactions with Flag Condition, Timepoint, and Participant SDO						
Measures	Post-Olympics vs. July 4th × Flag Condition × Participant SDO			Post-Olympics vs. Mid-Olympics × Flag Condition × Participant SDO		
	b (SE)	*t* (544)	*p*	*b* (SE)	*t* (544)	*p*
Positive Affect	-0.15 (0.20)	-0.75	.453	0.10	0.20	.517
Negative Affect	-0.07 (0.13)	-0.55	.580	-0.07 (0.13)	-0.57	.572
Likability	-0.44 (0.34)	-1.30	.196	-0.24 (0.34)	-0.70	.484
Comfort and Interest	-0.09 (0.28)	-0.32	.745	-0.00 (0.28)	-0.02	.987
Perceived Racism	-0.02 (0.16)	-0.15	.884	-0.07 (0.16)	-0.46	.643
Perceived Sexism	-0.02 (0.16)	-0.10	.918	0.02 (0.16)	0.10	.917
Coworker SDO	-0.13 (0.23)	-0.59	.556	-0.07 (0.23)	-0.29	.770
American Flag Perceptions	-0.46 (0.29)	-1.57	.118	-0.50 (0.29)	-1.72	.086
American Flag Threat Perceptions	0.47 (0.29)	1.60	.109	0.19 (0.29)	0.67	.505
Testing for 2-way interactions between Timepoint and Participant SDO						
Measures	Post-Olympics vs. July 4th × Participant SDO			Post-Olympics vs. Mid-Olympics × Participant SDO		
	b (SE)	*t* (544)	*p*	*b* (SE)	*t* (544)	*p*
Positive Affect	-0.12 (0.10)	-1.18	.240	-0.11 (0.10)	-1.12	.263
Negative Affect	0.01 (0.06)	0.10	.923	-0.07 (0.06)	-1.15	.249

Likability	-0.03 (0.17)	-0.19	.847	-0.18 (0.17)	-1.006	.291
Comfort and Interest	-0.05 (0.14)	-0.38	.708	-0.00 (0.14)	-0.02	.984
Perceived Racism	0.03 (0.08)	0.37	.715	0.10 (0.08)	1.26	.208
Perceived Sexism	-0.00 (0.08)	-0.01	.991	-0.03 (0.08)	-0.36	.722
Coworker SDO	0.04 (0.11)	0.34	.733	-0.01 (0.11)	-0.08	.934
American Flag Perceptions	-0.20 (0.15)	-1.34	.182	-0.06 (0.15)	-0.39	.696
American Flag Threat Perceptions	-0.13 (0.15)	-0.88	.381	-0.13 (0.15)	-0.89	.373

Testing for 2-way Interactions and 3-way Interactions with Flag Condition, Participant Race, and Participant SDO

Measures	Flag × SDO			Flag × Participant Race × SDO		
	b (SE)	*t* (548)	*p*	*b* (SE)	*t* (548)	*p*
Positive Affect	0.25 (0.08)	3.11	.002	0.09 (0.16)	0.60	.551
Negative Affect	-0.06 (0.05)	-1.08	.279	0.17 (0.10)	1.68	.094
Likability	0.57 (0.14)	4.17	<.001	-0.33 (0.27)	-1.19	.234
Comfort and Interest	0.45 (0.11)	4.06	<.001	-0.26 (0.22)	-1.18	.238
Perceived Racism	-0.36 (0.06)	-5.72	<.001	0.21 (0.13)	1.69	.092
Perceived Sexism	-0.33 (0.07)	-5.06	<.001	0.14 (0.13)	1.07	.286
Coworker SDO	-0.35 (0.09)	-3.87	<.001	-0.14 (0.18)	-0.75	.457
American Flag Perceptions	0.29 (0.12)	2.49	.013	-0.07 (0.24)	-0.29	.769
American Flag Threat Perceptions	-0.19 (0.12)	-1.64	.101	0.13 (0.23)	0.58	.565

Simple of Effects of Flag Condition at 1 Standard Deviation Above and Below the Mean on the Participant SDO Scale

Measures	1 Standard Deviation Below			1 Standard Deviation Above		
	b (SE)	*t* (552)	*p*	*b* (SE)	*t* (552)	*p*
Positive Affect	-0.03 (0.13)	-0.26	.795	0.54 (0.13)	4.08	< .001
Likability	-0.65 (0.22)	-3.01	.003	0.62 (0.23)	2.75	.006
Comfort and Interest	-0.62 (0.18)	-3.51	.001	0.37 (0.18)	2.01	.045
Perceived Racism	0.97 (0.10)	9.72	<.001	0.18 (0.10)	1.74	.082
Perceived Sexism	0.72 (0.10)	6.98	<.001	-0.01 (0.11)	-0.08	.937
Coworker SDO	1.13 (0.14)	7.84	<.001	0.33 (0.15)	2.18	.030
American Flag Perceptions	-0.21 (0.19)	-1.12	.264	0.43 (0.19)	2.20	.028

Additional Measures, Analyses, and Results for Study 3: Part 2

Moderation by Participant Political Orientation

We next examined whether political orientation (1 = *Extremely Liberal* to 7 = *Extremely Conservative*) moderated timepoint and flag condition, we ran a regression model with timepoint dummy coded and the Post-Olympics timepoint as the reference group, flag condition (0 = no flag and 1 = American flag), political orientation, and all interactions. We did not find any significant three-way

interactions between the Post-Olympics versus July 4th contrast (*ps* > .049), race, and political orientation predicting any of the outcome variables (see Table S34). We also did not find any significant three-way interactions between the Post-Olympics versus Mid-Olympics contrast (*ps* > .101), race, and political orientation predicting any of the outcome variables (see Table S34). However, for all outcome measures, we examined two-way interactions between condition dummy coded (with Post-Olympics timepoint as the reference group), and political orientation (all two-way interactions are reported in Table S34). We only found a significant Post-Olympics vs. July 4th x Political Orientation interaction on likability ($p = .026$; see Table S34). When we examined the effect of timepoint at one standard deviation below and above the mean on political orientation (see Table S20), we found that although not significant at one standard deviation below ($p = .759$) or above ($p = .060$), both liberal and conservative leaning participants perceived the coworker as more likable during the July 4th versus post-Olympics timepoint; however, the effect was larger for conservative leaning participants (see Table S34). We also found a Post-Olympics vs. Mid-Olympics x Political Orientation interaction on likability ($p = .032$; see Table S34). When we examined the effect of timepoint at one standard deviation below and above the mean on political orientation, we found that although not significant at one standard deviation below ($p = .563$) or above ($p = .494$), liberal leaning participants perceived the coworker as more likable during the post-Olympics versus mid-Olympics timepoint. We found the opposite effect for conservative leaning participants who perceived the coworker as more likable during the mid-Olympics versus post-Olympic timepoint (see Table S34).

We then examined whether political orientation moderated participant race (0 = White participants and 1 = Black participants) and flag condition and found significant Flag Condition x Political Orientation interactions on likability, comfort and interest,

perceived racism, perceived sexism, and coworker SDO (*ps* < .044; see Table S34), so we probed those interactions. At one standard deviation below the mean on political orientation, participants perceived the coworker in the no flag versus American flag condition as more likable (*p* = .001). At one standard deviation above the mean, the opposite pattern emerged, participants perceived the coworker in the American flag versus no flag condition as more likable (*p* = .011; see Table S34). At one standard deviation below the mean on political orientation, participants reported more comfort and interest interacting with the coworker in the no flag versus American flag condition (*p* = .016). At one standard deviation above the mean, although not significant (*p* = .729), conservative leaning participants had more comfort and interest interacting with the coworker in the American flag versus no flag condition (see Table S34). At both one standard deviation below (*ps* < .001) and above (*ps* = .001) the mean on political orientation, participants perceived the coworker in the American flag versus no flag condition as more racist and higher in SDO; however, this effect was larger for liberal leaning participants (see Table S34). At one standard deviation below the mean on political orientation, participants perceived the coworker in the American flag versus no flag condition as more sexist (*p* < .001); however, this effect was not significant for conservative participants (*p* = .071; see Table S34).

Table S34

Study 3 Supplemental Interactions with Political Orientation, Timepoint, Participant Race, and Flag Condition.

Testing for 3-way Interactions with Flag Condition, Timepoint, and Political Orientation						
Measures	Post-Olympics vs. July 4th × Flag Condition × Political Orientation			Post-Olympics vs. Mid-Olympics × Flag Condition × Political Orientation		
	b (SE)	*t* (544)	*p*	*b* (SE)	*t* (544)	*p*
Likability	0.15 (0.23)	0.66	.507	0.19 (0.23)	0.87	.387
Comfort and Interest	0.22 (0.19)	1.20	.231	0.03 (0.19)	0.15	.879
Positive Affect	0.11 (0.14)	0.81	.417	0.16 (0.14)	1.20	.229
Perceived Racism	-0.21 (0.11)	-1.96	.050	-0.18 (0.11)	-1.64	.102
Perceived Sexism	-0.11 (0.11)	-0.99	.321	-0.07 (0.11)	-0.66	.508

Measures	b (SE)	t	p	b (SE)	t	p
Coworker SDO	-0.27 (0.15)	-1.73	.084	-0.21 (0.15)	-1.40	.162
Negative Affect	-0.10 (0.09)	-1.15	.251	-0.02 (0.09)	-0.23	.818
American Flag Perceptions	0.01 (0.19)	0.03	.977	-0.04 (0.19)	-0.23	.818
American Flag Threat Perceptions	-0.06 (0.19)	-0.30	.761	-0.11 (0.19)	-0.59	.552
Testing for 2-way interactions between Timepoint and Political Orientation						
Measures	Post-Olympics vs. July 4th × Political Orientation			Post-Olympics vs. Mid-Olympics × Political Orientation		
	b (SE)	*t* (544)	*p*	*b* (SE)	*t* (544)	*p*
Likability	0.25 (0.11)	2.24	.026	0.24 (0.11)	2.15	.032
Comfort and Interest	0.17 (0.09)	1.84	.067	0.13 (0.09)	1.44	.150
Positive Affect	0.08 (0.07)	1.10	.274	-0.05 (0.07)	-0.70	.484
Perceived Racism	-0.08 (0.05)	-1.50	.135	-0.02 (0.05)	-0.33	.738
Perceived Sexism	-0.02 (0.06)	-0.41	.679	-0.01 (0.06)	-0.12	.904
Coworker SDO	-0.01 (0.08)	-0.09	.929	-0.01 (0.08)	-0.17	.865
Negative Affect	0.02 (0.04)	0.36	.718	-0.02 (0.04)	-0.52	.601
American Flag Perceptions	-0.00 (0.09)	-0.04	.971	-0.01 (0.09)	-0.08	.936
American Flag Threat Perceptions	-0.10 (0.10)	-1.03	.305	-0.09 (0.10)	-0.96	.339
Testing for 2-way Interactions and 3-way Interactions with Flag Condition, Participant Race, and Political Orientation						
Measures	Flag × Political Orientation			Flag × Participant Race × Political Orientation		
	b (SE)	*t* (548)	*p*	*b* (SE)	*t* (548)	*p*
Likability	0.41 (0.09)	4.59	<.001	-0.47 (0.18)	-2.64	.009
Comfort and Interest	0.15 (0.07)	2.03	.043	-0.42 (0.15)	-2.82	.005
Positive Affect	0.06 (0.05)	1.07	.287	-0.20 (0.11)	-1.83	.068
Perceived Racism	-0.14 (0.04)	-3.19	.002	0.18 (0.09)	2.10	.036
Perceived Sexism	-0.12 (0.04)	-2.64	.008	0.14 (0.09)	1.63	.103
Coworker SDO	-0.18 (0.06)	-2.87	.004	0.07 (0.12)	0.54	.587
Negative Affect	-0.07 (0.04)	-1.91	.057	0.04 (0.07)	0.51	.613
American Flag Perceptions	0.02 (0.07)	0.27	.785	0.02 (0.15)	0.13	.900
American Flag Threat Perceptions	-0.07 (0.07)	-0.96	.335	-0.20 (0.15)	-1.36	.174
Simple of Effects of Flag Condition (Post-Olympics vs. July 4th) at 1 Standard Deviation Above and Below the Mean on the Political Orientation Scale						
Measures	1 Standard Deviation Below			1 Standard Deviation Above		
	b (SE)	*t* (552)	*p*	*b* (SE)	*t* (552)	*p*
Likability	0.11 (0.36)	0.31	.759	0.74 (0.39)	1.88	.060
Simple of Effects of Flag Condition (Post-Olympics vs. Mid-Olympics) at 1 Standard Deviation Above and Below the Mean on the Political Orientation Scale						
Measures	1 Standard Deviation Below			1 Standard Deviation Above		
	b (SE)	*t* (552)	*p*	*b* (SE)	*t* (552)	*p*
Likability	-0.21 (0.35)	-0.58	.563	0.24 (0.35)	0.68	.494
Simple of Effects of Flag Condition at 1 Standard Deviation Above and Below the Mean on the Political Orientation Scale						
Measures	1 Standard Deviation Below			1 Standard Deviation Above		
	b (SE)	*t* (552)	*p*	*b* (SE)	*t* (552)	*p*
Likability	-0.68 (0.20)	-3.41	.001	0.53 (0.21)	2.55	.011
Comfort and Interest	-0.40 (0.16)	-2.42	.016	0.06 (0.17)	0.35	.729
Perceived Racism	0.81 (0.09)	8.54	<.001	0.37 (0.10)	3.70	.001
Perceived Sexism	0.55 (0.10)	5.69	<.001	0.19 (0.10)	1.81	.071

Coworker SDO	0.97 (0.13)	7.27	<.001	0.46 (0.14)	3.21	.001

Additional Measures, Analyses, and Results for Study 3: Part 3

Moderation by Participant Gender

We found a significant main effect of participant gender for all outcome variables (*ps* < .017; see Table S36). Women participants perceived the coworker as more sexist, more racist, and higher in SDO than men participants. In contrast, men participants reported more comfort and interest interacting with the coworker than women participants. We also found a significant main effect of flag condition across all outcome variables (all *ps* < .001; see Table S36), except for comfort and interest (*p* = .091). Participants perceived the professor in the American flag versus no flag condition as more racist, more sexist, and higher in SDO. We did not find any significant interactions (*ps* > .437; see Table S36).

Table S35

Study 3 Means and Standard Deviation across conditions.

Means and Standard Deviation Across Condition								
Measures	Men				Women			
	No Flag (*n* = 130)		American Flag (*n* = 151)		No Flag (*n* = 139)		American Flag (*n* = 136)	
	M	*SD*	*M*	*SD*	*M*	*SD*	*M*	*SD*
Comfort and Interest	4.55	1.23	4.43	1.63	4.25	1.47	3.93	1.80
Perceived Racism	2.35	0.77	2.95	0.96	2.58	0.76	3.23	0.97
Perceived Sexism	2.40	0.77	2.82	0.99	2.61	0.85	2.98	0.98
Coworker SDO	3.36	1.00	4.06	1.36	3.56	1.07	4.37	1.47
Measures	No Flag		American Flag		Men		Women	

	M	*SD*	*M*	*SD*	*M*	*SD*	*M*	*SD*
Comfort and Interest	4.40	1.36	4.19	1.73	4.49	1.45	4.09	1.65
Perceived Racism	2.47	0.77	3.08	0.98	2.67	0.93	2.90	0.93
Perceived Sexism	2.51	0.82	2.90	0.99	2.63	0.92	2.80	0.93
Coworker SDO	3.47	1.04	4.21	1.42	3.74	1.25	3.96	1.34

Table S36

Study 3 Between-Subjects ANOVA Results.

	**ANOVA results**												
Measures	Participant Gender Condition					Flag Condition					Participant Gender x Flag Condition		
	F (1,552)	*p*	η_p^2	*MD* (*SE*)	*d*	*F* (1,552)	*p*	η_p^2	*MD* (*SE*)	*d*	*F* (1,552)	*p*	η_p^2
Comfort and Interest	9.30	.002	.017	0.40 (0.13)	0.26	2.87	.091	.005	0.22 (0.13)	0.13	0.60	.438	.001
Perceived Racism	11.72	<.001	.021	0.26 (0.07)	0.25	69.97	<.001	.112	0.62 (0.07)	0.69	0.16	.692	.000
Perceived Sexism	5.97	.015	.011	0.19 (0.08)	0.18	26.69	<.001	.046	0.40 (0.08)	0.43	0.14	.705	.000
Coworker SDO	5.83	.016	.010	0.26 (0.11)	0.17	51.07	<.001	.085	0.76 (0.11)	0.60	0.25	.618	.000

Perceptions of Coworker Race

We found a significant main effect of coworker race among all outcome variables (all *ps* < .013; see Table S38). Participants who perceived the coworker as White (vs. other race) reported more perceptions of racism, sexism, SDO, and less comfort and interest interacting with the coworker. We also found a significant main effect of flag condition on all outcome variables (all *ps* < .024; see Table S38), except for comfort and interest (*p* = .751) and perceived sexism (*p* = .245). Participants in the American flag versus no

flag condition perceived the coworker as more racist and higher in SDO. Lastly, we found significant coworker race by flag condition interactions on perceived sexism (p = .005) and coworker SDO (p = .002). For perceived sexism, in the American flag condition, participants who perceived the coworker as White (vs. other race) reported more perceptions of sexism, *mean difference* = 0.66, *standard error* = 0.17, p < .001, d = 0.71. This effect was not significant in the no flag condition (p = .946). For coworker SDO, in the American flag condition, participants who perceived the coworker as White (vs. other race) reported more perceptions of SDO, *mean difference* = 1.08, *standard error* = 0.24, p < .001, d = 0.85. This effect was not significant in the no flag condition (p = .564).

Table S37

Study 3 Means and Standard Deviation across conditions.

Means and Standard Deviation Across Condition								
Measures	Other Race				White			
	No Flag (n = 44)		American Flag (n = 30)		No Flag (n = 225)		American Flag (n = 257)	
	M	*SD*	*M*	*SD*	*M*	*SD*	*M*	*SD*
Comfort and Interest	5.00	1.12	5.34	1.33	4.28	1.38	4.06	1.72
Perceived Racism	2.41	.678	2.63	0.91	2.48	0.79	3.13	0.97
Perceived Sexism	2.50	0.69	2.31	0.90	2.51	0.84	2.97	0.98
Coworker SDO	3.37	1.04	3.24	1.11	3.49	1.04	4.32	1.411
Measures	No Flag		American Flag		Other Race		White	
	M	*SD*	*M*	*SD*	*M*	*SD*	*M*	*SD*
Comfort and Interest	4.4	1.36	4.19	1.73	5.14	1.21	4.16	1.57
Perceived Racism	2.47	0.77	3.08	0.98	2.50	0.78	2.83	0.95
Perceived Sexism	2.51	0.82	2.90	0.99	2.42	0.78	2.75	0.94
Coworker SDO	3.47	1.04	4.21	1.42	3.32	1.07	3.93	1.32

Table S38

Study 3 Between-Subjects ANOVA Results.

Measures	ANOVA results												
	Coworker Race Condition					Flag Condition					Coworker Race x Flag Condition		
	F (1,552)	*p*	η_p^2	*MD* (*SE*)	*d*	*F* (1,552)	*p*	η_p^2	*MD* (*SE*)	*d*	*F* (1,552)	*p*	η_p^2
Comfort and Interest	26.85	<.001	.046	1.00 (0.19)	0.70	0.10	.751	.000	0.06 (0.19)	0.13	2.13	.145	.004
Perceived Racism	63.7	.012	.011	0.28 (0.11)	0.38	15.28	<.001	.027	0.44 (0.11)	0.69	3.80	.052	.007
Perceived Sexism	8.59	.004	.015	0.33 (0.11)	0.38	1.35	.245	.002	0.13 (0.11)	0.43	8.08	.005	.014
Coworker SDO	14.82	<.001	.026	0.60 (0.16)	0.51	5.20	.023	.009	0.36 (0.16)	0.60	9.61	.002	.017

Perceptions of Coworker Gender

We found a significant main effect of coworker gender among all outcome variables (all *ps* < .035), except for perceived racism (*p* = .211) and coworker SDO (*p* = .252; see Table S40). Participants who perceived the coworker as a man (versus a woman) reported more perceptions of sexism, and less comfort and interest interacting with the coworker. We also found a significant main effect of flag condition on coworker SDO (*p* = .028), but not on any of the other outcome variables (all *ps* > .066; see Table S40). Participants perceived the coworker in the American flag versus no flag condition as higher in SDO. Lastly, we found a significant coworker gender by flag condition interaction on comfort and interest (*p* = .029; see Table S40). Participants who perceived the coworker as a man (vs. a woman) reported less comfort and interest interacting with the coworker in the American flag condition, *mean difference* = 1.76, *standard error* = 0.59, *p* = .003, *d* = 1.26. This effect was not significant in the no flag condition (*p* = .968).

Table S39

Study 3 Means and Standard Deviation across conditions.

Measures	Man				Woman			
	No Flag (n = 261)		American Flag (n = 280)		No Flag (n = 8)		American Flag (n = 7)	
	M	*SD*	*M*	*SD*	*M*	*SD*	*M*	*SD*
Comfort and Interest	4.40	1.38	4.15	1.72	4.38	0.60	5.90	0.94
Perceived Racism	2.47	0.78	3..09	0.98	2.38	0.63	2.61	0.98
Perceived Sexism	2.51	0.83	2.92	0.98	2.38	0.57	1.93	1.03
Coworker SDO	3.48	1.04	4.22	1.43	3.13	1.13	3.82	0.81
**Measures**	**No Flag**		**American Flag**		**Man**		**Woman**	
	M	*SD*	*M*	*SD*	*M*	*SD*	*M*	*SD*
Comfort and Interest	4.40	1.36	4.19	1.73	4.27	1.57	5.09	1.09
Perceived Racism	2.47	0.77	3.08	0.98	2.79	0.94	2.48	0.79
Perceived Sexism	2.51	0.82	2.90	0.99	2.72	0.93	2.17	0.82
Coworker SDO	3.47	1.04	4.21	1.42	3.86	1.31	3.45	1.02

Table S40

Study 3 Between-Subjects ANOVA Results.

Measures	Coworker Gender Condition					Flag Condition					Coworker Gender x Flag Condition		
	F (1,552)	*p*	η_p^2	*MD* (*SE*)	*d*	*F* (1,552)	*p*	η_p^2	*MD* (*SE*)	*d*	*F* (1,552)	*p*	η_p^2
Comfort and Interest	4.53	.034	.008	0.87 (0.41)	0.61	2.47	.116	.004	0.64 (0.41)	0.13	4.76	.029	.009
Perceived Racism	1.57	.211	.003	0.29 (0.23)	0.36	3.38	.067	.006	0.43 (0.23)	0.69	0.70	.403	.001

Perceived Sexism	5.69	.017	.010	0.57 (0.24)	0.63	0.01	.939	.000	0.02 (0.24)	0.43	3.26	.072	.006
Coworker SDO	1.31	.252	.002	0.38 (0.33)	0.40	4.82	.028	.009	0.72 (0.33)	0.60	0.01	.944	.000

Initial Supplemental Study

We ran an initial study before Study 1 to test our design. This non-preregistered initial study had a between-subject experimental design (Denver Broncos flag vs. American flag); and thus, no true control condition. Participants were recruited from a western university in the U.S. and the procedures and measures were the same as Study 1. Materials, data, and syntax are available on OSF page: https://osf.io/n2a85/overview?view_only=7cf6ae79ec31441bbe14d39b56ef6fa7. Reliabilities and correlations for all the outcomes used in the initial study are presented in Table S41.

Table S41

Measure information and statistics, reliabilities, and correlations for the Initial Supplemental Study.

Measure	M (SD)	1	2	3	4	5	6	7	8	9	10	11	12	13
1. Likability	5.37 (1.80)	(NA)												
2. Comfort and Interest	4.40 (1.54)	.73***	(0.93)											
3. Perceived Similarity	2.66 (0.91)	.68***	.67***	(0.92)										
4. Positive Affect	2.15 (0.93)	.51***	.52***	.51***	(0.94)									
5. Negative Affect	1.51 (0.68)	-.36***	-.40***	-.32***	.17**	(0.93)								
6. Perceived Prejudice	3.55 (0.89)	-.72***	-.71***	-.58***	-.56***	.42***	(0.92)							
7. Perceived Racism	2.43 (0.89)	-.63***	-.66***	-.58***	-.49***	.39***	.72***	(0.80)						

8. Perceived Sexism	2.48 (0.95)	-.54***	-.61***	-.47***	-.28***	.45***	.62***	.67***	(0.87)					
9. Race-based Classroom Mistreatment	2.01 (1.38)	-.35***	-.44***	-.38***	-.20**	.37***	.44***	.55***	.51***	(0.97)				
10. Gender-based Classroom Mistreatment	2.32 (1.56)	-.44***	-.55***	-.48***	-.25***	.48***	.55***	.66***	.68***	.75***	(0.97)			
11. Professor SDO	3.65 (1.11)	-.49***	-.50***	-.51***	-.25***	.45***	.61***	.62***	.63***	.43***	.55***	(0.86)		
12. Professor Selection	4.34 (1.56)	.81***	.69***	.63***	.51***	-.29***	-.67***	-.59***	-.57***	-.44***	-.46***	-.49***	(NA)	
13. Participant SDO	1.82 (0.94)	.24***	.16*	.18**	.23***	.04	-.14*	-.18**	-.14*	-.08	-.16*	.03	.25***	(0.84)

Note. The reliability for each scale is shown on the diagonal. $^{*}p < .05$, $^{**}p < .01$, $^{***}p < .001$

Results

To examine the influence of the flags on perceptions of the professor, we conducted separate independent samples T-tests (Denver Broncos flag vs. American flag) for each dependent variable. Means, standard deviations, and full T-test results are reported in Table S42.

Table S42

Initial Supplemental Study Means, Standard Deviation, T-test results across conditions.

Means, Standard Deviation, and T-test Results Across Condition									
Measures	Denver Broncos Flag		American Flag		*t*	*df*	*p*	*MD (SE)*	*d*
	M	*SD*	*M*	*SD*					
Likability	5.98	1.44	4.73	1.91	5.58	208.16	<.001	1.25 (0.22)	0.74
Comfort and Interest	4.83	1.38	3.98	1.58	4.38	227.81	<.001	0.85 (0.19)	0.57
Perceived Similarity	2.95	0.81	2.39	0.93	4.87	223.50	<.001	0.56 (0.12)	0.64
Positive Affect	2.28	0.97	2.02	0.88	2.16	229.07	.032	0.26 (0.12)	0.28
Negative Affect	1.37	0.60	1.65	0.73	-3.33	229.33	.001	-0.29 (0.09)	-0.43
Perceived Prejudice	3.26	0.80	3.84	0.88	-5.30	233.93	<.001	-0.58 (0.11)	-0.69
Perceived Racism	2.06	0.72	2.79	0.90	-6.78	223.33	<.001	-0.72 (0.11)	-0.89
Perceived Sexism	2.20	0.86	2.77	0.95	-4.78	222.62	<.001	-0.57 (0.12)	-0.63
Race-based Classroom Mistreatment	1.76	1.18	2.28	1.53	-2.82	199.58	.005	-0.52 (0.18)	-0.38
Gender-based Classroom Mistreatment	2.05	1.38	2.61	1.69	-2.69	204.83	.008	-0.56 (0.21)	-0.36
Professor SDO	3.32	0.93	4.01	1.18	-4.86	205.04	< .001	-0.68 (0.14)	-0.64
Professor Selection	4.83	1.29	3.84	1.65	5.05	211.69	< .001	0.99 (0.20)	0.67

We found significant effects for all outcome variables (all *ps* $< .033$; see Table S42). Participants perceived the professor in the Denver Broncos flag versus American flag condition as more likable ($p < .001$), similar ($p < .001$), reported more comfort and interest ($p < .001$), positive affect ($p = .032$), and were more likely to select the professor ($p < .001$; see Table S42). In contrast, participants in the American flag versus Denver Broncos flag condition reported more prejudice ($p < .001$), racism ($p < .001$), sexism ($p < .001$), SDO ($p < .001$), negative affect ($p = .001$), and anticipated more race-based classroom mistreatment ($p = .005$), and gender-based classroom mistreatment ($p = .008$; see Table S42).

Zoom Background Pictures Used Across Studies

Studies 1-3: No Flag Zoom Background

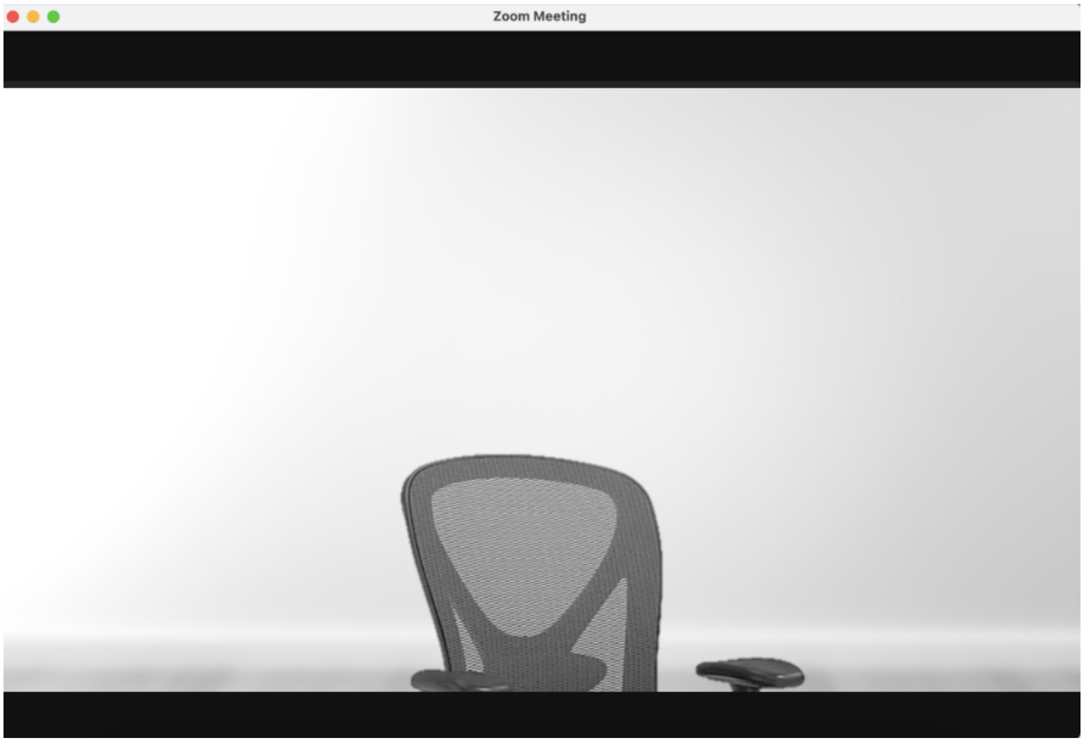

Study 1: Denver Broncos Flag Zoom Background

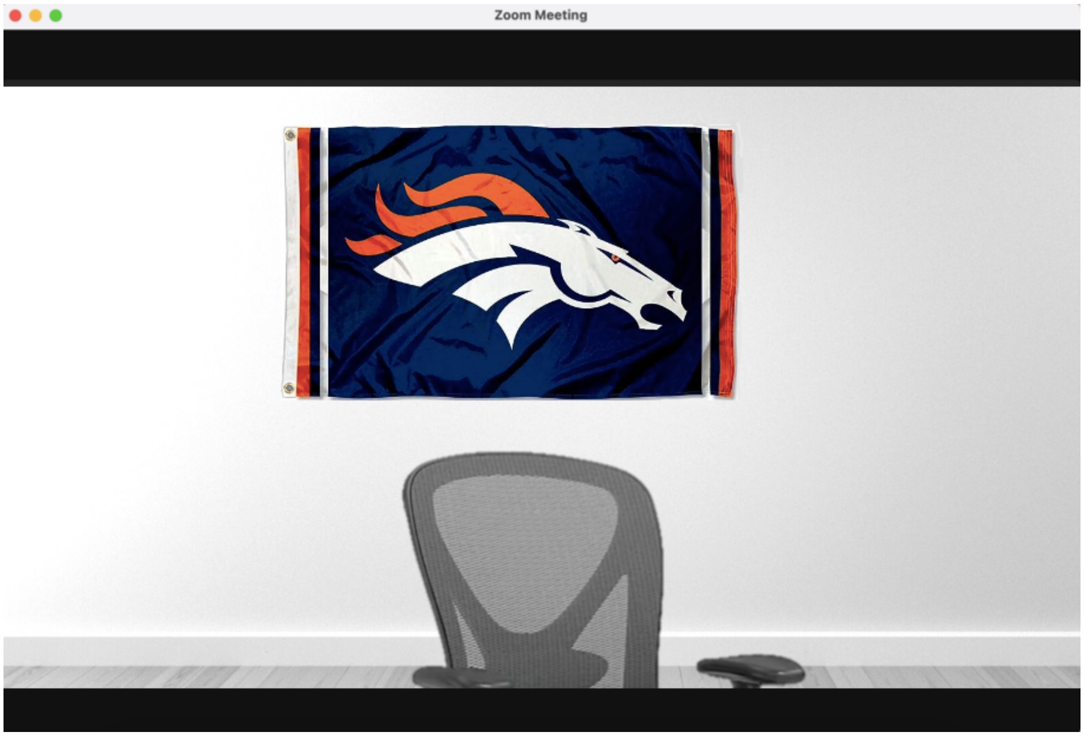

Supplemental Materials

Study 2: Inclusive Flag Zoom Background

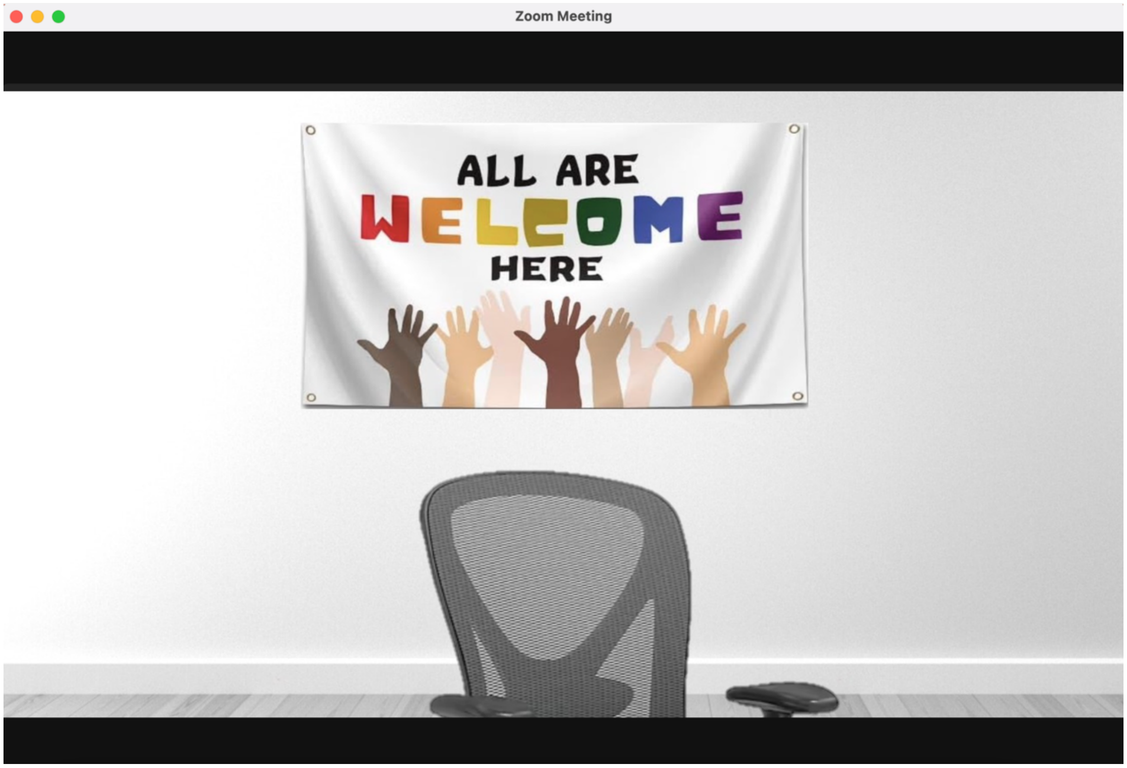

Studies 1-3: American Flag Zoom Background

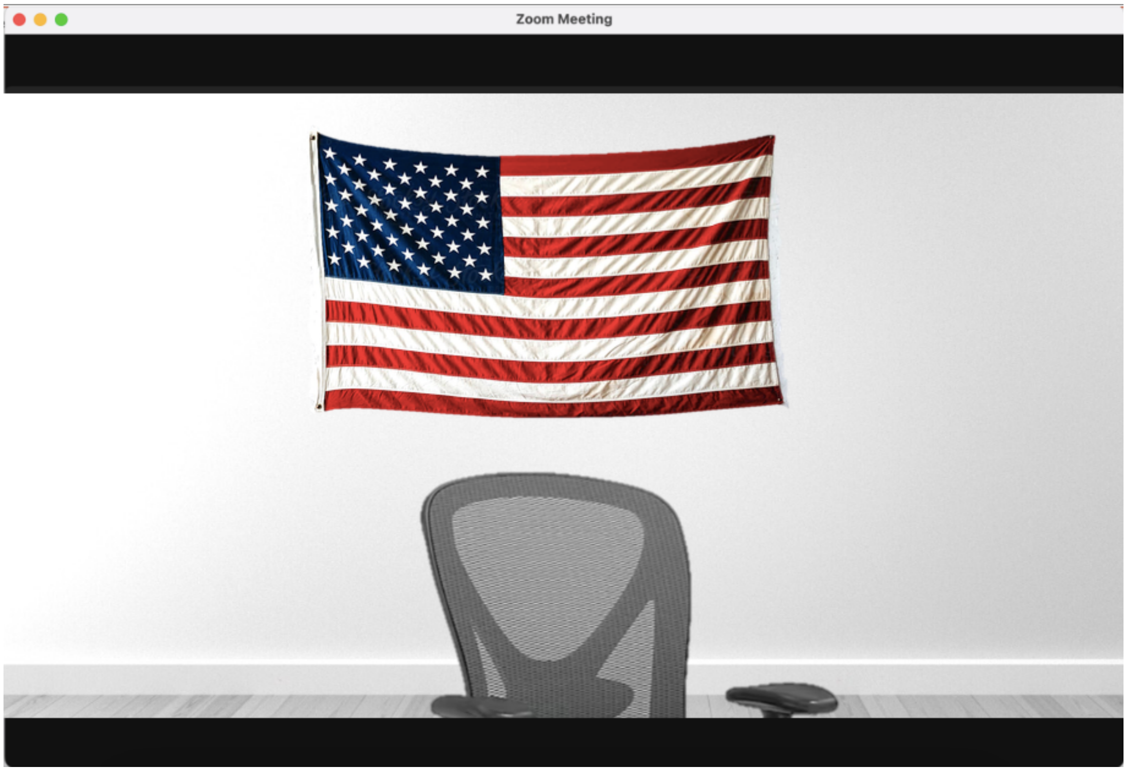

References

Goldstein, N. J., Vezich, I. S., & Shapiro, J. R. (2014). Perceived perspective taking: When others walk in our shoes. *Journal of Personality and Social Psychology*, *106*(6), 941–960. https://doi.org/10.1037/a0036395

Watson, D., Clark, L. A., & Tellegen, A. (1988). Development and validation of brief measures of positive and negative affect: the PANAS scales. *Journal of Personality and Social Psychology*, *54*(6), 1063. http://dx.doi.org/10.1037/0022-3514.54.6.1063
